# Dendritic Pillar[6]Arenes with Fixed Planar Chirality for Stereoselective Inclusions in Water: A Case of Facile Differentiation of Cocaine Adulterants, Levamisole and Dexamisole

**DOI:** 10.1002/anie.202514676

**Published:** 2025-09-01

**Authors:** Nitesh Kumar, Pratik Karmakar, Matthew D. Politeski, Alexandar R. Hansen, Carson E. Ward, Christopher Mortensen, Christopher M. Hadad, Kornkanya Pratumyot, Jovica D. Badjić

**Affiliations:** ^1^ Department of Chemistry and Biochemistry The Ohio State University 100 West 18th Avenue Columbus OH 43210 USA; ^2^ Supramolecular Chemistry Research Unit Department of Chemistry Faculty of Science King Mongkut's University of Technology Thonburi 126 Pracha Uthit Road, Bang Mod, Thung Khru Bangkok 10140 Thailand; ^3^ Campus Chemical Instrument Center The Ohio State University 100 West 18th Avenue Columbus OH 43210 USA

**Keywords:** Chiral shift reagents, Cocaine adulterants, Pillar[6]arene, Planar chirality, Stereoselective recognition

## Abstract

We describe the preparation, conformational dynamics, and stereoselective recognition characteristics of water‐soluble pillar[6]arenes *pS*–**2** ^12−^ and *pR*–**2** ^12−^. These two novel and diastereomeric cavitands comprise a 2,5‐*bis*(ethoxy)pillar[6]arene core with one of six phenylene ring conjugated to two hexaanionic dendrons. Each dendron includes an (*S*)−glutamic acid amidated with two *tris*‐carboxylic Behera's amines. Cavitands *pS*–**2** ^12−^ and *pR*–**2** ^12−^ were obtained in six synthetic steps and resolved by column chromatography. The results of ^1^H NMR and circular dichroism spectroscopic measurements are in line with *pS*/*pR*–**2** ^12−^ having unidirectional orientation of alkoxy substituents (i.e., planar chirality) and no observable interconversion for, at least, 2 weeks. Computational studies supported with ^1^H DOSY NMR measurements revealed that sufficiently bulky dendrons require high activation energy to pass through the pillararene's cylindrical cavity therefore inhibiting rotation of the phenylene holding them. With the unique and chiral binding pocket, *pS*–**2** ^12−^ (*pR*–**2** ^12−^) formed inclusion complexes with cocaine adulterants levamisole and dexamisole (*K*_d_ > mM), with their racemic mixture showing separate ^1^H NMR spectroscopic resonances. In this way, dendritic pillar[6]arenes can be used as chiral shift reagents for determining enantiopurity of pharmaceuticals but also for examining a variety of chiral recognition processes, sensing of chiral molecules, and stereoselective sequestrations in aqueous media.

## Graphical abstract

Dendritic pillar[6]arenes encompass a new class of cavitands with planar chirality, unique binding pockets, and great water solubility. These easily accessible hosts permit differentiating racemic dexamisole from levamisole as known cocaine adulterants. Dendritic pillar[6]arenes can now be used for stereoselective recognition, chemosensing, and sequestration of a broad range of chiral drugs.

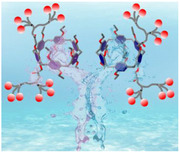

## Introduction

**Figure 1:**
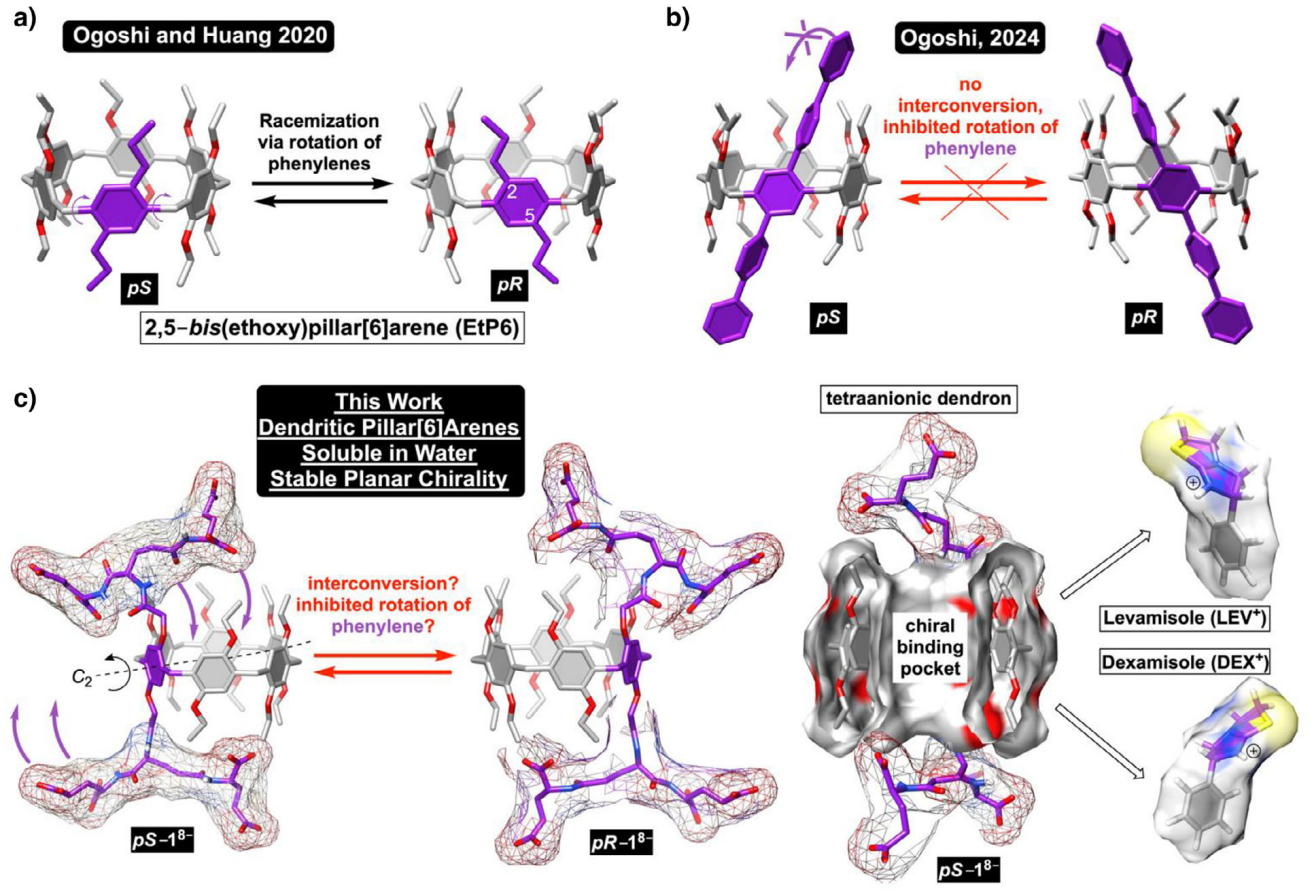
a) A stick representation of *pS* and *pR* stereoisomeric conformers of 2,5‐*bis*(ethoxy)pillar[6]arene (EtP6); as an example, solid state structures of EtP6 reported by the groups of Ogoshi (2020) and Huang (2020) show steric gearing and conformational bias (see Refs. [6, 7]). b) A stick representation of *pS* and *pR* stereoisomers of A1/A2 arylated pillar[6]arene. These two enantiomers are not interconverting at a room temperature and can be separated by chiral HPLC chromatography; see Ref. [38]. c) We hypothesized that placing bulky anionic dendrons, comprising natural α‐amino acids (*tris*‐Glu^4−^, OPLS4), at top and bottom of pillar[6]arene could improve water solubility and prevent the interconversion of diastereomeric *pS*–**1** ^8−^ to *pR*–**1** ^8−^ by inhibiting rotation of the phenylene holding the dendrons. Right: Surface area of *pS*–**1** ^8−^ is shown with energy‐minimized and complementary structures (DFT: B3LYP, 6‐31G*) of cocaine adulterants, levamisole and dexamisole.

**Scheme 1:**
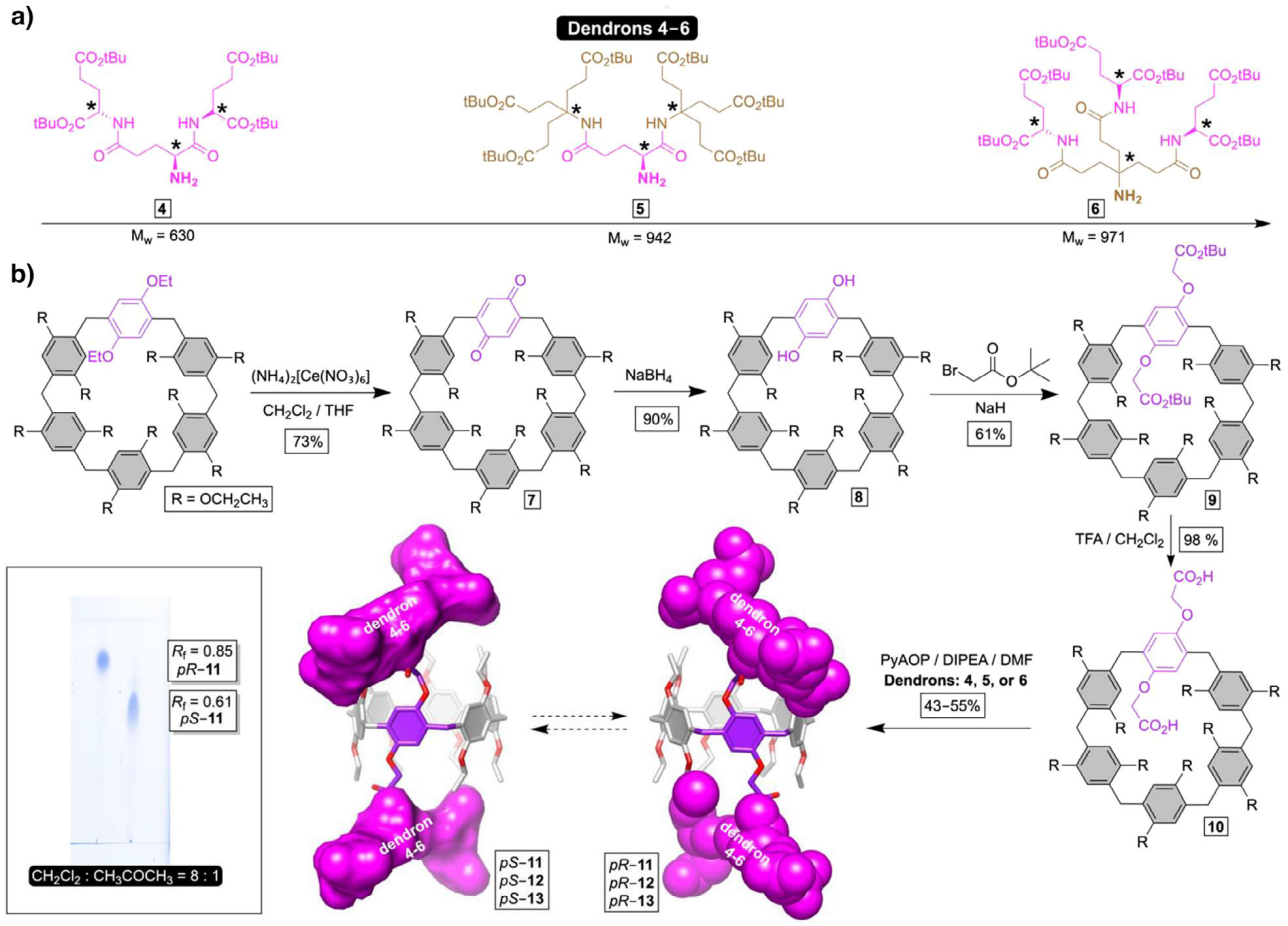
a) Chemical structures of chiral dendrons **4**–**6** along with their molecular weights and starred branching points. b) Synthesis of dendritic pillar[6]arenes *pS*/*pR*–**11**, *pS*/*pR*–**12**, and *pS*/*pR*–**13**. Left: A recorded image of thin‐layer chromatographic plate (visualized with cerium ammonium molybdate stain) shows well separated spots from diastereomeric *pS*–**11** and *pR*–**11** along with their *R*_f_ values.

Pillararenes are pillar‐shaped cavitands composed of phenylenes linked at their *para* positions via methylene groups and holding two alkoxy substituents at positions 2 and 5 (Figure 1a).^[^ ^1^ ^]^ As a result of steric gearing,^[^ ^2^ ^]^ pillar[5]or[6]arenes assume two principal conformations^[^ ^3^ ^]^ in which alkoxy units align at upper and bottom rims^[^ ^4^ ^]^ to give rise to a pair of interconverting macrocycles with *D*_n_ symmetry and planar *pR* and *pS* chirality (Figure 1a).^[^ ^5^, ^6^, ^7^ ^]^ Chiral pillararenes^[^ ^8^, ^9^ ^]^ have thus been probed for creating a variety of chiroptical switches^[^ ^10^ ^]^ in which external input (e.g., heat, guests, electrons, or light)^[^ ^11^, ^12^, ^13^, ^14^ ^]^ triggers a chiroptical output via change in planar chirality (i.e., *pS* to *pR* or vice versa). Furthermore, enantioselective inclusion of α‐amino acids was studied with water soluble and racemic pillar[5/6]arenes:^[^ ^15^, ^16^, ^17^ ^]^ the inclusion of chiral molecule altered the population of dynamic *pR* or *pS* cavitands resulting in chiroptical response (i.e., circularly polarized absorption or luminescence) to report on the presence of (*R*) or (*S*)‐amino acids in solution. And last, *pS*/*pR*‐pillararenes have been turned into a) circularly polarized luminescent (CPL) materials^[^ ^18^ ^]^ for applications in the area of chemical sensing,^[^ ^19^, ^20^ ^]^ b) homochiral metal‐organic frameworks for stereoselective separation of chiral molecules,^[^ ^21^, ^22^, ^23^ ^]^ c) ligands for transition metal asymmetric catalysis,^[^ ^24^ ^]^ d) nanochannels for controlling molecular trafficking across membranes,^[^ ^25^, ^26^ ^]^ and e) sequestering agents for selective removal of toxic compounds from living systems.^[^ ^27^, ^28^, ^29^ ^]^ Importantly, a facile interconversion (i.e., racemization)^[^ ^3^ ^]^ of *pR* to *pS* enantiomers of *C*_5_/*C*_6_ symmetric pillar[5/6]arenes holding linear alkoxy chains^[^ ^30^ ^]^ (Figure 1a) ought to be slowed down to allow their physical separation (i.e., resolution). So far, one can a) introduce bulky groups at both rims to prevent the rotation of all phenylenes through cylindrical cavity,^[^ ^4^ ^]^ b) thread a linear molecule through cavity to obtain *pR* and *pS* catenanes^[^ ^31^, ^32^ ^]^ or rotaxanes,^[^ ^33^ ^]^ and c) conjugate sufficiently sizeable and rigid aromatics to one of phenylenes^[^ ^34^, ^35^, ^36^ ^]^ from pillar[5]arenes (i.e., A1/A2 arylation)^[^ ^37^ ^]^ to inhibit the racemization via inversion. With all studies centered on resolution of pillar[5]arenes^[^ ^5^ ^]^ and, to our knowledge, single report^[^ ^38^ ^]^ addressing the separation of *pR*/*pS* enantiomers pillar[6]arenes via A1/A2 arylation for studies in organic media (Figure 1b), we wondered about developing a novel but also general methodology for inhibiting the racemization of the latter cavitands capable of forming stable inclusion complexes with functional drugs in water.^[^ ^27^, ^39^, ^40^, ^41^, ^42^ ^]^ In particular, we hypothesized (Figure 1c) that double amidation of pillar[6]arenes with bulky dendrons composed of α‐amino acids^[^ ^43^ ^]^ could prevent ring rotation and give a pair of noninterconverting and diastereomeric cavitands of type *pS*/*pR*–**1** ^8−^; tetraanionic dendron *tris*‐Glu^4−^ (derived from 4 in Scheme 1a) within *pS*/*pR*–**1** ^8−^ was recently introduced by our laboratory^[^ ^43^ ^]^ for preventing self‐inclusion of molecular baskets in water. Diastereomeric *C*_2_ symmetric hosts of type *pS*/*pR*–**1** ^8−^ were expected to be accessible in large quantity, resolvable by column chromatography, and soluble in water for selective inclusion complexation of chiral drugs. On that note, placing a branched, anionic, and peptidic dendron on the top and bottom rims of a deep nonpolar *pS* or *pR* cavity of pillar[6]arene akin in size to β/γ‐cyclodextrins^[^ ^44^ ^]^ and lined with alkoxy groups grants a unique binding pocket (Figure 1c) with functional dendrons permitting water solubility and potentially serving as molecular recognition units. Known cocaine adulterants, levamisole and dexamisole (i.e., tetramisole),^[^ ^45^ ^]^ among other cationic and racemic drugs,^[^ ^46^ ^]^ were hypothesized to complement the inner space of such chiral cavitands^[^ ^47^ ^]^ thereby allowing ^1^H NMR spectroscopic differentiation (Figure 1c).^[^ ^48^ ^]^ With the goal of developing novel and dendritic pillar[6]arenes with stable planar chirality and solubility in water, we herein describe a study of their synthesis, resolution, and stereoselective recognition^[^ ^49^, ^50^ ^]^ of tetramisole.

## Results and Discussion

### Design Principles

By appending linear alkoxy chains having 2–12 carbons to pillar[5]arene (Figure 1a), Ogoshi and coworkers found^[^ ^30^ ^]^ that the rate of its *pS*/*pR* racemization slows down with longer chains. Importantly, the activation energy characterizing the interconversion^[^ ^30^ ^]^ was insufficient (Δ*G* ^‡^ <14 kcal mol^−1^) to prevent a physical separation of enantiomers.^[^ ^3^ ^]^ On the contrary, placing cyclohexylmethyl groups (i.e., CH_2_C_6_H_11_) at both rims of pillar[5]arene allowed for facile resolution of *pS*/*pR* enantiomers with chiral HPLC chromatography.^[^ ^4^ ^]^ While methylcyclohexane is 149 Å^3^ in volume and smaller than dodecane 234 Å^3^, the cyclic ring is conformationally restricted with a thicker profile so that its slippage^[^ ^51^ ^]^ through the cylindrical cavity of the pillar[5]arene (c.a. 5 Å in diameter)^[^ ^52^ ^]^ must be causing a considerable van der Waals strain to inhibit the racemization via inversion. Indeed, the process in which a narrow molecular “passage” restricts translation of molecules was originally recognized by Cram as constrictive binding^[^ ^53^, ^54^ ^]^ leading to investigations of molecular gating^[^ ^55^, ^56^ ^]^ and mechanically interlocked compounds.^[^ ^53^ ^]^ While examining the kinetic stability of (anion ⊂ hexapodal capsule) inclusion complexes,^[^ ^57^ ^]^ we recently found that the shape of anions played a role in the rate by which they access the inner space of capsules: trigonal planar and cylindrical anions make their way through the narrow aperture of hexapodal capsules at a faster rate than the tetrahedral ones with a thicker profile. Along with the notion of constrictive binding in a variety of molecular environments,^[^ ^58^ ^]^ we reasoned that ring inversions within pillar[6]arene possessing a sizeable cylindrical cavity (ca. 6.7 Å in diameter)^[^ ^52^ ^]^ may be inhibited with dendron **4** ^[^ ^43^ ^]^ (Scheme 1a) composed of three (*S*)‐glutamic acids. While the branching of conformationally flexible dendron **4** provides bulkiness, the presence of six stereogenic centers renders corresponding *pS*–**11** and *pR*–**11** hosts diastereomeric (Scheme 1b), to be a subject of resolution by standard column chromatography.^[^ ^21^ ^]^ As an alternative, we also chose to probe the conjugation of two larger and chiral dendrons **5** and **6** (Scheme 1a). Dendron **5** comprises two carboxylates from (*S*)‐glutamic acid connected to branched Behera's amines^[^ ^59^, ^60^ ^]^ while dendron **6** includes Behera's amine holding three (*S*)‐glutamic acids; for syntheses of **4**–**6**, see Scheme S2. The bulkiness of dendrons **4**‐to‐**6** increases as indicated by greater molecular weight and number of branching points along the series (Scheme 1a).

### Synthesis of Dendritic Pillar[6]Arenes *pS*/*pR*–11/12/13

The oxidation of 2,5‐diethoxybenzene unit from abundant 2,5‐*bis*(ethoxy)pillar[6]arene (**EtP6**) with cerium(IV) ammonium nitrate gave pillar[6]arene[1]quinone **7** in 73% yield (Scheme 1b).^[^ ^52^ ^]^ Next, two‐electron reduction of the quinone from **7** into hydroquinone‐containing **8** was completed with NaBH_4_, which was then used as a nucleophile to, in the reaction with *tert*‐butyl 2‐bromoacetate, give pillar[6]arene **9** (61% yield).^[^ ^61^ ^]^ Finally, trifluoroacetic acid promoted deprotection of **9** led to the formation of dicarboxylic acid functionalized pillar[6]arene **10** ready for peptide coupling with dendrons **4**–**6**. After each amidation, promoted with PyAOP coupling reagent, dendritic pillar[6]arenes *pS*/*pR*–**11**, *pS*/*pR*–**12**, and *pS*/*pR*–**13** were obtained in 43%–55% yields (Schemes 1b and S3; Figures S37–S47). Note that for all three cavitands, ^1^H NMR spectra of crude products revealed two sets of signals corresponding to *pS* and *pR* diastereomers in a comparable ratio.

### Diastereomeric Pillar[6]Arenes *pS*/*pR*–1^8−^/2^12−^/3^12−^

**Figure 2:**
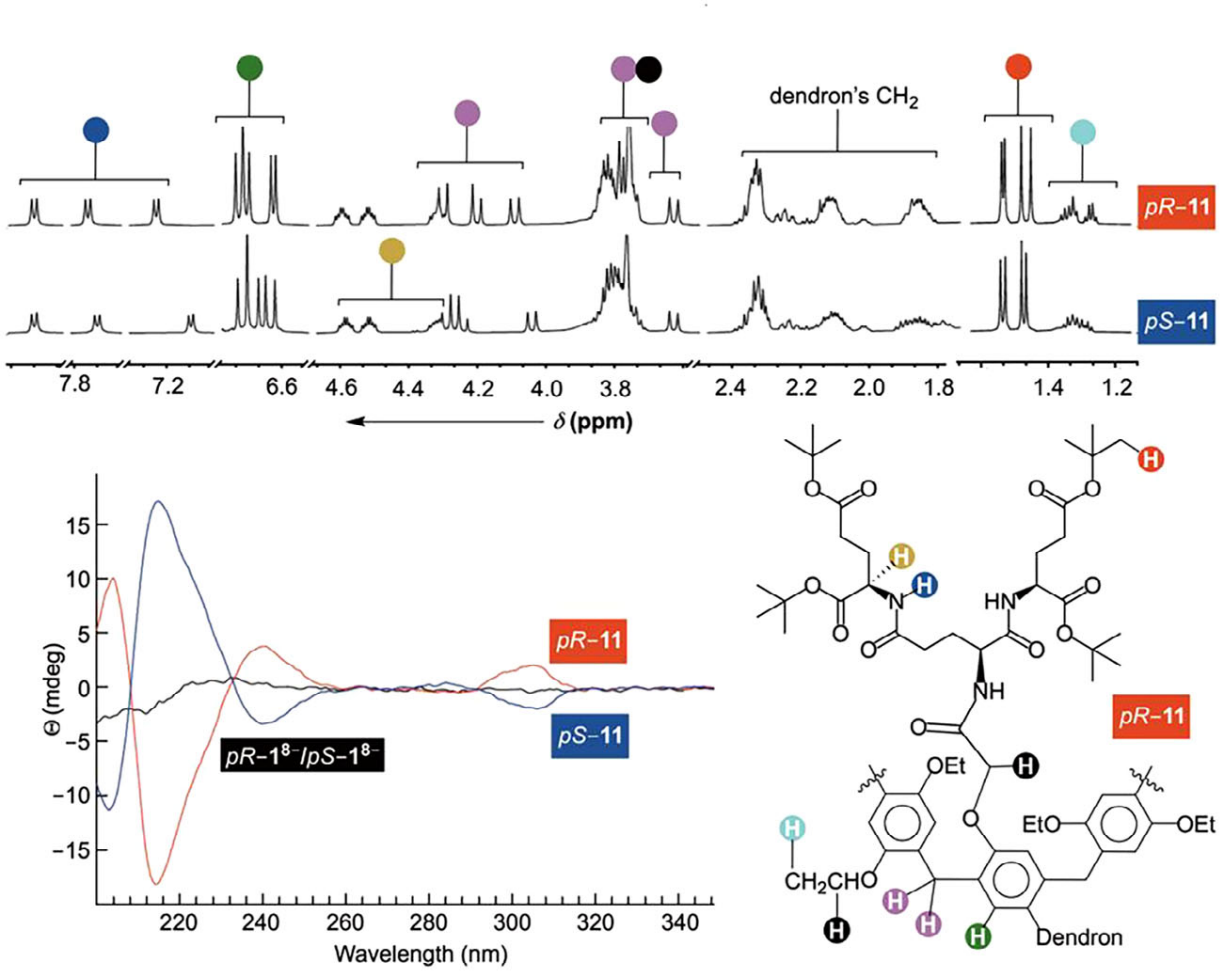
^1^H NMR spectrum (600 MHz, 298.0 K) of dendritic pillar[6]arenes *pR*–**11** (top) and *pS*–**11** (bottom) in CD_2_Cl_2_ with color‐coded assignment of signals. Chemical structure of *pR*‐**11** is shown at the bottom right. Circular dichroism (CD) spectra of 25 µM *pR*–**11** (red) and *pS*–**11** (blue) in CH_3_OH. CD spectrum of 50 µM *pS*/*pR*–**1^8^** ^−^ in 30 mM phosphate buffer at pH = 7.0 is shown in black.

With ^1^H NMR spectrum of crude *pS*/*pR*–**11** showing two sets of resonances, we presumed that two dendritic cavitands could be interconverting slowly on the ^1^H NMR time scale. After a solution of diastereomeric mixture was examined by thin‐layer chromatography, two separate spots were observed having *R*_f_ values of 0.61 and 0.85 (Scheme 1b).^[^ ^21^ ^]^ It follows that the free energy of activation corresponding to interconversion of *pS*–**11** and *pR*–**11** ought to be sufficiently high to permit chromatographic separation. That is to say, if it takes 10 min to develop a chromatographic plate showing two distinct spots or 1 h to run a column, then one can conservatively estimate *t*_1/2_ > 1 h for the conversion of one stereoisomer to another. By computing first‐order rate coefficient *k* from *t*_1/2_ = ln(2)/*k* at 298 K, one can use the Eyring equation to find Δ*G* ^‡^ >23 kcal mol^−1^. After the resolution of *pS*–**11** and *pR*–**11** by column chromatography, ^1^H NMR spectrum of each compound was found to be in line with *C*_2_ symmetric molecule (Figure 2 and S37–S40). Each stereoisomer showed 6 singlets from 12 aromatic protons (*δ* = 6.6–6.8 ppm, green) as well as 3 pairs of AB quartets from 6 diastereotopic methylene units (*δ* = 3.7–4.4 ppm, pink) connecting the aromatics. Furthermore, variable temperature (VT) ^1^H NMR spectra of *pS*–**11** in CD_2_Cl_2_ (Figure S50) revealed an extensive broadening and, seemingly, absence of new resonances at lower temperatures (298.0–208.0 K). It follows that less symmetric conformers of *pS*–**11** were missing from conformational equilibria, provided that, at lower temperatures, they exchanged at a slow rate with *C*_2_ symmetric *pS*–**11**. Indeed, VT ^1^H NMR spectra of **2**,**5**‐*bis*(ethoxy)pillar[6]arene (**EtP6**) in both C_6_D_5_CD_3_ and CD_2_Cl_2_ had resonances from diastereotopic CH_2_ protons undergo decoalescence at 180–190 K (Figure S49)^[^ ^4^, ^30^ ^]^ with ring rotations occurring slow on the ^1^H NMR time scale. The notion that dialkoxy aromatics in *pS*–**11** are geared in the same direction is supported from studies of a variety of pillar[5/6] arenes, both in solution and solid state.^[^ ^3^ ^]^ Circular dichroism spectra of diastereomeric *pS*–**11** and *pR*–**11** were mirror image of one another (Figure 2). By using CD spectra of already studied pillararenes,^[^ ^62^ ^]^ the faster moving fraction of dendritic **11** (*R*_f_ = 0.85, Scheme 1b) had a positive Cotton effect at 310 nm and we assigned it as *pR*.^[^ ^63^ ^]^ The slower moving diastereomer with the negative CD band at 310 nm was thus *pS*–**11**.^[^ ^38^ ^]^ For removing four tertiary‐butyl (*t*‐Bu) groups from *pS*–**11** and therefore rendering dendritic pillararene soluble in aqueous media, we used an excess of trifluoroacetic acid (Scheme S4). After dissolving the alleged *pS*–**1** in 30 mM phosphate buffer at pH = 7.0, ^1^H NMR spectrum (Figures S51 and S52) revealed the presence of both *pS*–**1** ^8−^ and *pR*–**1** ^8−^ in roughly equal ratio. Likewise, CD spectrum of the product was hardly distinguishable from the baseline (black line, Figure 2). Evidently, dendron **4** without four *t*‐Bu groups lacked bulkiness^[^ ^64^ ^]^ for preventing the interconversion of stereoisomeric pillar[6]arenes. The anionic *tris*‐Glu^4−^ groups make their way through the cylindrical cavity of the cavitand to enable a facile interconversion of *pS*–**1** ^8− ^and *pR*–**1** ^8−^; if *t*_1/2_ for *pS*‐to‐*pR* is <1 min, Δ*G* ^‡^ <20 kcal mol^−1^. On the positive note, 2 tetraanionic and peptidic dendrons rendered *pS*/*pR*–**1** ^8−^, holding nonpolar 6 benzene rings with 10 ethoxy groups, soluble in water.

**Figure 3:**
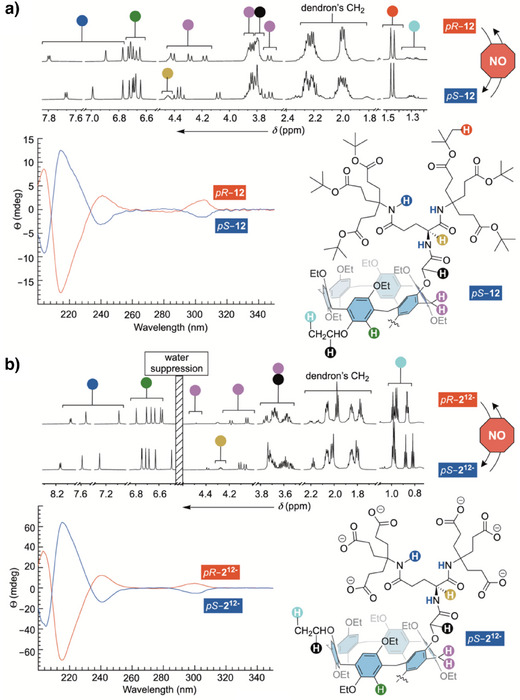
a) ^1^H NMR spectrum (600 MHz, 298.0 K) of dendritic pillar[6]arenes *pR*–**12** (top) and *pS*–**12** (bottom) in CD_2_Cl_2_ with color‐coded assignment of signals. Chemical structure of *pR*–**12** is shown at the bottom right. CD spectra of 25 µM *pR*–**12** (red) and *pS*–**12** (blue) in CH_3_OH. b) ^1^H NMR spectrum (600 MHz, 298.0 K) of dendritic pillar[6]arenes *pR*‐**2** ^12−^ (top) and *pS*–**2** ^12−^ (bottom) in 30 mM phosphate buffer at pH = 7.0. All ^1^H NMR resonances are color‐coded with chemical structure of *pS*‐**2** ^12−^ shown at the bottom right. CD spectra of 100 µM *pR*–**2** ^12−^ (red) and *pS*–**2** ^12−^ (blue) in 30 mM phosphate buffer at pH = 7.0.

Will more sizeable dendrons **5** (Scheme 1a), act differently and inhibit *pS*–**12** to *pR*–**12** interconversion after deprotection? As in the case of diastereomeric *pS*/*pR*–**11**, we separated *pS*–**12** from *pR*–**12** using column chromatography. ^1^H NMR spectrum of each cavitand (Figures 3a and S41–S44) was in line with *C*_2_ symmetric molecule having all alkoxy groups geared in the same direction. CD spectra of *pS*–**12** and *pR*–**12** (Figure 3a) were mirror image of one another and almost identical to *pS*–**11** and *pR*–**11** therefore corroborating similar conformational characteristics of two pillar[6]arene frameworks. After the removal of *t*‐Bu groups, *pS*–**12** and *pR*–**12** were changed into water soluble *pS*–**2** ^12−^ and *pR*–**2** ^12−^ (Figures 3b and S53–S56). ^1^H NMR spectrum of each deprotected molecule showed a distinct set of signals that remained unchanged over a period of 2 weeks at 50 °C (Figure S62); with an assumption that *t*_1/2_ > 2 weeks, Δ*G* ^‡^ > 28 kcal mol^−1^ at 348 K. For *pS* and *pR*–**2** dissolved in DMSO, ^1^H NMR spectrum remained unchanged for 60 days at room temperature and after heating for 1 h at 150 °C (Figure S62). CD spectra of *pS*–**2** ^12−^ and *pR*–**2** ^12−^ (Figure 3b) confirmed that chiral characteristics of each diastereomer are in polar aqueous media similar to those observed in organic solvent (Figure 3a). Finally, *pS*–**2** ^12−^ and *pR*–**2** ^12−^ are stable stereoisomers not interconverting into one another at ambient conditions and in water with millimolar solubility (Figure S63). The stage was set to study the capacity of such unique hosts for promoting inclusion complexation of drugs.

Resolving stereoisomeric and dendritic *pS*/*pR*–**13** pillararenes (Figures S45 and S46) by silica gel chromatography was unsuccessful, despite testing a variety of mobile phases. While there may be a facile way to separate these cavitands, we decided to focus on examining already accessible *pS*/*pR*–**2** ^12−^.

### Computational Study of *pS*‐to‐*pR* Interconversion Within Dendritic Pillar[6]Arenes

**Figure 4:**
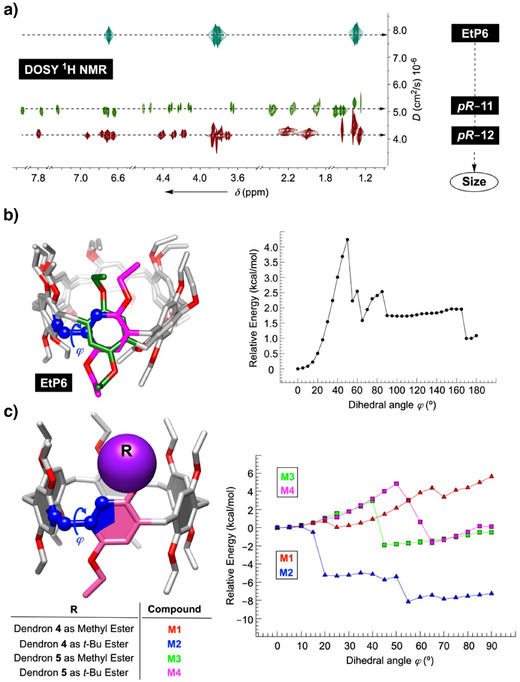
a) DOSY ^1^H NMR spectra (600 MHz, 298.0 K) of **EtP6** (1.5 mM), *pR*–**11** (1.5 mM) and *pR*–**12** (1.5 mM) in CD_2_Cl_2_ (Figure S48). Change in intensities of individual peaks over the magnetic field gradient strength were fit to Stejskal–Tanner equation to obtain diffusion coefficients (Mnova software) that were then converted into hydrodynamic radii (*R*_H_) using the Stokes–Einstein equation. b) Ball and stick representations of initial (pink) and final (green) poses of **EtP6** during dihedral drive scan about dihedral angle *j* (shown in blue). Right: Potential energy diagram for dihedral drive scan (PM6) of **EtP6**. c) Ball and stick representation of dendritic pillar[6]arenes **M1**‐**M4**, each holding one dendron of type **4** or **5** at the top rim. Right: Potential energy diagram for dihedral drive calculations (PM6) of dendritic pillar[6]arenes **M1** (red), **M2** (blue), **M3** (green), and **M4** (pink).

The interconversion of *pS* to *pR* stereoisomers of pillar[5/6]arenes occurs by a geared rotation^[^ ^2^ ^]^ of five or six phenylene rings.^[^ ^65^ ^]^ The process encompasses an intricate potential energy surface^[^ ^5^ ^]^ that, based on gearing in molecular baskets,^[^ ^66^ ^]^ may be investigated using a combination of nudge elastic band and density functional theory computations. As for dendritic pillar[6]arenes, we decided to examine the stereoisomerization by completing a series of dihedral drive studies (PM6 level of theory, Figure 4b,c)^[^ ^67^ ^]^ in which only one of the phenylenes is rotated about its axis. The energy profile for the rotation (i.e., degree of rotation as a function of potential energy) was expected to shine light on the bulkiness of alkoxy groups and their behavior going from 2,5‐*bis*(ethoxy)pillar[6]arene (**EtP6**, Figure 1a) to *pS*/*pR*–**11** and *pS*/*pR*–**12** (Scheme 1).

In this regard, DOSY ^1^H NMR measurements of EtP6, *pR*–**11**, and *pR*–**12** (Figures 4a and S48) in nonpolar dichloromethane, revealed an increase in hydrodynamic radii (*R*_H_) along the series. Thus, EtP6 holding twelve ethoxy groups at its rims moved at the fastest rate in liquid phase with *R*_H_ = 6.50  ± 0.05 Å. For *pR*–**11** holding two dendrons of type **4** at its rims, *R*_H_ was found to be 10.0 ± 0.05 Å. Finally, the diffusion of *pR*–**12** with two dendrons of type **5** was the slowest with *R*_H_ = 12.2 ± 0.4 Å. The notion that the size of the alkoxy arms increases from **EtP6** to *pR*–**11** and then *pR*–**12** bodes well with the facile *pS*‐to‐*pR* interconversion for **EtP6** and deprotected *pR*–**11** (i.e., *pR*–**1**) while its absence for *pR*–**12** and *pR*–**2**.

For energy optimized **EtP6** (PM6), we set the dihedral drive scan to take place by single phenylene (pink, Figure 4b) rotating about dihedral angle *φ* (blue, Figure 4b) in 36 steps at 5° increments. The initial and final poses of **EtP6** display a full rotation of the ring (pink‐to‐green, Figure 4b) in which the formation of diastereomeric (*pS*)_5_(*pR*)‐EtP6 from (*pS*)_6_‐EtP6 ensues. The activation energy *E*_a_ (Figure 4b) is circa 4.2 kcal mol^−1^ with the ethoxy group easily threading through the cavitand's cylindrical cavity and thereby causing a negligible van der Waals steric strain (Figure S49). On the contrary, the full rotation of phenylene within dendritic pillar[6]arenes **M3** and **M4** holding single dendron of type **5** (akin to *pS*–**12**, Figure 4c) failed at *φ* > 90°. At smaller dihedral angles, the potential energy increased sharply only to drop at later stages with the system finding other local energy minima. In the case of **M1** and **M2** pillar[6]arenes holding single dendron of type **4** (akin to *pS*–**11**, Figure 4c) the full rotation also failed at *φ* > 90° (Figure 4c). Importantly, lower slopes of **M1**/**M2** than **M3**/**M4** potential energy curves (Figure 4c) imply that the insertion of less bulky dendron **4** in the cavity of pillar[6]arene causes a smaller van der Waals strain than bigger dendron **5**.

### Inclusion Complexation of Levamisole and Dexamisole with Dendritic *pS*–2^12−^ and *pR*–2^12−^

**Figure 5:**
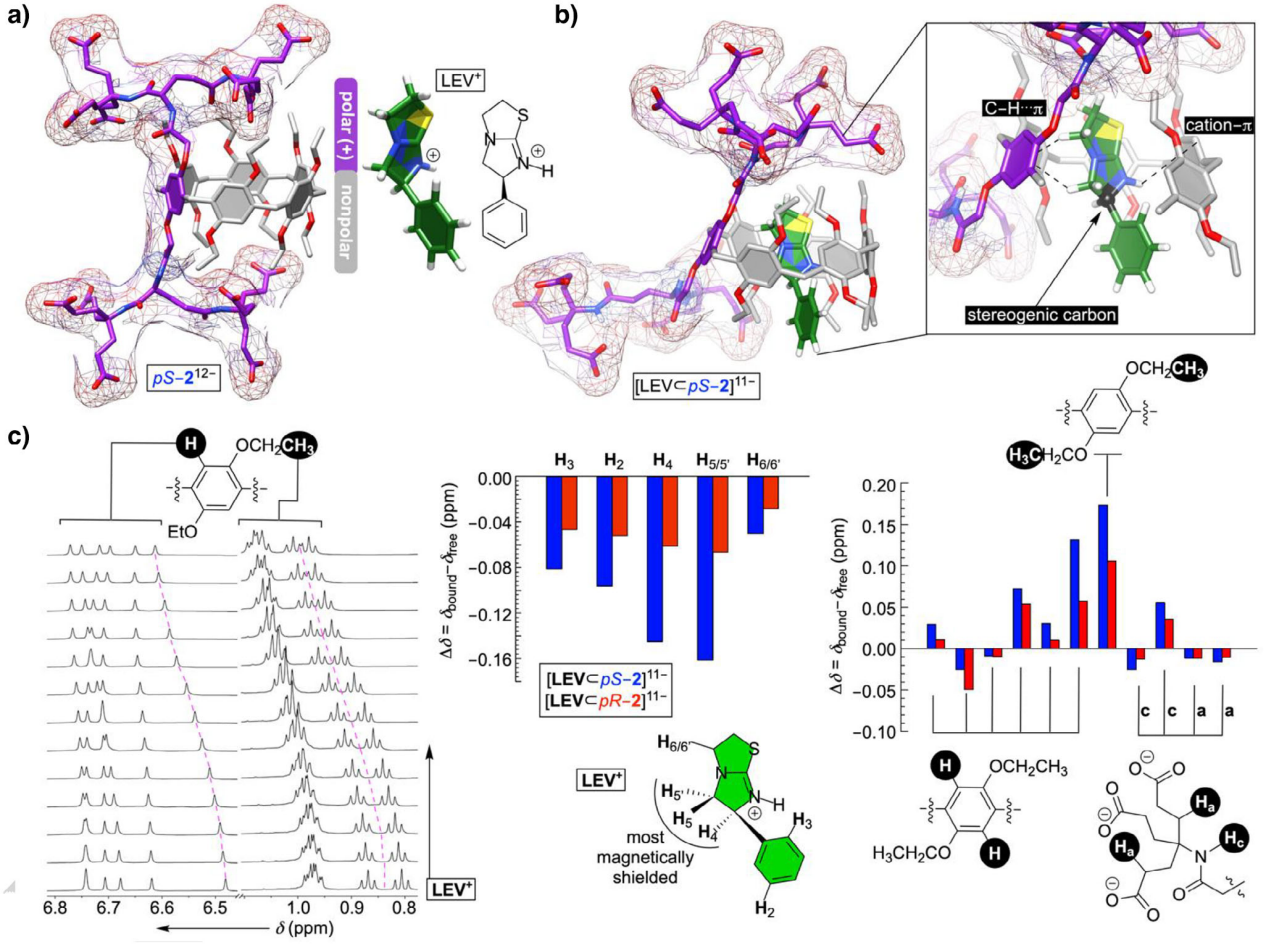
a) A stick representation of energy‐minimized structures of *pS*–**2** ^12−^ (OPLS4) and levamisole (LEV^+^, DFT: B3LYP, 6–31G*). b) The most stable pose from Monte‐Carlo conformational search of [LEV ⊂ *pS*–**2**]^11−^ in implicit water solvent (OPLS4, Figure S70). c) A section of ^1^H NMR spectra (600 MHz, 298.0 K) of 0.25 mM dendritic pillar[6]arene *pS*–**2** ^12−^ (30 mM phosphate buffer at pH = 7.0) obtained after incremental addition of 50 mM solution of LEV^+^ in 30 mM phosphate buffer at pH = 7.0 (see also, Figure S65); for supramolecular titration of LEV^+^ to *pR*–**2** ^12−^, see Figure S67. Left bar plot: Chemical shift perturbations (Δ*δ*  = *δ*_bound_ – *δ*_free_) of ^1^H NMR resonances from LEV^+^ during the formation of [LEV ⊂ *pS*–**2**]^11−^ (blue) and [LEV ⊂ *pR*–**2**]^11−^ (red) were obtained from titration experiments (Figures S65–S68). Right bar plot: Chemical shift perturbations (Δ*δ* = *δ*_bound_ – *δ*_free_) of ^1^H NMR resonances from *pS*‐**12** (blue) and *pR*‐**12** (red) during the formation of [LEV ⊂ *pS*–**2**]^11−^ and [LEV ⊂ *pR*–**2**]^11−^, respectively, were obtained from titration experiments (Figures S65–S68); note that for six aromatic signals, we arbitrarily paired the observed Δ*δ* values from blue versus red resonances, since we could not fully assign them.

^1^H NMR spectra of 37–250 µM dendritic *pS*–**2** ^12−^ in water (30 mM PBS at pH = 7.0) showed a negligible change of chemical shifts of its resonances and their line widths (Figure S60). Moreover, ^1^H DOSY NMR of *pS*–**2** ^12−^ (250 µM) revealed all of the resonances having equal diffusion coefficient (*D* = 1.95  ± 0.03·10^−9^ cm^2^ s^−1^; Figure S61 and Table S4). After converting this value into hydrodynamic radius *R*_H_ = 12.1  ± 0.4 Å, using the Stokes–Einstein equation, it matched the size of energy‐minimized *pS*–**2** ^12−^ having longest distances across equal to *R*_H_ = 13–14 Å (OPLS4, Figure 5a). It follows that bolaamphiphilic *pS*–**2** ^12− ^is, at concentrations <0.25 mM in water, predominantly in its monomeric state. For probing the capacity of such monomeric *pS*–**2** ^12−^ to recognize and differentiate chiral organic molecules with ^1^H NMR spectroscopy,^[^ ^48^ ^]^ we considered a number of easily available and enantiopure drugs.^[^ ^46^ ^]^ The hypothesis was that water soluble pharmaceuticals with a cationic site at top of a nonpolar aromatic group would occupy the chiral and nonpolar cavity of dendritic pillar[6]arene while positioning the cationic moiety next to one of anionic dendrons (Figure 5a).

Levamisole (LEV^+^, Figure 5a) is a veterinary medicine (Ergamisol) used for treating parasitic worm infections (anthelmintic) and more recently as an adulterant in illicitly distributed cocaine^[^ ^68^ ^]^ with life‐threatening effects.^[^ ^69^ ^]^ The drug comprises a phenyl ring linked to cationic tetrahydro imidazothiazole with (*S*)‐stereogenic carbon; with p*K*_a_ = 6.75–6.98, circa 50% of LEV is at pH = 7.0 in the cationic LEV^+^ form.^[^ ^70^ ^]^ In terms of both size and electronic characteristics,^[^ ^27^ ^]^ LEV^+^ seemed complementary to our dendritic pillar[6]arenes (Figure 5a). Dexamisole is (*R*)‐enantiomer of the drug (DEX^+^) without anthelmintic although with antidepressant characteristics.^[^ ^71^ ^]^ Importantly, both enantiopure LEV^+^ and the racemic mixture of LEV^+^/DEX^+^ (i.e., tetramisole) have been found in blood samples of cocaine users.^[^ ^72^, ^73^ ^]^ We wondered: can ^1^H NMR spectroscopy be used to distinguish LEV^+^ from DEX^+^ while residing in the inner space of dendritic *pS*–**2** ^12−^ or *pR*–**2** ^12−^ pillar[6]arenes?^[^ ^71^ ^]^ If so, can this method be applied to rapidly quantify the ratio of two stereoisomeric drugs in aqueous samples?^[^ ^74^ ^]^ A Monte‐Carlo conformational search (OPLS 4, Maestro) of LEV^+^ docked in *C*_2_ symmetric cavity of *pS*–**2** ^12−^ revealed a set of comparable poses dominating the energy landscape (<1.4 kcal mol^−1^, Figures 5b and S70). The drug occupies nonpolar cavity of pillar[6]arene so that positively charged imidazothiazole engages in cation−π interactions with aromatics from pillar[6]arene.^[^ ^75^ ^]^ Notably, (*S*) stereogenic carbon from LEV^+^ sits within unidirectional (i.e., *pS*) belt of aromatics wherein it could potentially be distinguished from the opposite (*R*) stereoisomer via host–guest intermolecular contacts. While one of the anionic dendrons lodges away from the cavity the other one is on top with its carboxylates >7 Å from the drug's formal cationic site.

An incremental addition of a standard solution of LEV^+^ to *pS*–**2** ^12−^ in water (30 mM phosphate buffer at pH = 7.0) was monitored with ^1^H NMR spectroscopy (Figures 5c, left and S65). A steady perturbation of resonances from both compounds suggested noncovalent interactions taking place in solution. A change in the chemical shift of protons from *pS*–**2** ^12−^ as a function of increasing concentration of LEV^+^ fit well to 1:2 stoichiometric model (via nonlinear regression analysis, Figure S66)^[^ ^76^ ^]^ with *K*_1_ = 222 ± 1 M^−1^ and *K*_2_ = 22 ± 1 M^−1^. Results from mass spectrometry measurements corroborated the finding (Figure S66), implying the drug occupying the cavity of *pS*–**2** ^12−^ in addition to, we posit,^[^ ^77^, ^78^ ^]^ binding to its nonpolar outer surface. Similarly, supramolecular titration of LEV^+^ to stereoisomeric *pR*–**2** ^12−^ was also in line with the formation of binary (*K*_1_ = 176 ± 1 M^−1^) and ternary (*K*_2_ = 48 ± 1 M^−1^) complexes (Figures S67 and S68). Allegedly, the poor stereoselectivity^[^ ^49^ ^]^ resulted from weak binding and small number of intermolecular host–guest contacts.^[^ ^79^ ^]^ With the first binding event being sufficiently stronger than the second, our further discussion will center on the formation of binary [LEV ⊂ *pS*–**2**]^11−^ and [LEV ⊂ *pR*–**2**]^11−^ complexes. The magnetic shielding of all resonances from LEV^+^ within [LEV ⊂ *pS*–**2**]^11−^ (Figure 5c, middle) are in line with the drug included in diamagnetic shielding region of the aromatic cavity of the host. That is to say, H_4_ and H_5_ resonances from LEV^+^ experienced the greatest chemical‐shift change (Δ*δ* = *δ*_bound_ − *δ*_free_) to be in line with their computed positioning against the cavitand's aromatics while engaging in cation−π interactions (Figure 5b). On the other side, the same trend in Δ*δ* values from LEV^+^ occupying the *pR*–**2** ^12−^ and *pS*–**2** ^12−^ cavitands (Figure 5c, middle) suggested comparable binding poses for diastereomeric [LEV ⊂ *pS*–**2**]^11−^ and [LEV ⊂ *pR*–**2**]^11−^ complexes. As for protons from pillar[6]arenes *pR*–**2** ^12−^ and *pS*–**2** ^12−^ during ^1^H NMR titrations, the resonances from 10 methyl groups (Figure 5c, right) showed the greatest degree of change (i.e., deshielding, Δ*δ*  = 0.10–0.17 ppm). Presumably, nonpolar ethoxy groups are tucked in the aromatic pillararene's cavity in polar water solvent and thus magnetically shielded.^[^ ^80^ ^]^ After the inclusion of LEV^+^, the ethoxy groups move to bulk solvent to reside in less‐shielded environment. This conformational change could be a part of the reason for the observed low stabilities (*K*_d_ > mM) of [LEV ⊂ *pS*–**2**]^11−^ and [LEV ⊂ *pR*–**2**]^11−^. On the other side, protons from anionic dendrons (H_a‐c_, Figure 4c) were, for the most part, undergoing a relatively small perturbation of chemical shifts (Δ*δ* < 0.05 ppm). The observation corroborates the computed binding pose of [LEV ⊂ *pS*–**2**]^11−^ (Figure 4b) in which the included drug makes no noncovalent contacts with dendrons.

**Figure 6:**
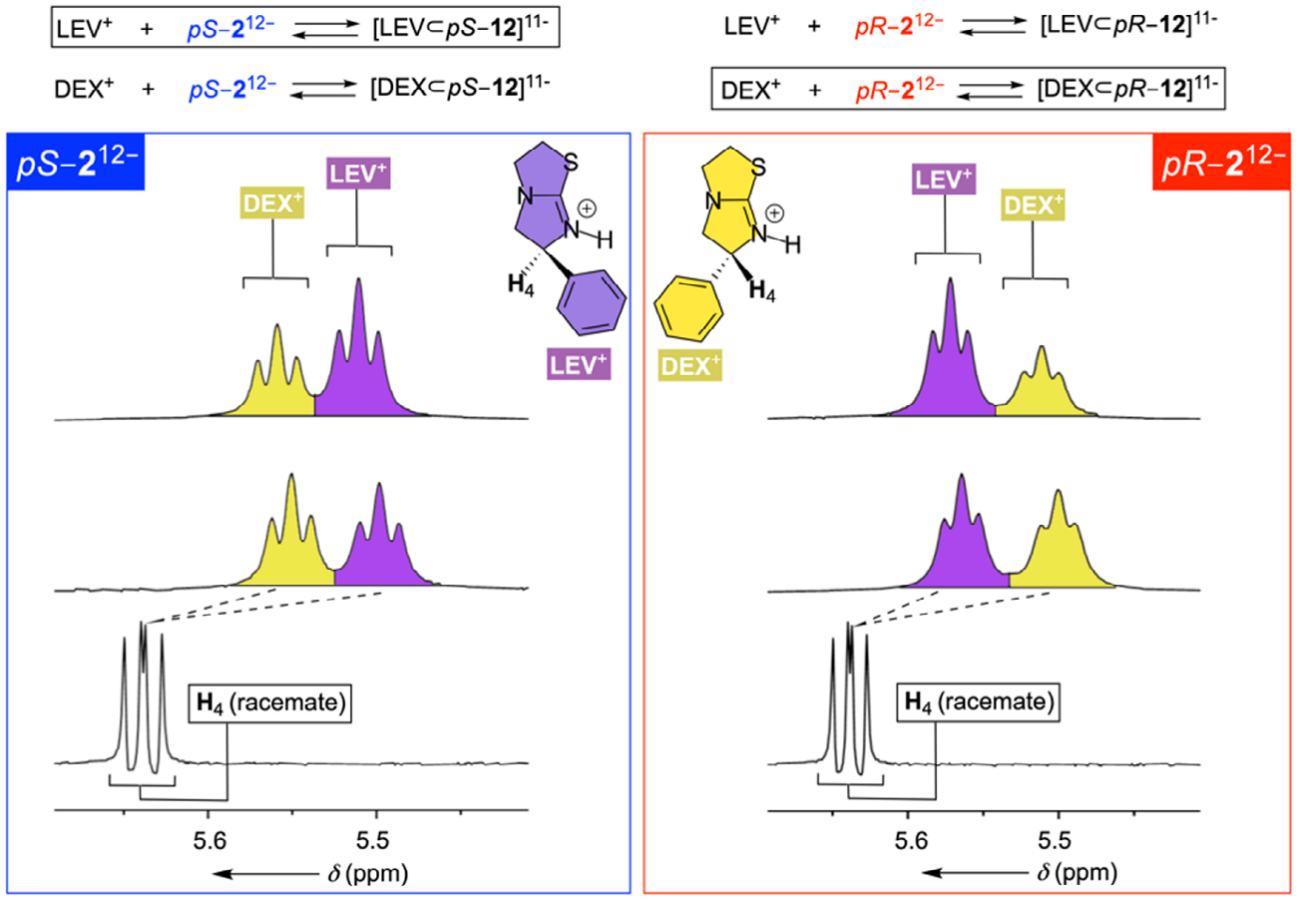
Left: A segment of ^1^H NMR spectrum (850 MHz, 298.0 K) of 0.5 mM tetramisole (LEX^+^ and DEX^+^, bottom), in 30 mM phosphate buffer at pH = 7.0, showing doublet of doublet signal from H_4_. In the presence of 0.5 mM *pS*–**12** ^12−^, the resonance from H_4_ splits into two with equal intensity (yellow and magenta). After spiking the sample with 0.5 mM LEV^+^, an increase in the intensity of more shielded (magenta) peak reveals its identity. Right: A segment of ^1^H NMR spectrum (850 MHz, 298.0 K) of 0.5 mM tetramisole (LEX^+^ and DEX^+^, bottom), in 30 mM phosphate buffer at pH = 7.0, showing doublet of doublet signal from H_4_. In the presence of 0.5 mM *pR*–**12** ^12−^, the resonance from H_4_ splits into two with equal intensity (yellow and magenta). After spiking the sample with additional 0.5 mM LEV^+^, an increase in the intensity of less shielded (magenta) peak reveals its identity.

While LEV^+^ assumes comparable binding poses inside diastereomeric *pS*/*pR*–**2** ^12−^, it has a slightly greater affinity for *pS*–**2** ^12−^ than *pR*–**2** ^12−^ (Figure 6, top left). Will enantiomeric DEX^+^ act in the opposite manner? That is to say, while LEV^+^ binds stronger to *pS*–**2** ^12−^, DEX^+^ could possess a greater affinity for *pR*–**2** ^12−^ (Figure 6, top right). Without enantiopure DEX^+^ available in laboratory, we went on to study the inclusion complexation of tetramisole (i.e., racemic mixture of LEV^+^ and DEX^+^) using *pS*–**2** ^12−^ (Figure 6, left) and *pR*–**2** ^12−^ (Figure 6, right); see also Figure S69. An addition of tetramisole (LEV^+^/DEX^+^) to *pS*–**2** ^12−^ resulted in the appearance of two sets of ^1^H NMR spectroscopic signals from the drug (yellow and magenta in Figure 6). Each set of resonances arose from LEV^+^ and DEX^+^ in a rapid exchange with diastereomeric [LEV ⊂ *pS*–**2**]^11−^ and [DEX ⊂ *pS*–**2**]^11−^, respectively.^[^ ^48^ ^]^ In this way, a doublet of doublet from H_4_ within the racemic drug (*δ* = 5.63 ppm; Figure 6, left) splits into a pair of doublet of doublets (appearing as triplets) in the presence of the host (*δ* = 5.55 ppm; Figure 6, left). The new resonances are magnetically shielded, well resolved, and with equivalent intensity thereby showing equal quantity of two enantiomers in the sample. After spiking the solution with LEV^+^, the more shielded magenta resonance (*δ* = 5.50 ppm; Figure 6, left) increased its intensity to be in line with LEV^+^ forming a more stable complex with *pS*–**2** ^12−^ than DEX^+^. On the contrary, when ^1^H NMR spectroscopic resolution of racemic LEV^+^/DEX^+^ was probed with *pR*–**2** ^12−^ the outcome was exactly reverse (Figure 6, right). In brief, cavitand *pR*–**2** ^12−^ favored DEX^+^ over LEV^+^, with H_4_ proton from the former showing a greater degree of shielding resulting from its more effective inclusion. The contrasting action of diastereomeric cavitands *pS*–**2** ^12−^ and *pR*–**2** ^12−^ complexing LEX^+^ and DEX^+^ goes along with the notion that their cylindrical cavity possessing planar chirality, and not dendrons, played the principal role in the recognition.

## Conclusion

Alkoxy substituents at two rims of pillar[6]arenes are geared in the same direction giving rise to two interconvertible and enantiomeric cavitands, each with *pS* or *pR* planar chirality. In this work, we introduced novel, general, and facile method for inhibiting such *pS* to *pR* interconversion while enabling cavitands’ solubility in water. By appending two chiral, peptidic, polyanionic, and sufficiently bulky dendrons to difunctionalized pillar[6]arene, we created dendritic *pS* and *pR* cavitands that, as diastereomers, were easily resolved by column chromatography. While soluble in water, *pS* and *pR* macrocycles were stable for weeks without interconversion. Diastereomeric pillar[6]arenes possess a unique binding pocket comprising a nonpolar and chiral cavity with two branched anionic nests at top and bottom. With an access to water soluble and chiral pillar[6]arenes for the first time, we decided to examine the potential of such novel hosts for stereoselective inclusion complexation of racemic and cationic drugs. Accordingly, ^1^H NMR spectroscopic measurements revealed stereoselective binding of cocaine adulterants, levamisole and dexamisole, with a distinct set of resonances from each drug within their racemic mixture. While polyanionic, dendritic, and chiral pillar[6]arenes can already be used as chiral shift reagents for determining enantiopurity of pharmaceuticals in water, we suggest that the results of our study also set the stage for a) examining chiral recognition, chemo sensing, and stereoselective sequestration of a broad range of small chiral drugs in aqueous media and b) creating dendritic pillar[6]arenes with other functional dendrons and fixed planar chirality for applications in the areas of chemo sensing, sequestration or delivery of drugs.

## Supporting Information

Supporting Information includes synthesis and characterization of molecules along with other spectroscopic and computational data.

## Conflict of Interests

The authors declare no conflict of interest.

## Supporting information



Supporting Information

## Data Availability

The data that support the findings of this study are available from the corresponding author upon reasonable request.
