# Supplementary material for: Dendritic Pillar[6]Arenes with Fixed Planar Chirality for Stereoselective Inclusions in Water: A Case of Facile Differentiation of Cocaine Adulterants, Levamisole and Dexamisole
Source: Angew Chem Int Ed Engl. 2025 Sep 1;64(43):e202514676. doi: 10.1002/anie.202514676 (PMC12535389; doi:10.1002/anie.202514676)
Supplement: Supplementary file 1 — Supporting Information [file ANIE-64-e202514676-s001.pdf]

Dendritic Pillar[6]Arenes with Fixed Planar Chirality for Stereoselective Inclusions in Water – A Case of Facile Differentiation of Cocaine Adulterants, Levamisole and Dexamisole

Nitesh Kumar,[a][d] Pratik Karmakar,[b][d] Matthew Politeski,[a] Alexandar R. Hansen,[c] Carson E. Ward,[a] Christopher Mortensen,[a] Christopher M. Hadad,[a] Kornkanya Pratumyot,[b] and Jovica D. Badjić*[a]

[a] N. Kumar, M. Politeski, C. E. Ward, C. Mortensen, Prof. C. M. Hadad, Prof. J. D. Badjić
Department of Chemistry and Biochemistry
The Ohio State University
100 West 18th Avenue, Columbus, OH 43210 (USA)
E-mail: badjic.1@osu.edu

[b] P. Karmakar, Asst. Prof. K. Pratumyot
Supramolecular Chemistry Research Unit, Department of Chemistry, Faculty of Science
King Mongkut's University of Technology Thonburi
126 Pracha Uthit Road, Bang Mod, Thung Khru, Bangkok 10140 (Thailand)

[c] Dr. A. L. Hansen
Campus Chemical Instrument Center
The Ohio State University
100 West 18th Avenue, Columbus, OH 43210 (USA)

[d] N. Kumar and P. Karmakar contributed equally to this work.

Supporting Information

Table of Contents

General Information 3

Synthesis of Pillar[6]Arenes 7-10 4

NMR Spectra of Pillar[6]Arenes 7-10 8

Preparation of Dendrons 4-6 17

Characterization of Dendrons 4-6 21

Synthesis and Resolution of Dendritic Pillar[6]Arenes *pS*/*pR*-11/12/13 30

Spectroscopic Characterizations of Dendritic Pillar[6]Arenes *pS*/*pR*-11/12/13 33

Diffusion (DOSY) NMR Analysis of Pillar[6]arenes 467

Variable temperature ^{1}H NMR Spectroscopic Studies of Pillar[6]Arenes 50

Synthesis and Characterization of Dendritic Pillar[6]Arenes *pS*/*pR*-11^{8-} and *pS*/*pR*-11^{8-} 52

Inclusion Complexation Studies of Levamisole (LEV^{+}) and Dexamisole (DEX^{+}) with Dendritic Pillar[6]Arenes *pS*-2^{12-} and *pR*-2^{12-} 68

Computational Studies 75

General Information

All chemicals were purchased from commercial sources and used as received unless stated otherwise. All solvents were dried before use according to standard literature procedures. Chromatographic purifications were performed with silica gel 60 (SiO_2, 40–75 µm, 200 × 400 mesh). Thin-layer chromatography was performed on a silica gel plate w/UV254 (200 µm). Chromatograms were visualized by UV light or stained with ninhydrin, iodine (I_2), or ceric ammonium molybdate (CAM). All NMR experiments were performed with Bruker 400, 600, and 850 MHz spectrometers. Deuterated solvents CD_2Cl_2, and $(CD_3)_2SO$, and D_2O were acquired from Cambridge Isotope Laboratories. NMR data was processed and analyzed using Bruker software (Topspin 4.2, Dynamics center 2.8, MestReNova 15.0.0). High-resolution mass data (HRMS) were obtained using electrospray ionization (ESI) techniques (Bruker ESI TOF and Thermo-fisher ESI orbitrap instruments). Acidity (pH) measurements were completed with an HI 2210 pH meter.

Synthesis of Pillar[6]Arenes 7-10

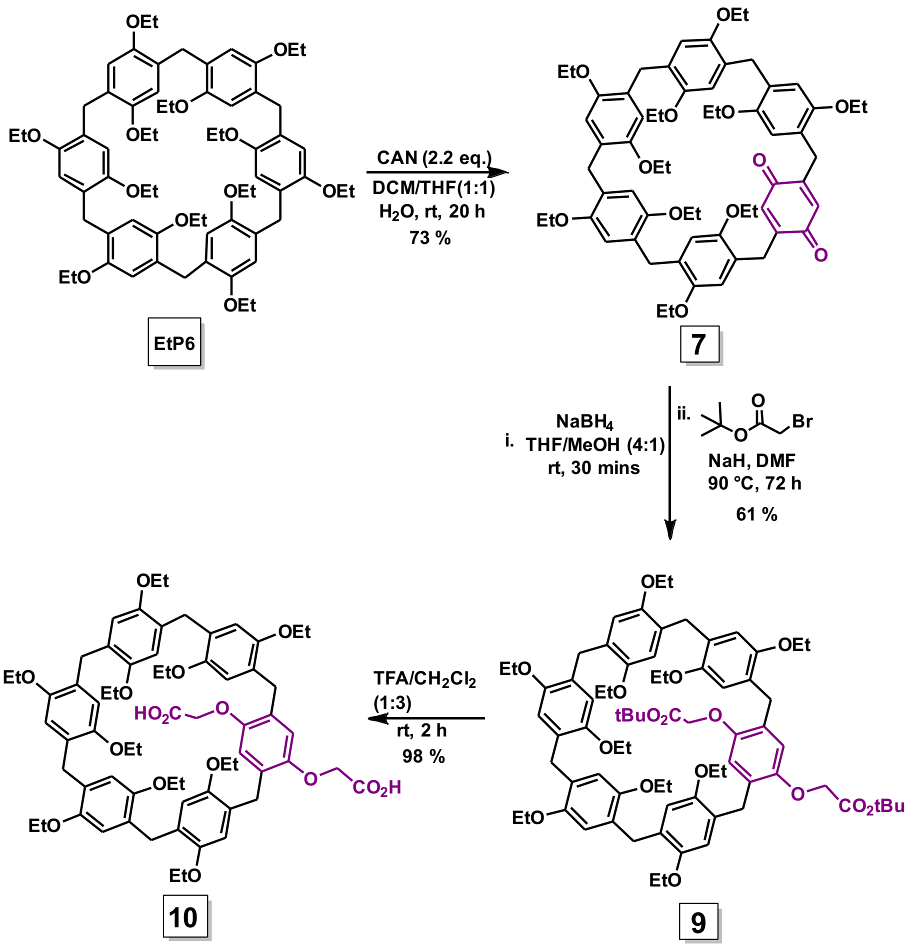

Scheme S1. A synthetic scheme describing the preparation of pillar[6]arene **10**. Note that 2,5-*bis*(ethoxy)pillar[6]arene (EtP6) was obtained by following literature procedure (Wilson, C.R., Chen, E.F., Puckett, A.O. and Hof, F., *Org. Synth.* **2022**, *99*, 125-138).

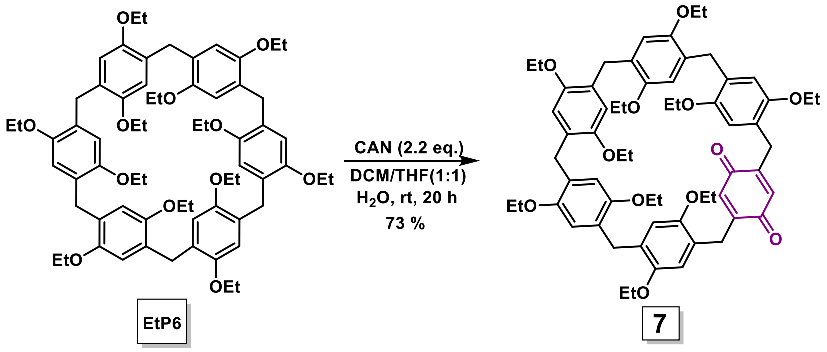

EtP6 7

Compound 7. This compound was obtained by a modified literature procedure (C. Han, L. Gao, G. Yu, Z. Zhang, S. Dong, F. Huang, Eur. *J. Org. Chem.* **2013**, 2529-2532). Cerium (IV) ammonium nitrate (1.69 g, 3.09 mmol) dissolved in 20 mL of water was added dropwise to a solution of 2,5-*bis*(ethoxy)pillar[6]arene (1.5 g, 1.40 mol) in 160 mL of CH_2Cl_2/THF (1:1). The reaction mixture was stirred at room temperature for 20 h after which 50 mL of CH_2Cl_2 was added. After the removal of water by separatory funnel, the organic layer was dried with sodium sulfate and evaporated under reduced pressure. Crude product was purified by column chromatography (SiO_2, ethyl acetate: hexane = 1:9) to give 1.04 g (73 %) of **7** as a bright red solid. ^{1}H NMR (400 MHz, $CDCl_3$) δ (ppm) = 6.75 (s, 2H), 6.70 (s, 2H), 6.69 (s, 2H), 6.68 (s, 2H), 6.62 (s, 2H), 6.44 (s, 2H), 3.94 - 3.74 (m, 28H), 3.57 (s, 4H), 1.37 – 1.21 (m, 30H). ^{13}C NMR (101 MHz, $CDCl_3$) δ (ppm) = 188.32 (s), 150.66 (s), 150.54 (s), 146.72 (s), 133.67 (s), 129.49 (s), 128.37 (s), 128.10 (s), 127.40 (s), 122.80 (s), 115.64 (s), 115.38 (s), 115.27 (s), 115.07 (s), 114.64 (s), 64.33 (s), 64.24 (s), 64.20 (s), 64.07 (s), 63.67 (s), 63.67 (s), 31.05 (s), 30.63 (s), 30.39 (s), 26.98 (s), 15.21 (s), 15.16 (s), 14.99 (s). HRMS (ESI): *m*/*z* calculated for $C_{62}H_{78}O_{12}N^+$ $[M + NH_4]^+$ = 1028.5524, found 1028.5728.

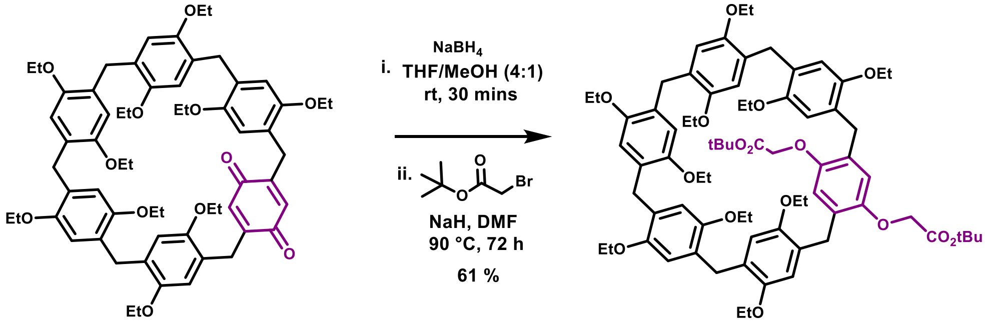

7 9

Compound 9. To a solution of compound **7** (600 mg, 0.59 mmol) in a mixture of THF (20 mL) and CH_3OH (5 mL) was added $NaBH_4$ (100 mg, 2.64 mmol). After stirring at room temperature for 20 minutes, CH_2Cl_2 (100 mL) was added, and the mixture was poured into aqueous 0.1 M HCl. The organic layer was separated by separatory funnel, dried over anhydrous Na_2SO_4 and evaporated to give 590 mg of compound **8** (Scheme 1B,

main text) as a pale-yellow solid. Compound **8** was used without further purification. ^{1}H NMR (400 MHz, CDCl3) δ 6.85 (s, 1H), 6.75 (s, 3H), 6.68 (dd, J = 6.2, 2.4 Hz, 6H), 6.62 – 6.59 (m, 3H), 6.43 (s, 1H), 3.87-3.78 (m, 32H), 1.38 – 1.29 (m, 30H), HRMS (ESI): *m/z* calculated for $C_{62}H_{76}O_{12}$ $[M + NH_4]^+$ = 1030.5675, found 1030.5887. Compound **8** (590 g, 0.58 mmol) was dissolved in 25 mL of anhydrous DMF under an atmosphere of nitrogen, followed by the addition of 1.36 g of *tert*-butyl 2-bromoacetate (7.0 mmol). Next, 280 mg of 60% NaH (7.0 mmol) was added slowly. After stirring the mixture at 90 °C for 72 h, methanol (5 mL) was added to quench the unreacted sodium hydride. Solvent was evaporated under reduced pressure, 50 mL of CH_2Cl_2 was added and the solution was treated with water (25 mL) and saturated NH_4Cl solution (20 mL) using separatory funnel. The organic layer was dried with anhydrous Na_2SO_4 and the solvent removed under reduced pressure to give crude **9**. After purification by column chromatography (SiO_2, ethyl acetate: hexane = 1:9) compound **9** (440 mg, 61 %) was obtained as a yellow solid. ^{1}H NMR (400 MHz, $CDCl_3$) δ (ppm) = 6.91 (s, 2H), 6.77 (s, 2H), 6.72 (s, 2H), 6.69 (s, 2H), 6.68 (s, 2H), 6.67 (s, 2H), 4.35 (s, 4H), 3.89 – 3.81 (m, 32H), 1.35 (s, 18H), 1.34 – 1.30 (m, 30H). ^{13}C NMR (101 MHz, $CDCl_3$) δ (ppm) = 168.36 (s), 150.60 (s), 150.47 (s), 150.42 (s), 150.06 (s), 128.29 (s), 128.01 (s), 127.98 (s), 127.77 (s), 127.35 (s), 115.61 (s), 115.57 (s), 115.35 (s), 115.12 (s), 115.00 (s), 81.74 (s), 66.58 (s), 64.12 (s), 64.10 (s), 64.06 (s), 63.98 (s), 63.86 (s), 31.09 (s), 31.06 (s), 30.73 (s), 27.87 (s), 15.24 (s), 15.21 (s), 15.17 (s). HRMS (ESI): *m/z* calculated for $C_{74}H_{100}O_{16}N^+$ $[M + NH_4]^+$ = 1258.7042, found 1258.7002.

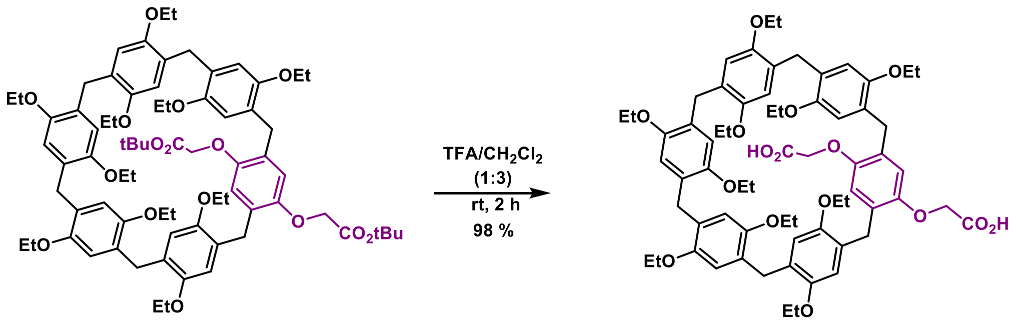

9

10

Compound 10. Pillar[6]arene **9** (400 mg, 0.32 mmol) was dissolved in 40 mL of CF_3COOH:CH_2Cl_2 = 1:3 and, under an atmosphere of nitrogen, stirred at room temperature for 2 h. The solvent was removed under reduced pressure and 50 mL of water was added followed by a dropwise addition of 1 M NaOH until pH became close to 10. The clear solution was then transferred to a centrifuge tube and **10** was precipitated by a slow addition of 4 M HCl until pH was close to 3. Centrifugation of the suspension followed by washing the solid 3-4 times with distilled water and lyophilization gave 355 mg of **10** (98 %) as an off-white solid. ^{1}H NMR (400 MHz, DMSO-d_6) δ (ppm) = 6.84 (s, 2H), 6.65 (d, J = 1.9 Hz, 6H), 6.60 (d, J = 1.6 Hz, 4H), 4.40 (s, 4H), 3.83 – 3.73 (m, 24H),

3.66 (d, J = 4.9 Hz, 8H), 1.22 (m, 30H). ^{13}C NMR (101 MHz, DMSO-d^6) δ (ppm) =170.22 (s), 149.79 (s), 149.75 (s), 149.66 (s), 149.52 (s), 127.30 (s), 127.01 (s), 126.97 (s), 126.89 (s), 126.62 (s), 114.94 (s), 114.86 (s), 114.78 (s), 114.67 (s), 114.43 (s), 65.47 (s), 63.40 (s), 63.30 (s), 30.73 (s), 14.84 (s). HRMS (ESI): *m*/*z* calculated for $C_{66}H_{84}O_{16}N^+$ $[M + NH_4]^+$ = 1146.5790 (calculated), found 1146.5772.

NMR Spectra of Pillar[6]Arenes 7-10

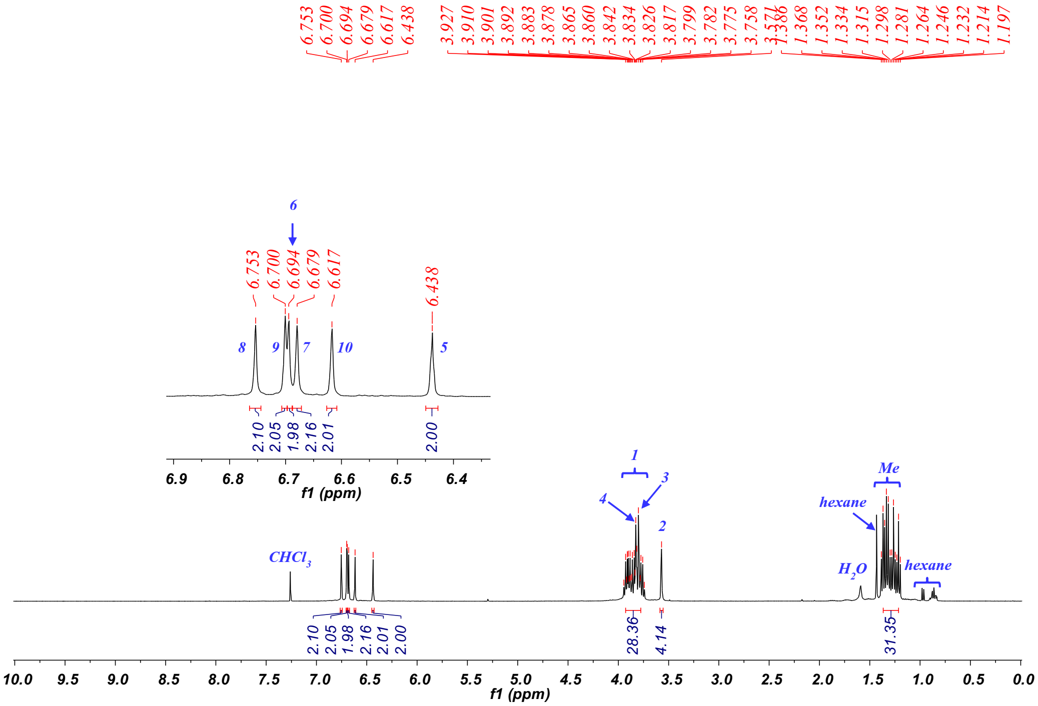

Figure S1. ^{1}H NMR spectrum (400 MHz, 298 K) of compound **7** in $CDCl_3$.

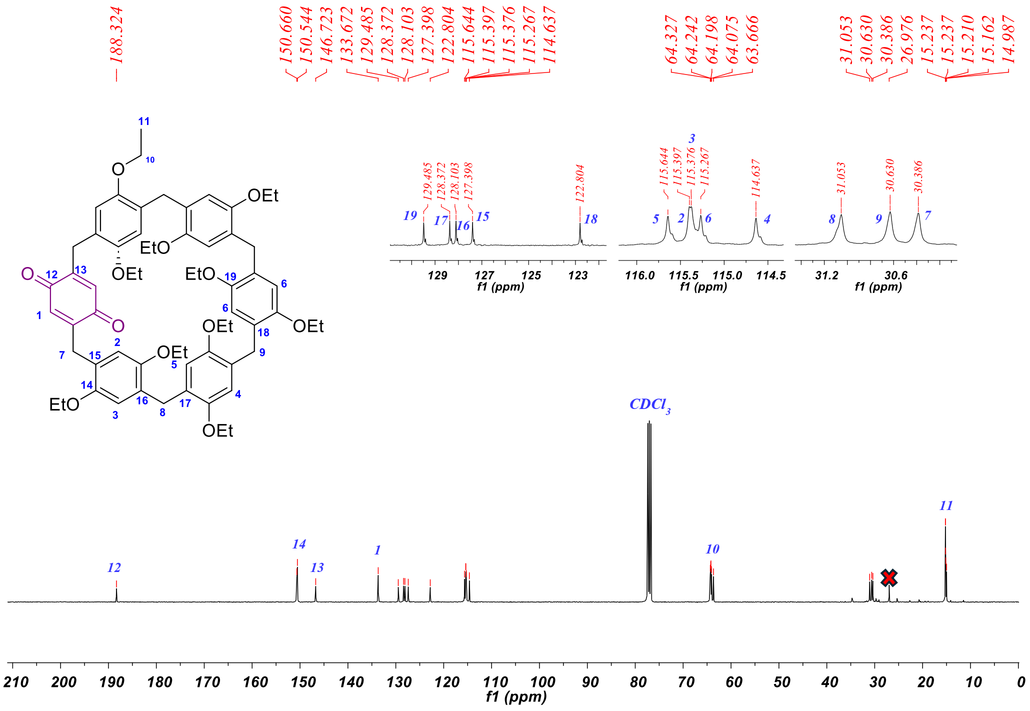

Figure S2. ^{13}C NMR spectrum (100 MHz, 298 K) of compound **7** in $CDCl_3$.

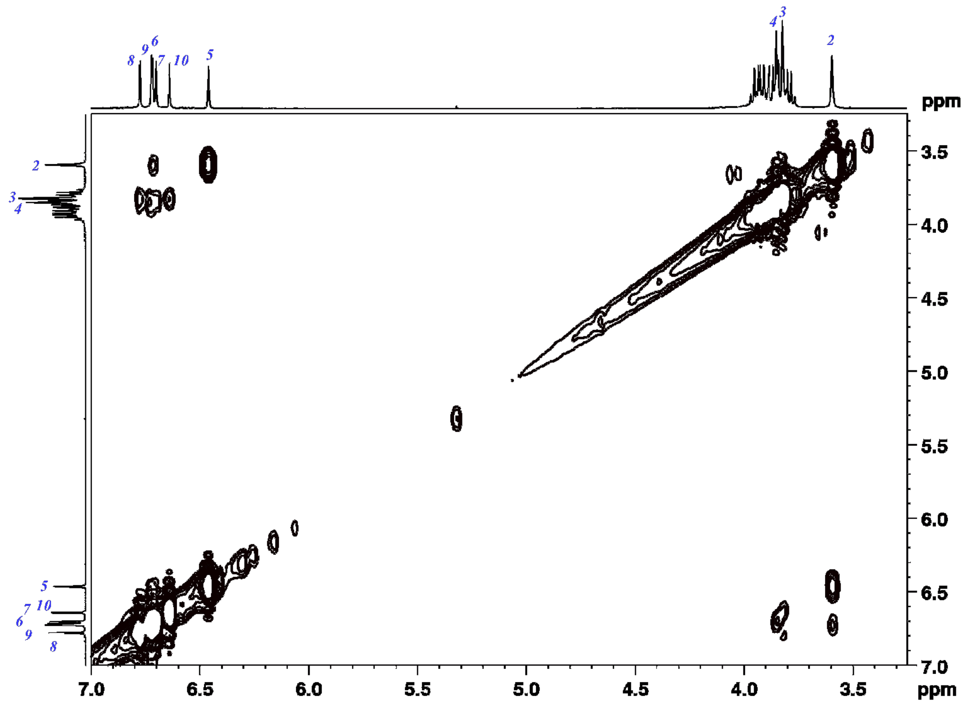

Figure S3. A segment of ^{1}H-^{1}H COSY NMR (400 MHz, 298 K) of compound **7** in $CDCl_3$.

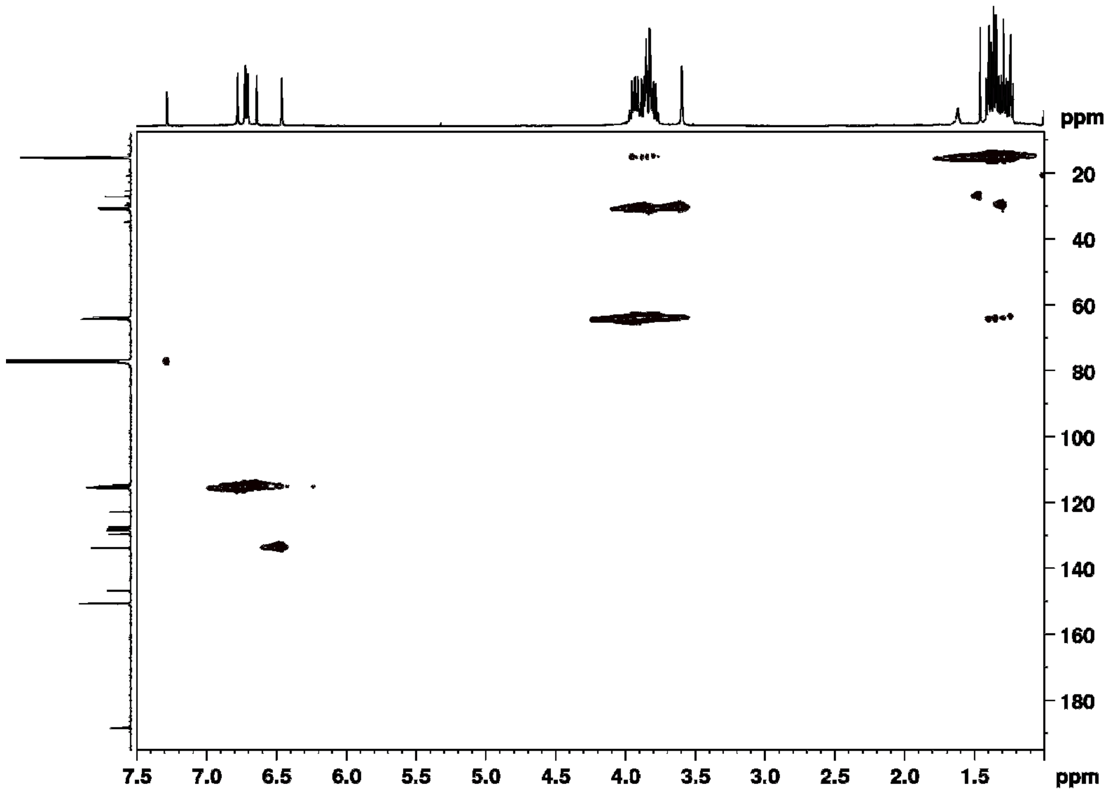

Figure S4. ^{1}H-^{13}C HSQC NMR spectrum (400 MHz, 298 K) of compound **7** in $CDCl_3$.

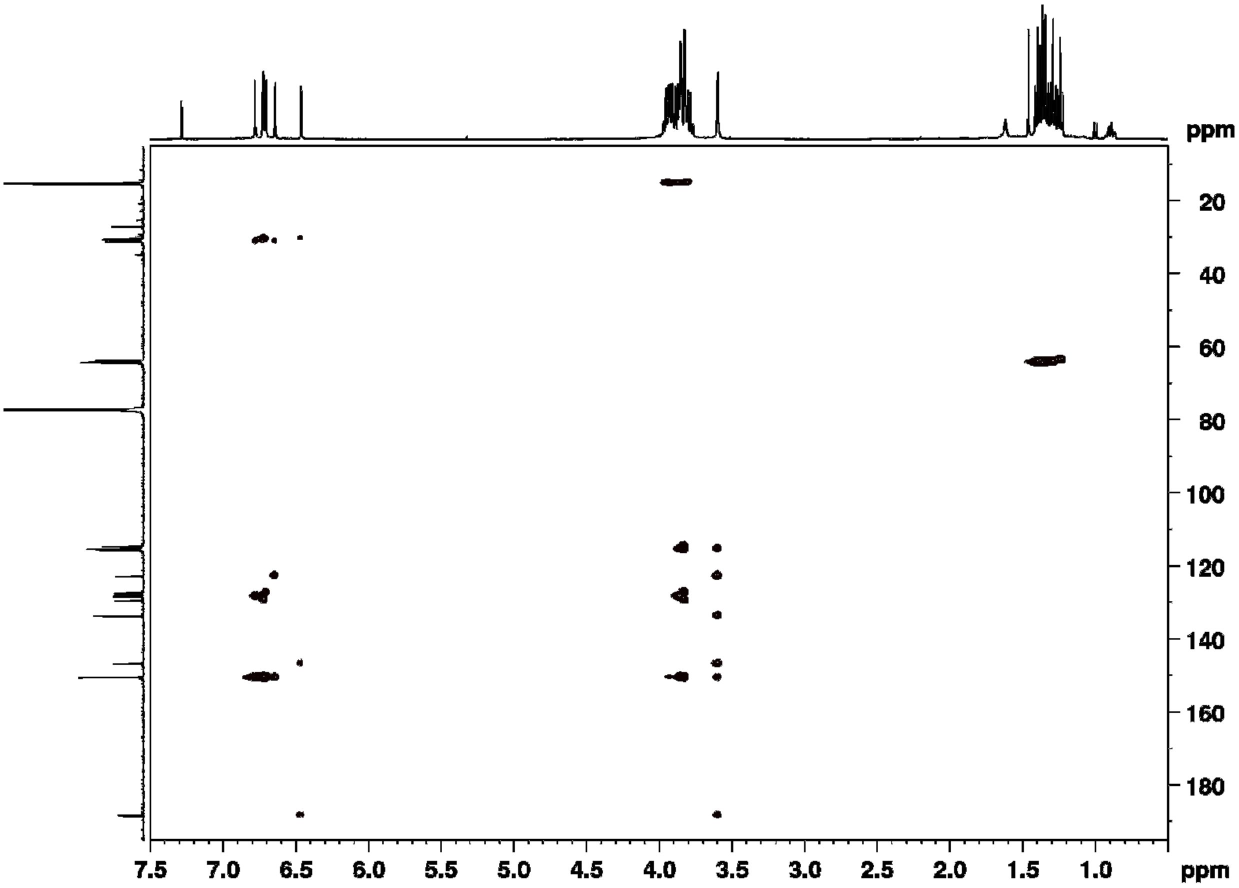

Figure S5. ^{1}H-^{13}C HMBC NMR spectrum (400 MHz, 298 K) of compound **7** in $CDCl_3$.

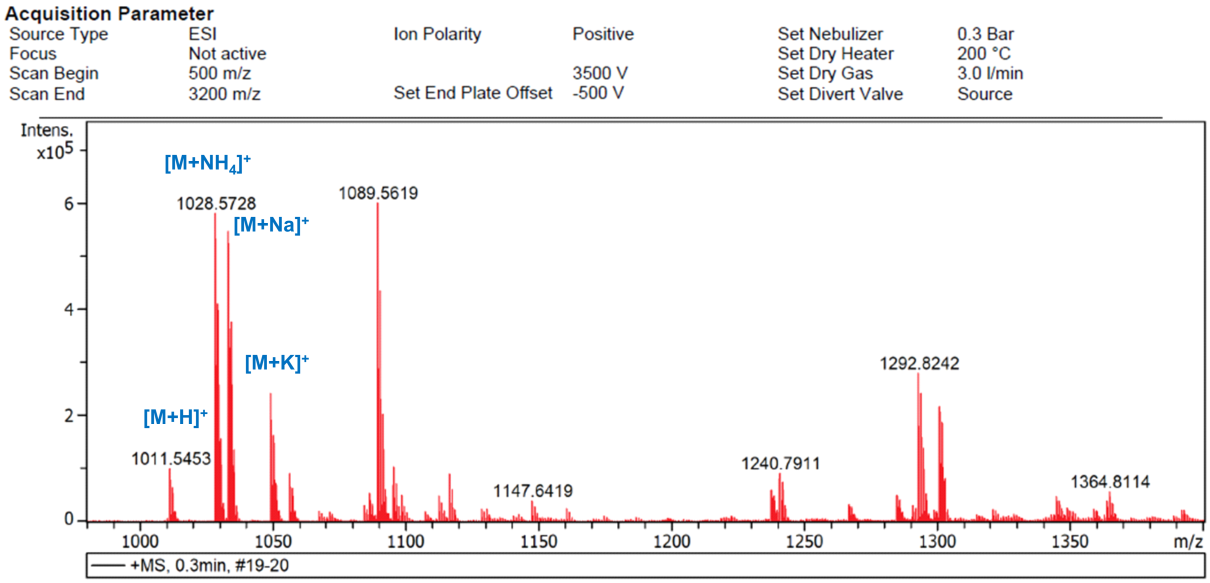

Figure S6. HRMS (ESI-TOF) of compound **7** using positive ionization mode, suggesting the formation of $[\mathbf{7}+NH_4]^+$ and $[\mathbf{7}+Na]^+$ cations.

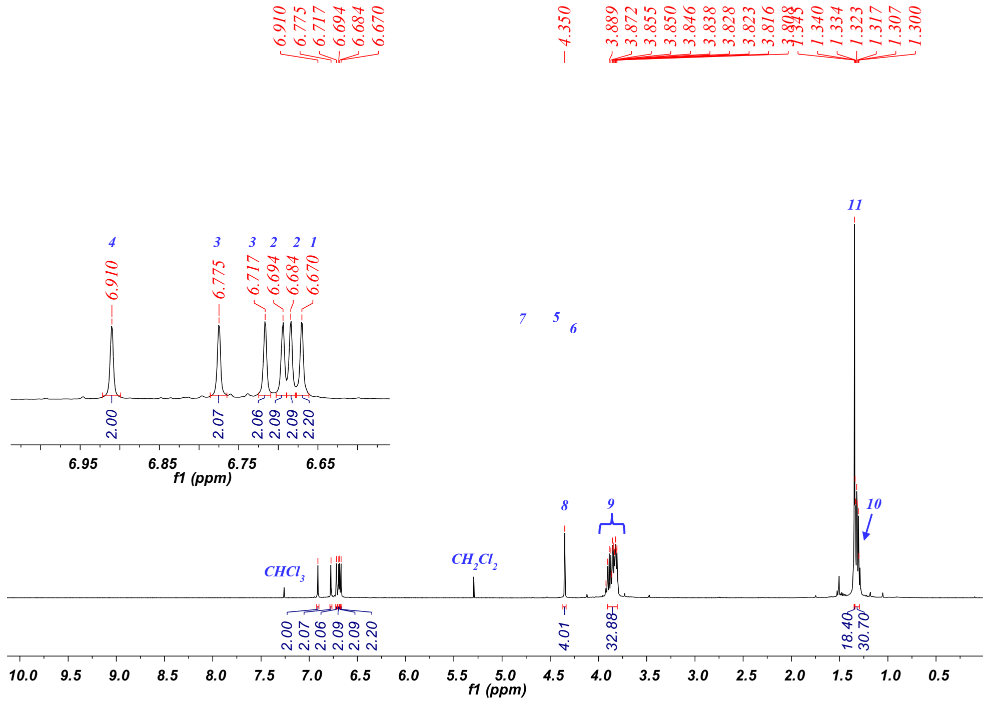

Figure S7. ^{1}H NMR spectrum (400 MHz, 298 K) of compound **9** in $CDCl_3$.

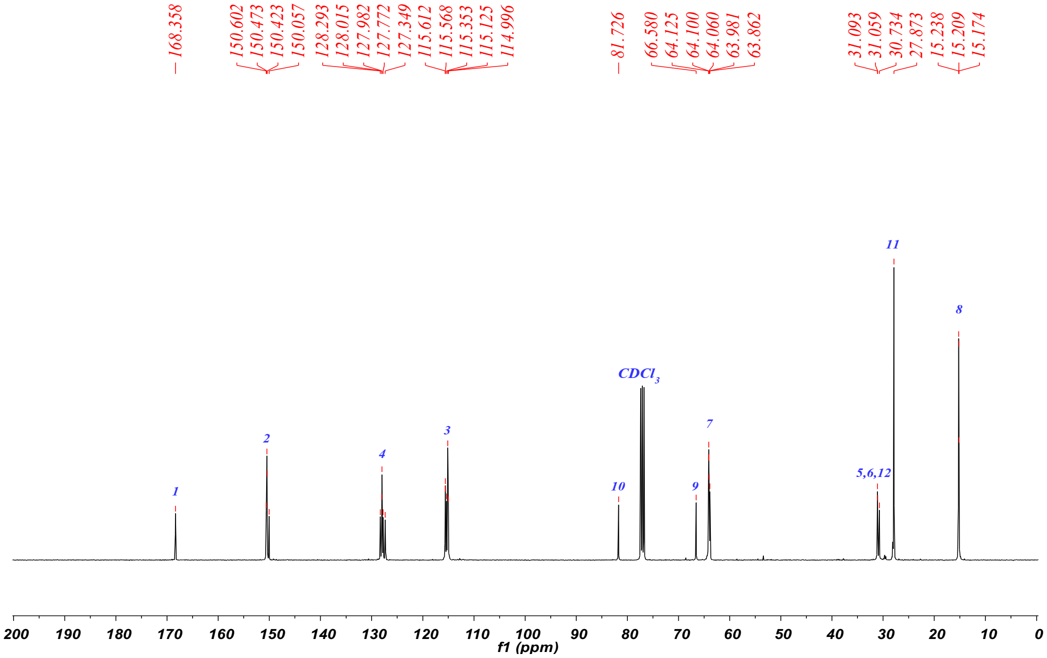

Figure S8. ^{13}C NMR spectrum (100 MHz, 298 K) of compound **9** in $CDCl_3$.

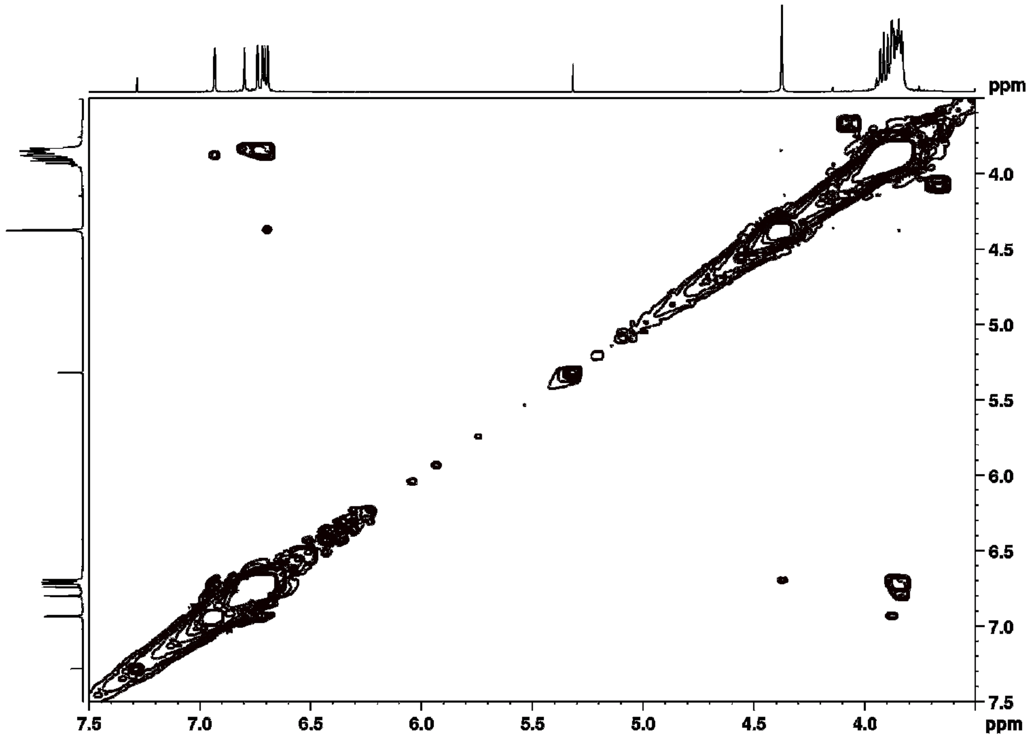

Figure S9. ^{1}H-^{1}H COSY NMR spectrum (400 MHz, 298 K) of compound **9** in $CDCl_3$.

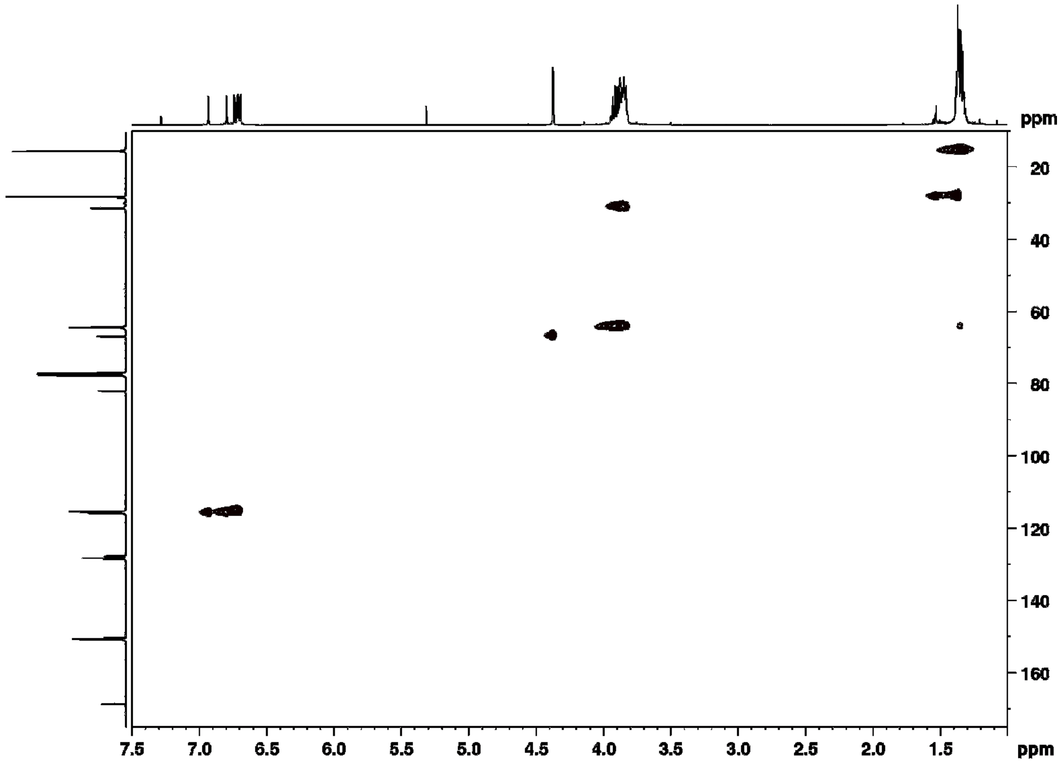

Figure S10. ^{1}H-^{13}C HSQC NMR spectrum (400 MHz, 298 K) of compound **9** in $CDCl_3$.

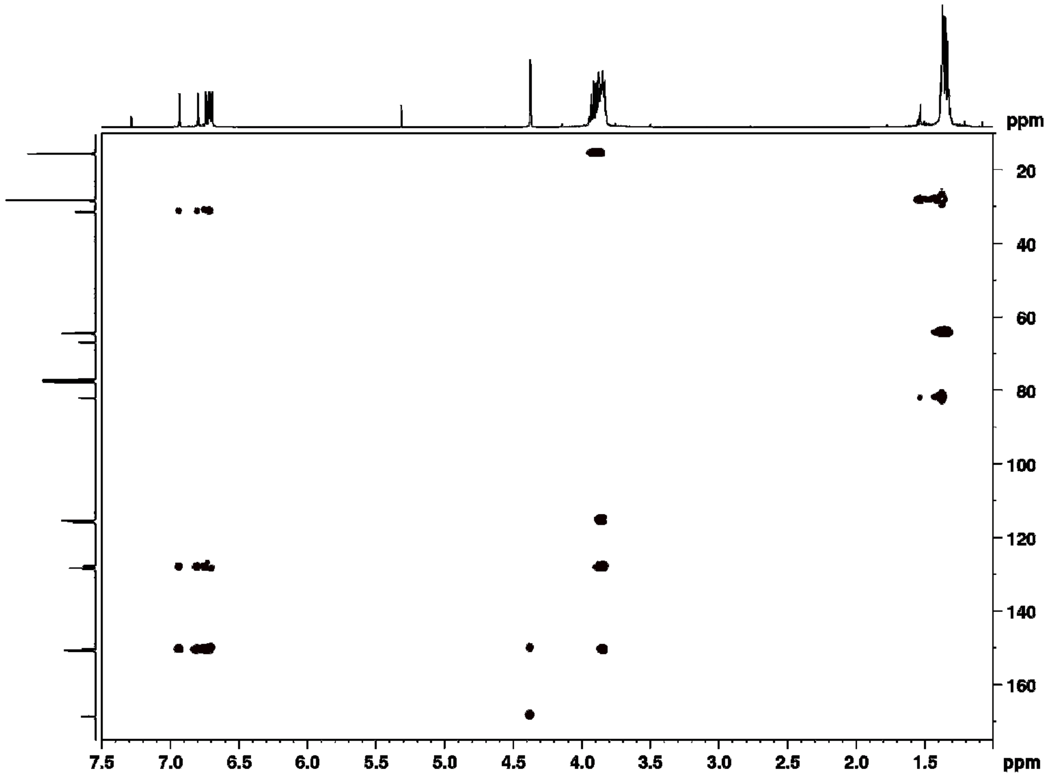

Figure S11. ^{1}H-^{13}C HMBC NMR spectrum (400 MHz, 298 K) of compound **9** in $CDCl_3$.

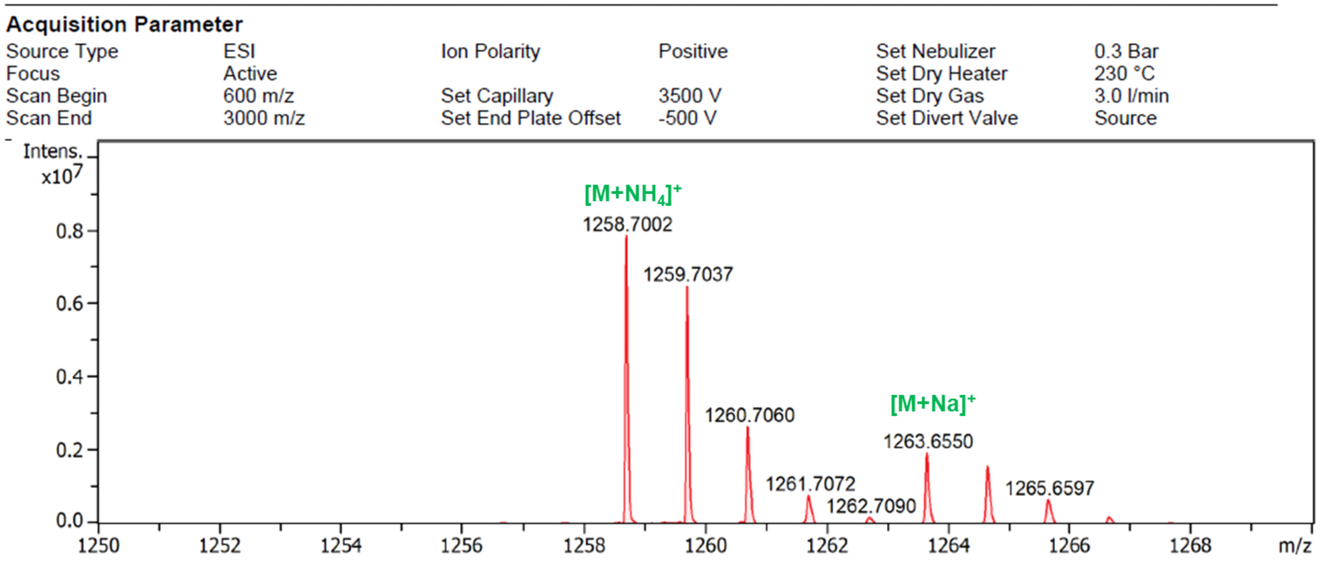

Figure S12. HRMS (ESI-TOF) of compound **9** using positive ionization mode, suggesting the formation of [**9**+NH_4]$^+$ and [**9**+Na]$^+$ cations.

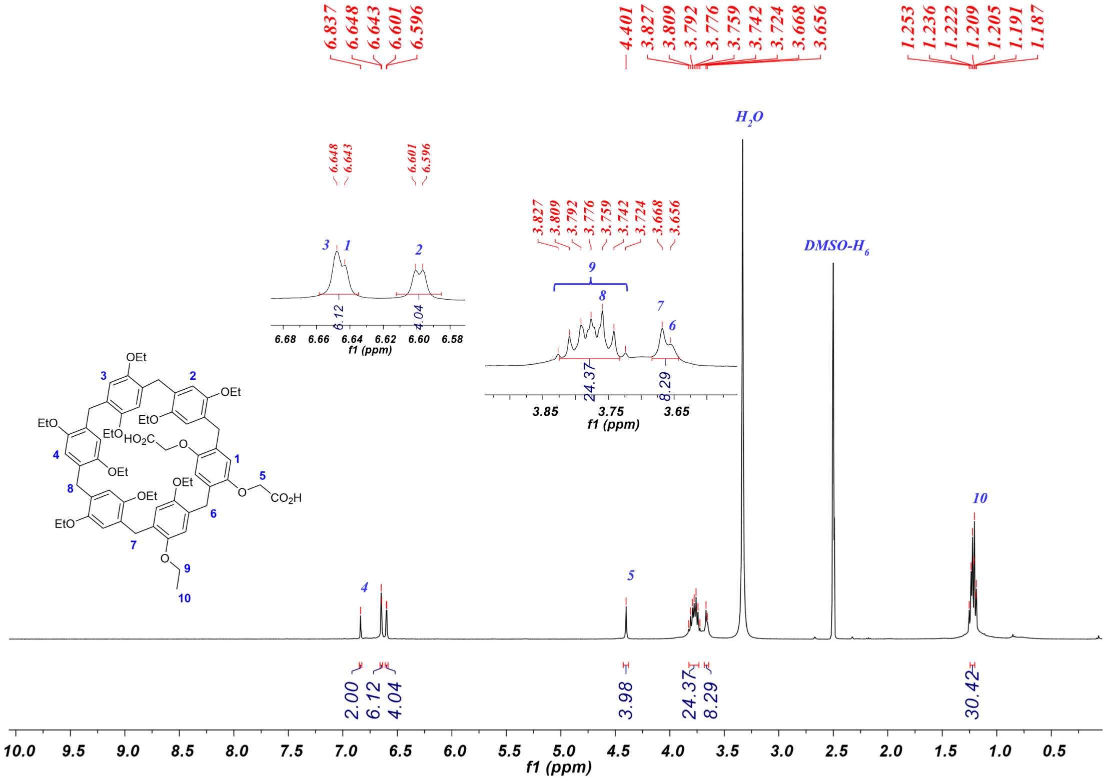

Figure S13. ^{1}H NMR spectrum (400 MHz, 298 K) of compound **10** in DMSO-d6.

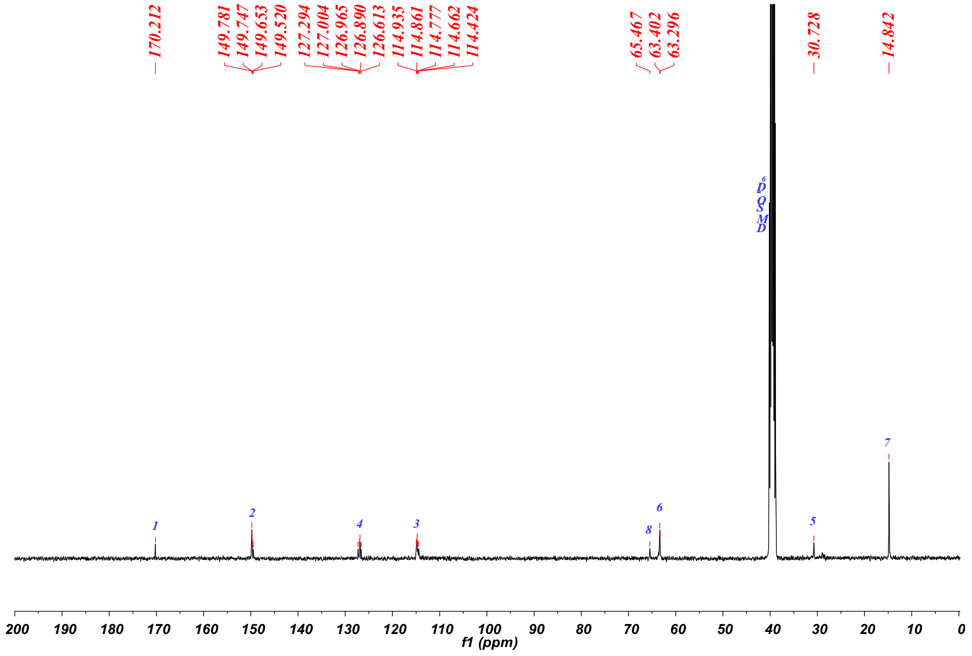

Figure S14. ^{13}C NMR spectrum (100 MHz, 298 K) of compound **10** in DMSO-d6.

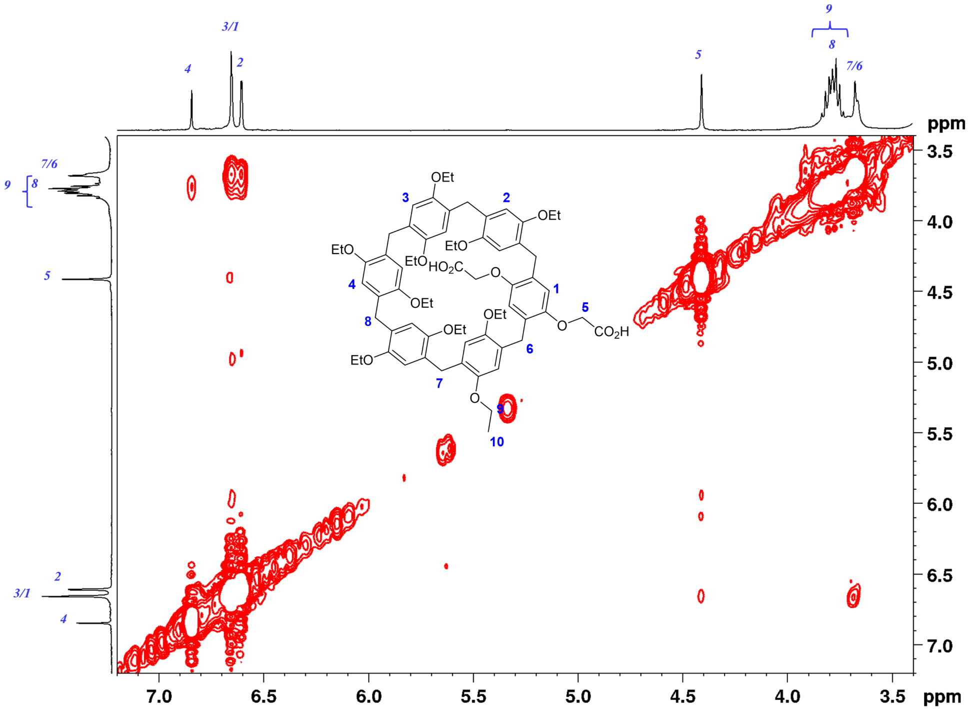

Figure S15. ^{1}H-^{1}H COSY NMR spectrum (400 MHz, 298 K) of compound **10** in DMSO-d$_6$.

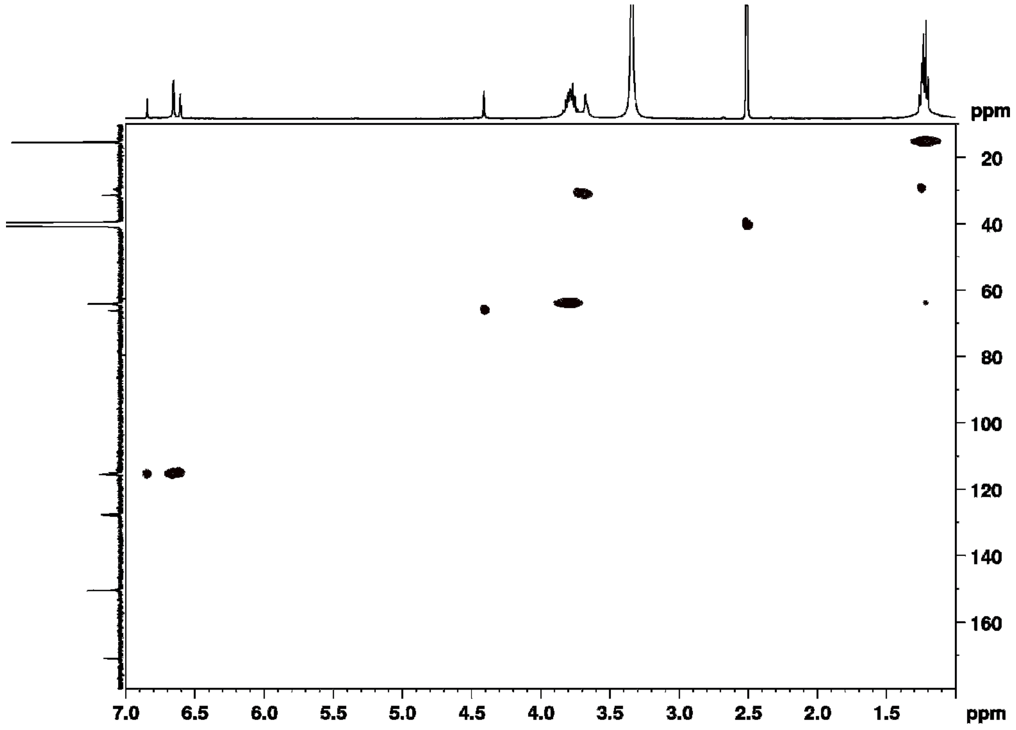

Figure S16. ^{1}H-^{13}C HSQC NMR spectrum (400 MHz, 298 K) of compound **10** in DMSO-d$_6$.

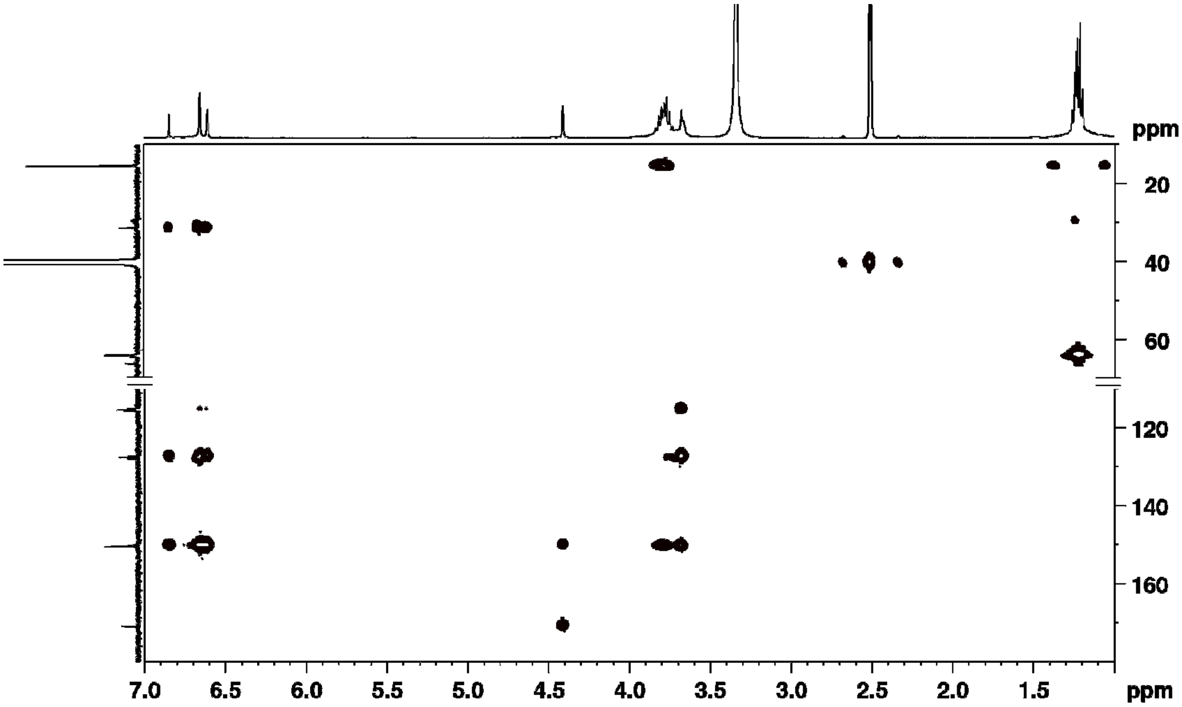

Figure S17. ^{1}H-^{13}C HMBC NMR spectrum (400 MHz, 298 K) of compound **10** in DMSO-d$_6$.

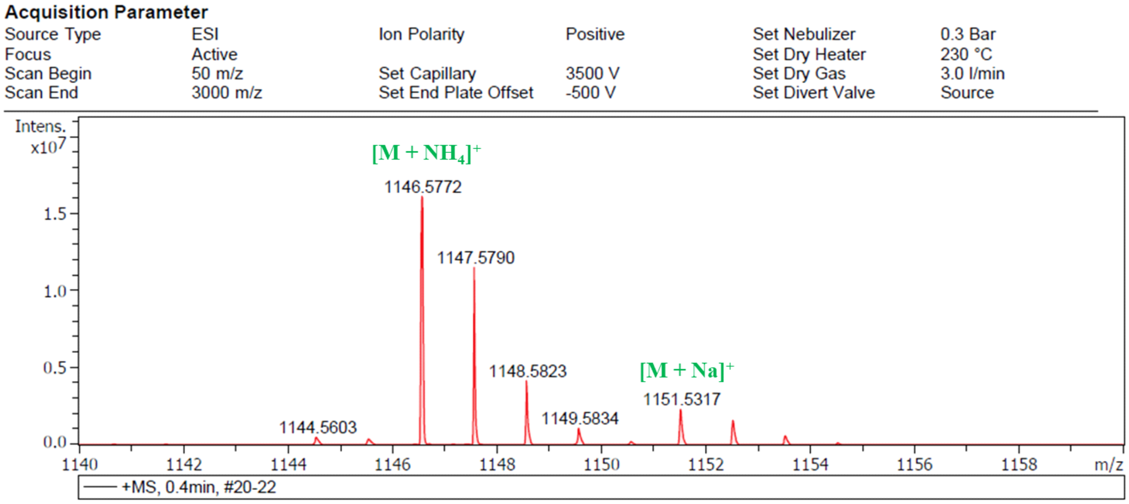

Figure S18: HRMS (ESI-TOF) of **10** using positive ionization mode, suggesting the formation of [**10**+NH_4]$^+$ and [**10**+Na]$^+$ cations.

Preparation of Dendrons 4-6

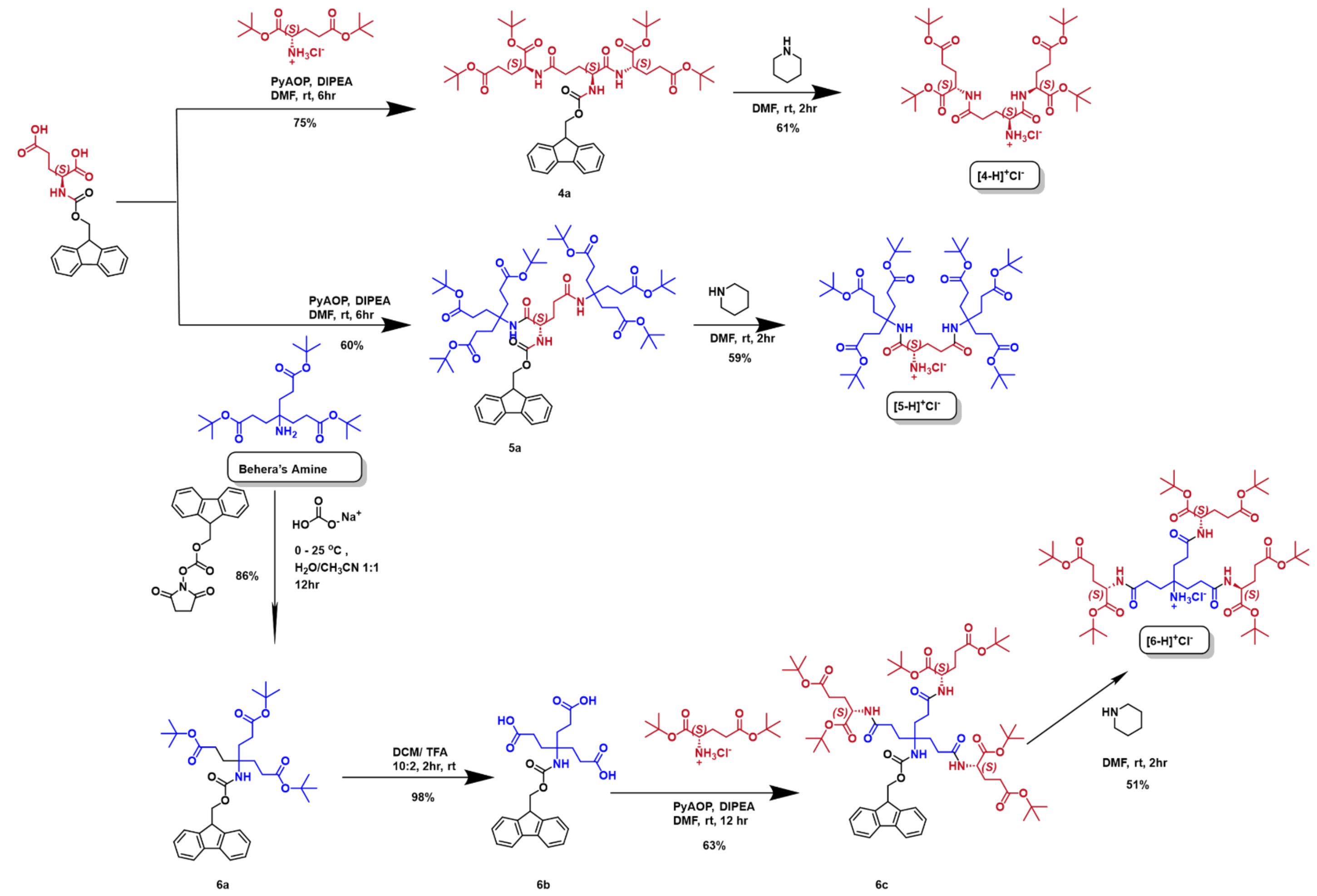

Scheme S2. A synthetic scheme describing the preparation of dendrons **4**, **5**, and **6**.

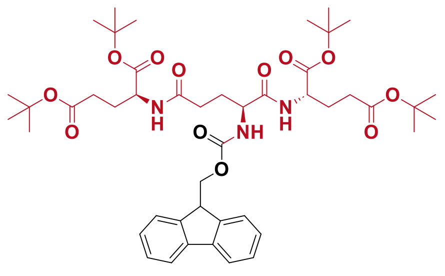

4a

Compound 4a. Compound **4a** was synthesized using a procedure reported by us (N. Kumar, T. J. Finnegan, S. Taneja, D. C. Rostam, A. L. Hansen, C. E. Ward, S. Ucar, J. R. Parquette, J. D. Badjic, *Angew. Chem., Int. Ed.* **2024**, *64*, e202420574).^{1}H NMR (400 MHz, DMSO) δ (ppm): 8.18 (dt, J = 7.6 Hz, 1H), 8.08 (dt, J = 7.6 Hz, 1H), 7.90 (dt, J = 7.8, 0.9 Hz, 2H), 7.74 (dd, J = 7.4, 5.3 Hz, 2H), 7.53 (d, J = 8.2 Hz, 1H), 7.42 (td, J = 7.5, 1.2 Hz, 2H), 7.33 (td, J = 7.5, 1.2 Hz, 2H), 4.30 – 4.17 (m, 3H), 4.18 – 4.06 (m, 2H), 4.08 – 3.98 (m, 1H), 2.32 – 2.18 (m, 6H), 1.98 – 1.84 (m, 3H), 1.79 – 1.70 (m, 4H), 1.43 – 1.37 (m, 36H). HRMS (ESI-MS): *m/z* calculated for $C_{46}H_{66}N_3O_{12}$: 852.4641 [M+H]+; found: 852.4659.

Dendron [4-H]$^+$Cl$^-$. Compound **4a** (1.0 g, 1.17 mmol) was dissolved in 60 mL of dry dichloromethane and cooled to 0°C. Piperidine (12 mL) was slowly added, and the reaction mixture was stirred for 2 h. The reaction mixture was diluted with 60 mL of dichloromethane, washed with water (2 x 25 mL) and brine (25 mL). The organic layer was dried using anhydrous sodium sulfate and concentrated under reduced pressure. Crude **4** was purified using column chromatography (SiO_2, from hexane: ethyl acetate = 1:1 to by ethyl acetate: triethylamine = 100:1) to give dendron **4** as an oil; the chromatographic elution of **4** was monitored with Ninhydrin stain. The oil was redissolved in dichloromethane and washed with 1M HCl. The organic layer was dried with sodium sulfate and the solvent was evaporated under reduced pressure to give dendron **[4-H]$^+$Cl$^-$** (452 mg, 61 %) as a white solid. ^{1}H NMR (400 MHz, DMSO) δ (ppm) = 8.85 (d, J = 7.3 Hz, 1H), 8.36 (s, 3H), 8.32 (d, J = 7.5 Hz, 1H), 4.21 (ddd, J = 9.0, 7.3, 5.1 Hz, 1H), 4.12 (ddd, J = 9.3, 7.5, 5.2 Hz, 1H), 3.85 (t, J = 6.5 Hz, 1H), 2.40 – 2.29 (m, 5H), 2.29 – 2.22 (m, 2H), 2.03 – 1.85 (m, 4H), 1.85 – 1.68 (m, 2H), 1.41 (s, 9H), 1.40 (s, 9H), 1.40 (s, 9H), 1.39 (s, 9H). ^{13}C NMR (101 MHz, DMSO) δ (ppm) = 171.8, 171.8, 171.8, 171.5, 170.7, 169.0, 81.6, 81.1, 80.4, 80.3, 52.5, 52.3, 52.1, 31.6, 31.4, 31.1, 28.2, 28.2, 28.1, 28.0, 27.7, 26.7, 26.6. HRMS (ESI-MS): *m/z* calculated for $C_{31}H_{56}N_3O_{10}$: 630.3960 [M+H]+; found: 630.4040.

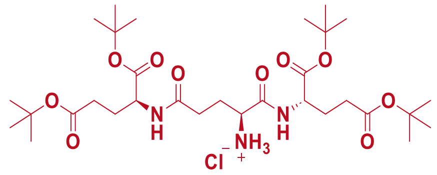

[4-H]$^+$Cl$^-$

Behera's Amine. Behera's amine was synthesized using a previously described procedure (G. R. Newkome, R. K. Behera, C. N. Moorefield, and G. R. Baker *J. Org. Chem.* **1991**, 56, 7162). ^{1}H NMR (400 MHz, DMSO) δ (ppm) = 2.21 – 2.06 (m, 6H), 1.47 – 1.33 (m, 33H). ^{13}C NMR (101 MHz, DMSO) δ (ppm) = 173.1, 79.7, 52.3, 34.3, 29.9, 28.2.

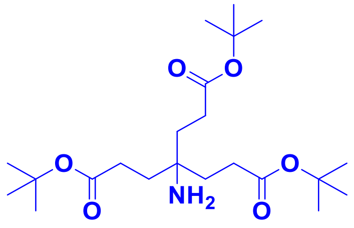

Behera's Amine

Compound 5a. Fmoc-protected (*S*)-glutamic acid (850 mg, 2.3 mmol), Behera's amine (2.87 g, 6.9 mmol), and PyAOP (3.60 g, 6.90 mmol) were dissolved in 30 mL of anhydrous DMF. Diisopropylethylamine (2.41mL, 13.81 mmol) was added, and the reaction mixture was stirred for 12 h. The reaction mixture was diluted with ethyl acetate (100 mL) and washed with water (2 x 100 mL) followed by washing with brine (100 mL). The organic layer was dried with anhydrous sodium sulfate and concentrated under reduced pressure. Crude product was purified by column chromatography (SiO_2, hexane: ethyl acetate = 10:1 to 3:1) to give **5a** (1.61g, 60%) as a white sticky solid. ^{1}H NMR (400 MHz, DMSO) δ (ppm) = 7.89 (dt, J = 7.7, 0.9 Hz, 2H), 7.72 (dd, J = 7.4, 3.1 Hz, 2H), 7.50 – 7.27 (m, 4H + 1H from amide), 7.18 (s, 1H), 7.17 (s, 1H), 4.79 – 4.01 (m, 3H), 3.90 (td, J = 8.8, 4.9 Hz, 1H), 2.37 – 2.02 (m, 14H), 1.79 (m, J = 9.5, 6.4 Hz, 14H), 1.38 (s, 36H), 1.34 (s, 36H). ^{13}C NMR (101 MHz, DMSO) δ (ppm) = 172.64, 172.61, 171.83, 171.81, 156.34, 144.33, 144.22, 141.20, 128.09, 127.49, 125.79, 125.71, 120.58, 80.01, 79.98, 66.19, 57.10, 56.89, 47.07, 29.55, 29.41, 29.37, 28.19, 28.14, 11.72. HRMS (ESI-MS): *m/z* calculated for $C_{64}H_{97}N_3O_{16}$ [M+H]$^+$:1164.6942; found: 1164.6934.

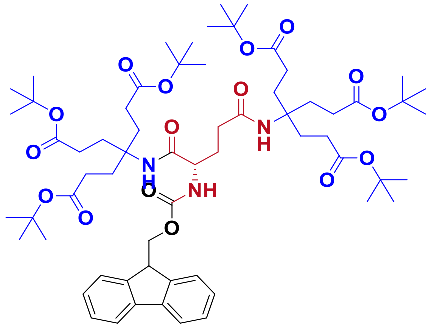

5a

Dendron [5-H]$^+$Cl$^-$. Compound **5a** (1.0 g, 0.86 mmol) was dissolved in a 60 mL of anhydrous dichloromethane and cooled to 0°C. Piperidine (12 mL) was slowly added, and the reaction mixture stirred for 2 h. The reaction mixture was diluted with 60 mL of dichloromethane and washed with water (2 x 25 mL) followed by brine (25 mL). The organic layer was dried with anhydrous sodium sulfate and the solvent removed under reduced pressure. The crude product was purified by column chromatography (SiO_2, from hexane: ethyl acetate = 1:1 to ethyl acetate: triethylamine = 100:1) to give dendron **5** as an oil; the chromatographic elution of **5** was monitored with ninhydrin stain. The oil was dissolved in dichloromethane and washed with 1M HCl. The organic layer was dried with anhydrous sodium sulfate and the solvent was evaporated under reduced pressure to give **[5-H]$^+$Cl$^-$** (480 mg 59%) as a white solid. ^{1}H NMR (400 MHz, DMSO) δ (ppm) = 8.12 (s, 3H), 7.85 (s, 1H), 7.36 (s, 1H), 3.77 (m, 1H), 2.39 – 1.99 (m, 14H), 2.02 – 1.73 (m, 14H), 1.39 (two singlets, 54H). ^{13}C NMR (101 MHz, DMSO) δ (ppm) = 172.12, 172.06, 170.90, 167.78, 79.63, 79.57, 57.40, 56.71, 52.22, 29.07, 28.99, 28.89, 28.83, 27.73, 27.70. HRMS (ESI-MS): *m/z* calculated for $C_{49}H_{87}N_3O_{14}$ [M+H]$^+$:942.6261; found: 942.6256.

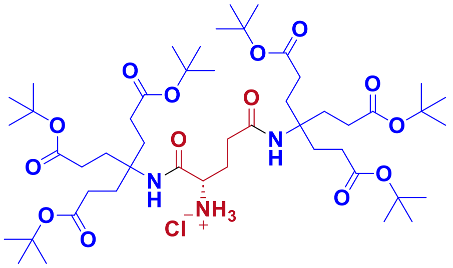

[5-H]$^+$Cl$^-$

Compound 6a. Behera's amine (1.0 g, 2.41 mmol) and sodium bicarbonate (303 mg, 3.61 mmol) were dissolved in 40 mL of 1:1 mixture of water and acetonitrile thereafter cooled to 0 ºC. Next, a solution of Fmoc-succinimide (974 mg, 2.89 mmol) in acetonitrile (20 mL) was slowly added over 15 minutes. The reaction mixture was warmed to room temperature and stirred for 12 h. After a dilution with 200 mL of ethyl acetate and washing with water (2 x 100mL) in a separatory funnel, the organic layer was collected, dried with anhydrous sodium sulfate, and concentrated under reduced pressure. Crude product was purified by column chromatography (SiO_2, hexane: ethyl acetate = 6:1) to give **6a** (1.3 g, 86 %) as a white solid. ^{1}H NMR (400 MHz, DMSO) δ (ppm) = 7.89 (dt, J = 7.6, 1.0 Hz, 2H), 7.75 – 7.69 (m, 2H), 7.42 (td, J = 7.5, 1.2 Hz, 2H), 7.33 (td, J = 7.5, 1.2 Hz, 2H), 6.99 (s, 1H), 4.45 – 3.90 (m, 3H), 2.11 (t, J = 8.0 Hz, 6H), 1.77 (t, J = 8.2 Hz, 6H), 1.38 (s, 27H). ^{13}C NMR (101 MHz, DMSO) δ (ppm) = 172.59, 154.56, 144.35, 141.19, 128.06, 127.47, 125.71, 120.58, 80.02, 65.23, 56.38, 47.25, 29.53, 29.41, 28.19. HRMS (ESI-MS): *m/z* calculated for $C_{37}H_{51}NO_8$ [M+H]$^+$: 638.3687; found: 638.3679.

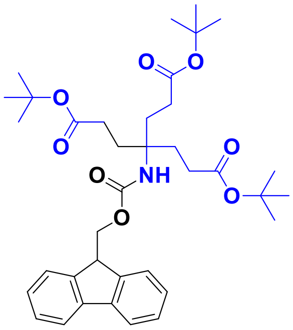

6a

Compound 6b. Compound **6a** (1.0 g) was dissolved in 50 mL of dichloromethane: trifluoracetic acid = 10:1 and stirred overnight at room temperature. The solvent was removed under reduced pressure to give an oily solid. The crude product was suspended in 10 mL of diethyl ether followed by centrifugation to give a white solid. The solid was dried under vacuum to give **6b** (721 mg, 98%) as a white powder. ^{1}H NMR (400 MHz, DMSO) δ (ppm) = 11.94 (s, 3H), 7.80 (dt, J = 7.5 Hz, 2H), 7.63 (dt, J = 7.4 Hz, 2H), 7.28 (td, J = 34.8, 7.4, 1.1 Hz, 2H), 6.92 (s, 1H), 4.13 (m, 3H), 2.05 (t, J = 8.0 Hz, 6H), 1.72 (t, J = 8.2 Hz, 6H). ^{13}C NMR (101 MHz, DMSO) δ (ppm) = 174.85, 154.60, 144.38, 141.17, 128.09, 127.51, 125.75, 120.56, 65.31, 56.22, 47.25, 29.58, 28.50, 28.20. HRMS (ESI-MS): *m/z* calculated for $C_{25}H_{27}NO_8$ [M+H]$^+$: 470.1809; found: 470.1816.

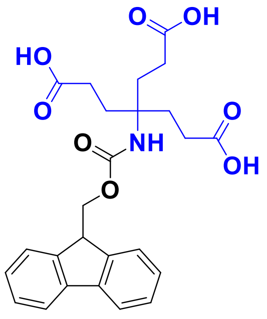

6b

Compound 6c. Compound **6b** (500 mg 1.06 mmol), di-tert-butyl-(*S*)-glutamate hydrochloride (1.57 g, 5.32 mmol) and PyAOP (2.0 g 5.32 mmol) were dissolved in 25 mL of anhydrous DMF. Diisopropylethylamine (1.57 mL, 8.48 mmol) was added, and the reaction mixture was stirred for 12 h. The reaction mixture was diluted with ethyl acetate (100 mL) and washed with water (2 x 100 mL) followed by brine (100 mL) in a separatory funnel. The organic layer was dried with sodium sulfate and concentrated under reduced pressure. The crude product was purified by column chromatography (SiO_2, hexane: ethyl acetate = from 10:1 to 1:1) to give **6c** (800 mg, 63 %) as a colorless oil. ^{1}H NMR (400 MHz, DMSO) δ (ppm) = 7.90 – 7.88 (m, 3H), 7.74 (d, *J* = 7.4 Hz, 1H), 7.41 (td, *J* = 7.4 1.3 Hz, 2H), 7.34 (td, *J* = 7.4, 1.3 Hz, 2H), 7.08 (s, 1H), 4.37 – 4.00 (m, 5H), 2.43 – 2.17 (m, 6H), 2.20 – 2.04 (m, 6H), 1.96 – 1.67 (m, 12H), 1.39 (s, 27H), 1.39 (s, 27H). ^{13}C NMR (101 MHz, DMSO) δ 175.37, 173.31, 172.77, 172.50, 171.88, 171.61, 171.57, 169.85, 154.58, 144.42, 144.40, 141.14, 137.89, 129.39, 128.06, 127.75, 127.56, 125.87, 121.85, 120.53, 120.50, 80.94, 80.26, 65.50, 56.50, 54.24, 52.23, 52.18, 47.23, 31.72, 31.57, 28.22, 28.19, 28.16, 28.10, 26.81. HRMS (ESI-MS): *m*/*z* calculated for $C_{64}H_{96}N_4O_{17}$ $[M+H]^+$:1193.6843; found: 1193.6831.

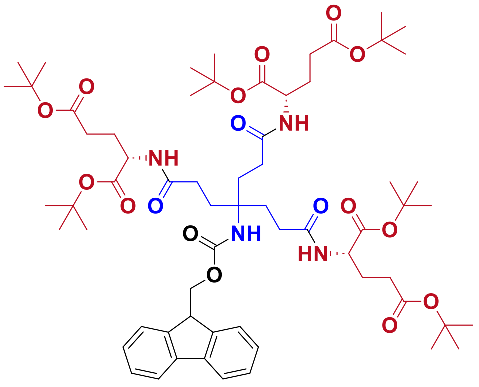

6c

Dendron $[6\text{-H}]^+Cl^-$. Compound **6c** (500 mg, 0.42 mmol) was dissolved in a 60 mL of anhydrous dichloromethane and the solution was cooled to 0°C. Piperidine (12 mL) was added at a slow rate, and the reaction mixture stirred for 2 h. After a dilution with 60 mL of dichloromethane, washing with water (2 x 25 mL), and brine (25 mL), the organic layer was dried with anhydrous sodium sulfate and concentrated under reduced pressure. Crude product was purified by column chromatography (SiO_2, hexane: ethyl acetate = 1:1 to ethyl acetate: triethylamine = 100:1) to give dendron **6** as an oil; the chromatographic elution of **6** was monitored with ninhydrin stain. The oil was dissolved in dichloromethane and washed with 1N HCl in a separatory funnel. The organic layer was dried with sodium sulphate and the solvent was evaporated under reduced pressure to give dendron **$[6\text{-H}]^+Cl^-$** (215 mg, 51%) as a white solid. ^{1}H NMR (400 MHz, DMSO) δ (ppm) = 8.30 (d, *J* = 7.5 Hz, 3H), 8.02 (s, 3H), 4.12 (ddd, *J* = 9.1, 7.4, 5.3 Hz, 3H), 2.36 – 2.18 (m, 12H), 2.04 – 1.62 (m, 12H), 1.40 (s, 54H). ^{13}C NMR (101 MHz, DMSO) δ (ppm) = 171.9, 171.8, 171.4, 81.1, 80.3, 52.3, 31.8, 31.6, 29.1, 28.2, 28.1, 26.7. HRMS (ESI-MS): m/z calculated for $C_{49}H_8N_4O_{15}$ $[M+H]^+$: 971.6162; found: 971.6168.

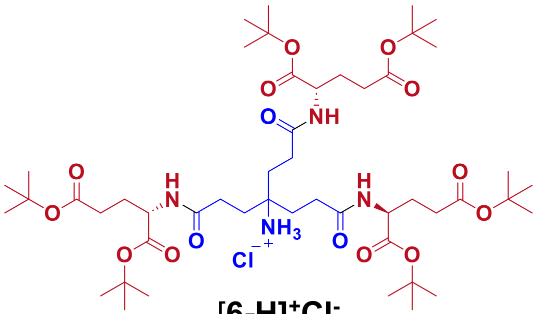

$[6\text{-H}]^+Cl^-$

Characterization of Dendrons 4-6

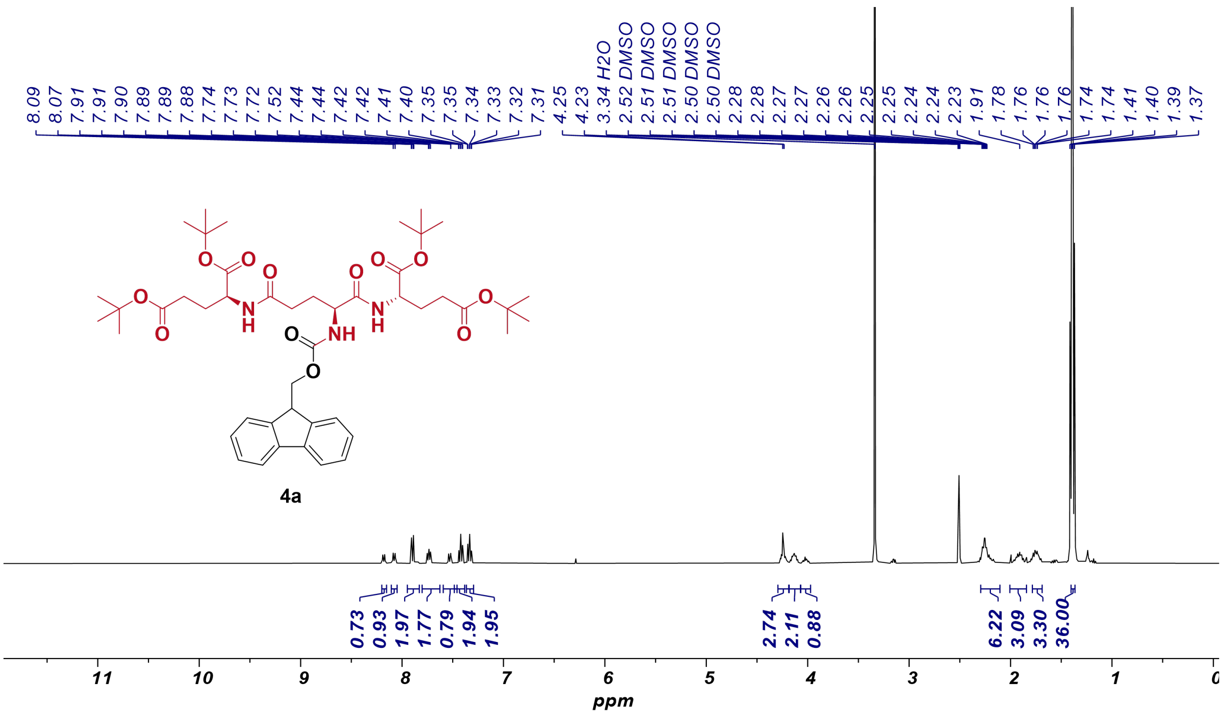

Figure S19. ^{1}H NMR spectrum (400 MHz, 298 K) of **4a** in DMSO-d$_6$.

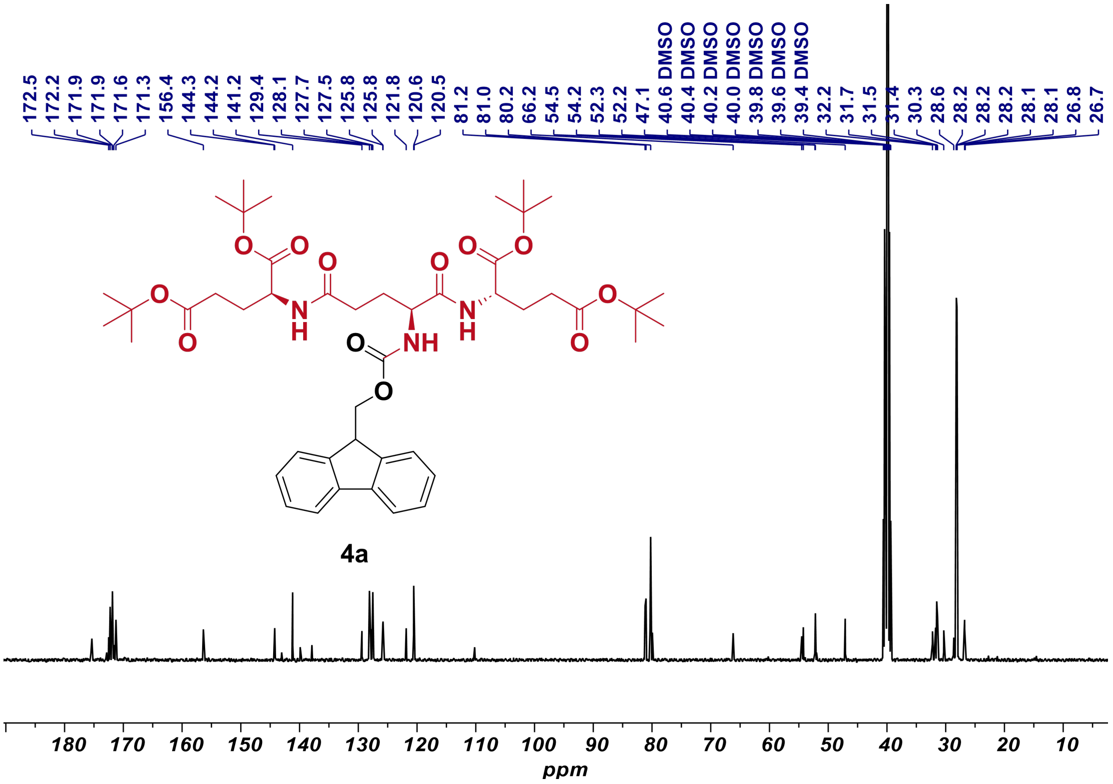

Figure S20. ^{13}C NMR spectrum (100 MHz, 298 K) of **4a** in DMSO-d$_6$.

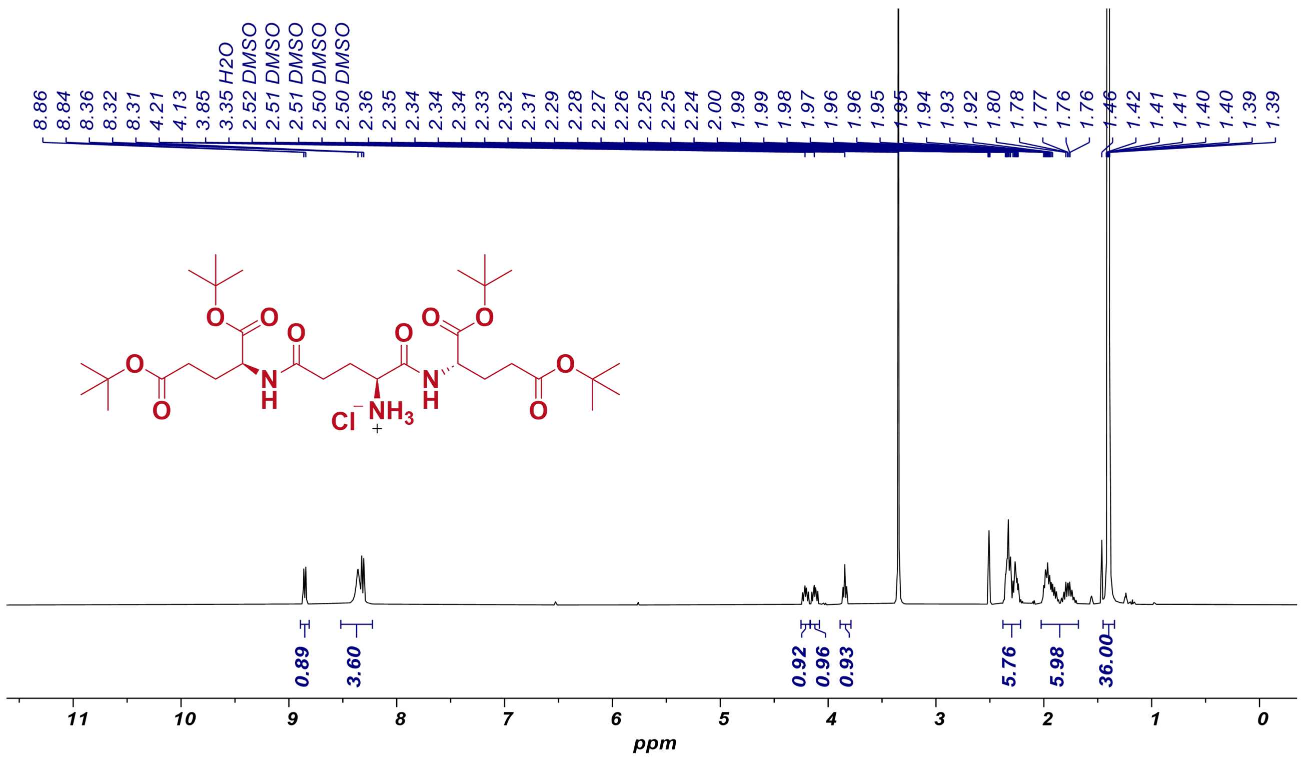

Figure S21. ^{1}H NMR spectrum (400 MHz, 298 K) of dendron **[4-H]$^{+}$Cl^{-}** in DMSO-d$_6$.

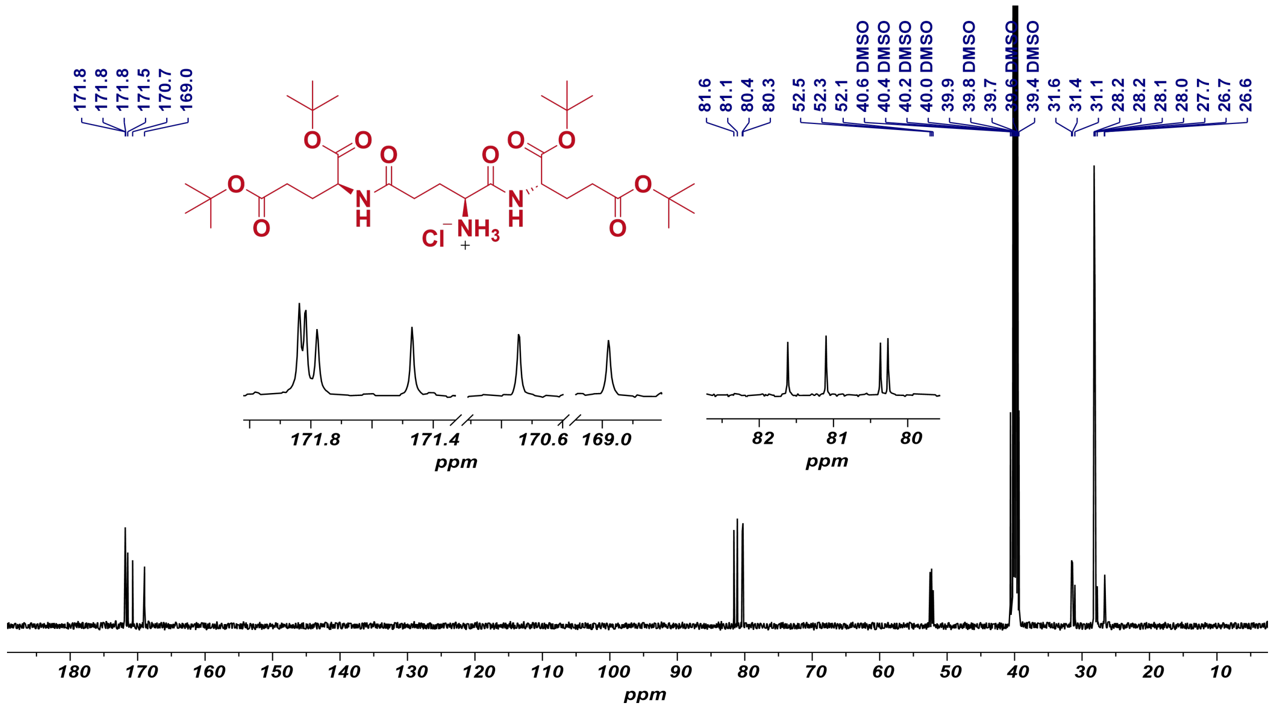

Figure S22. ^{13}C NMR spectrum (100 MHz, 298 K) of dendron **[4-H]$^{+}$Cl^{-}** in DMSO-d$_6$.

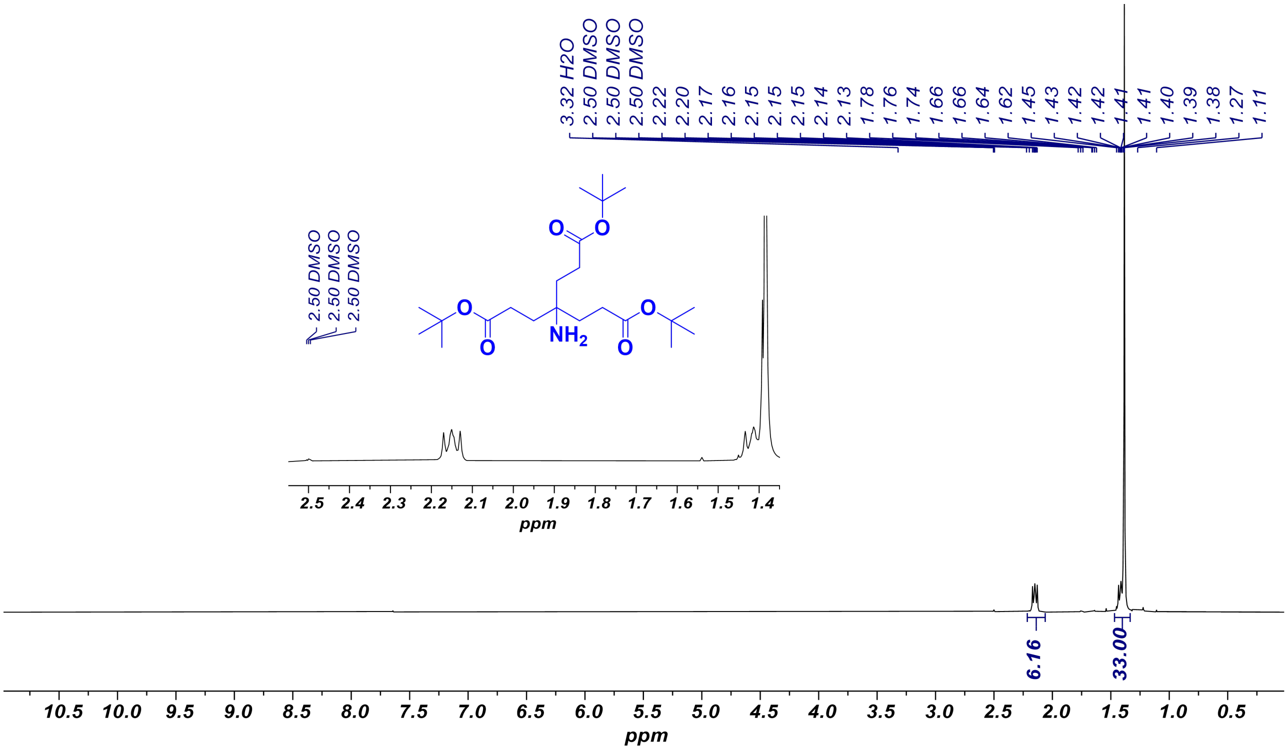

Figure S23. ^{1}H NMR spectrum (400 MHz, 298 K) of Behera's amine in DMSO-d_6.

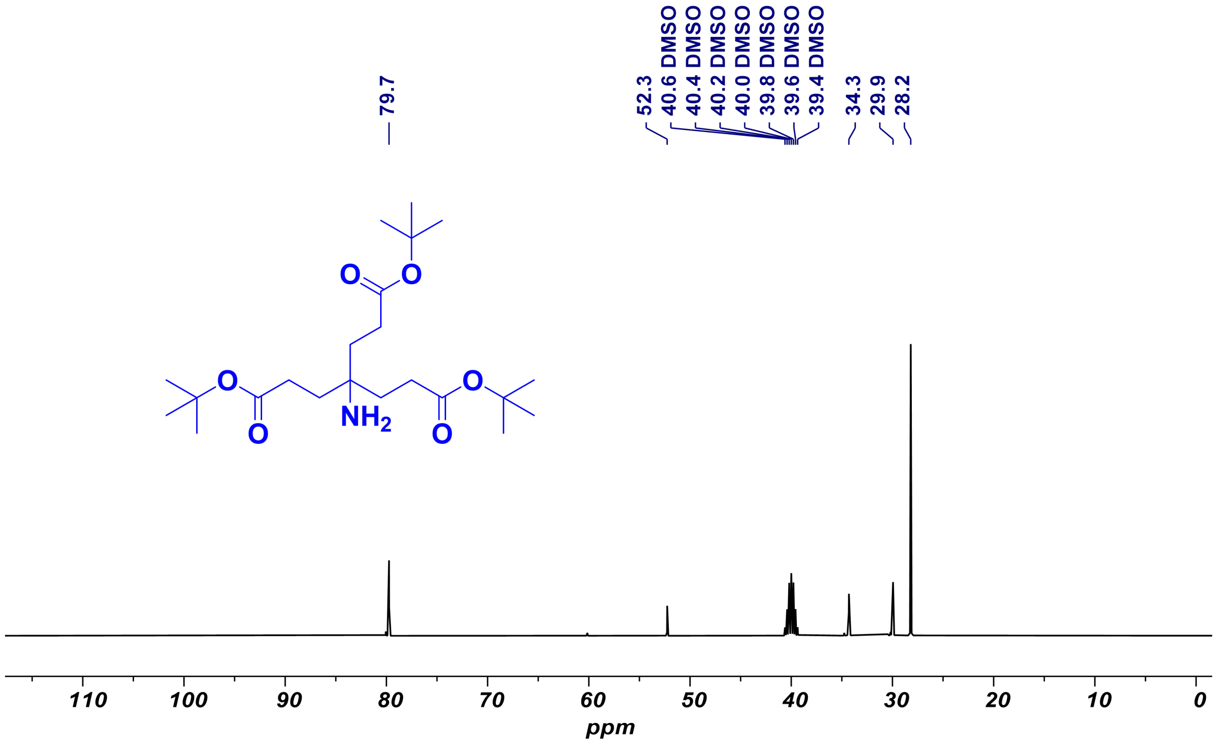

Figure S24. ^{13}C NMR spectrum (100 MHz, 298 K) of Behera's amine in DMSO-d_6.

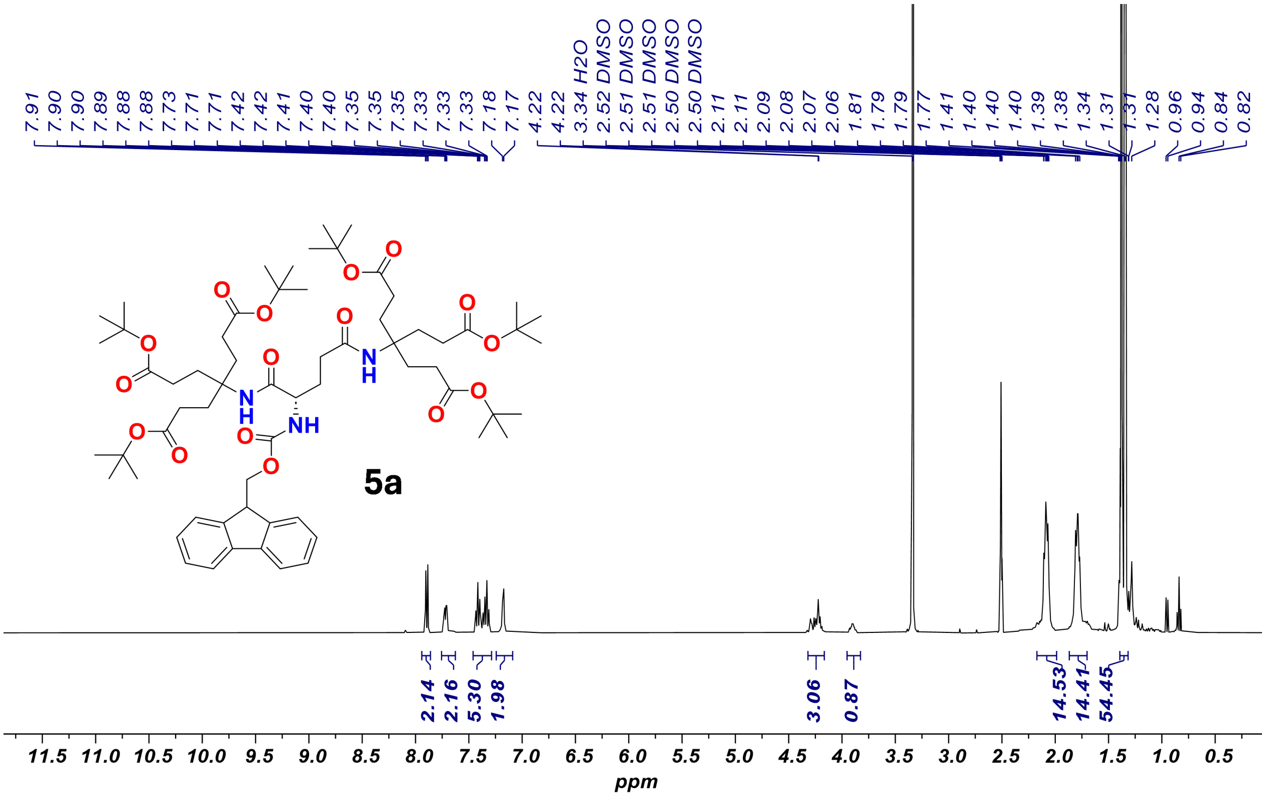

Figure S25. ^{1}H NMR spectrum (400 MHz, 298 K) of **5a** in DMSO-d$_6$.

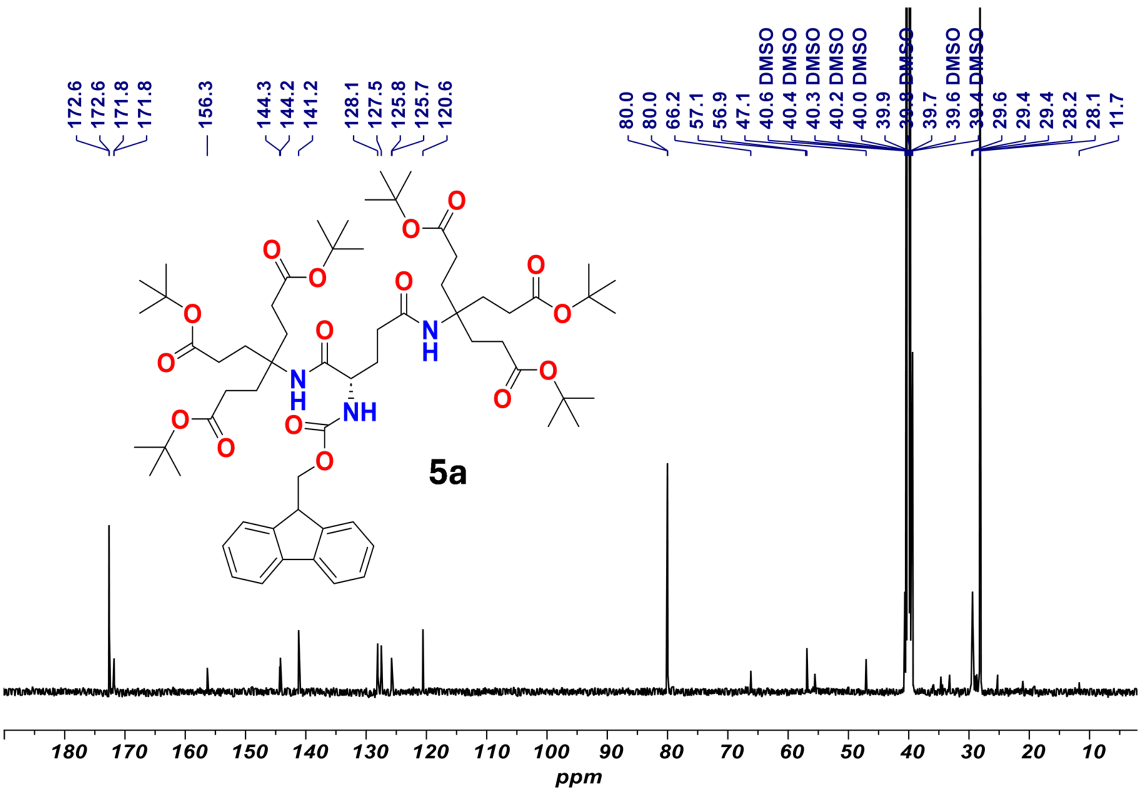

Figure S26. ^{13}C NMR spectrum (100 MHz, 298 K) of **5a** in DMSO-d$_6$.

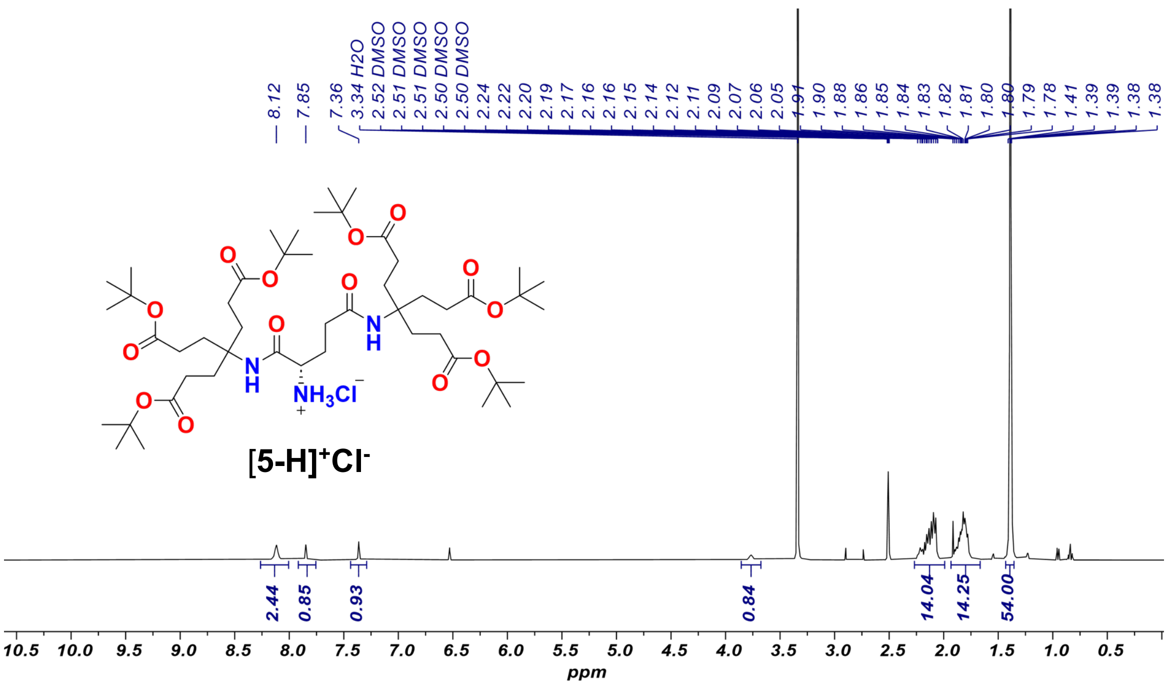

Figure S27. ^{1}H NMR spectrum (400 MHz, 298 K) of dendron **[5-H]$^{+}$Cl^{-}** in DMSO-d$_6$.

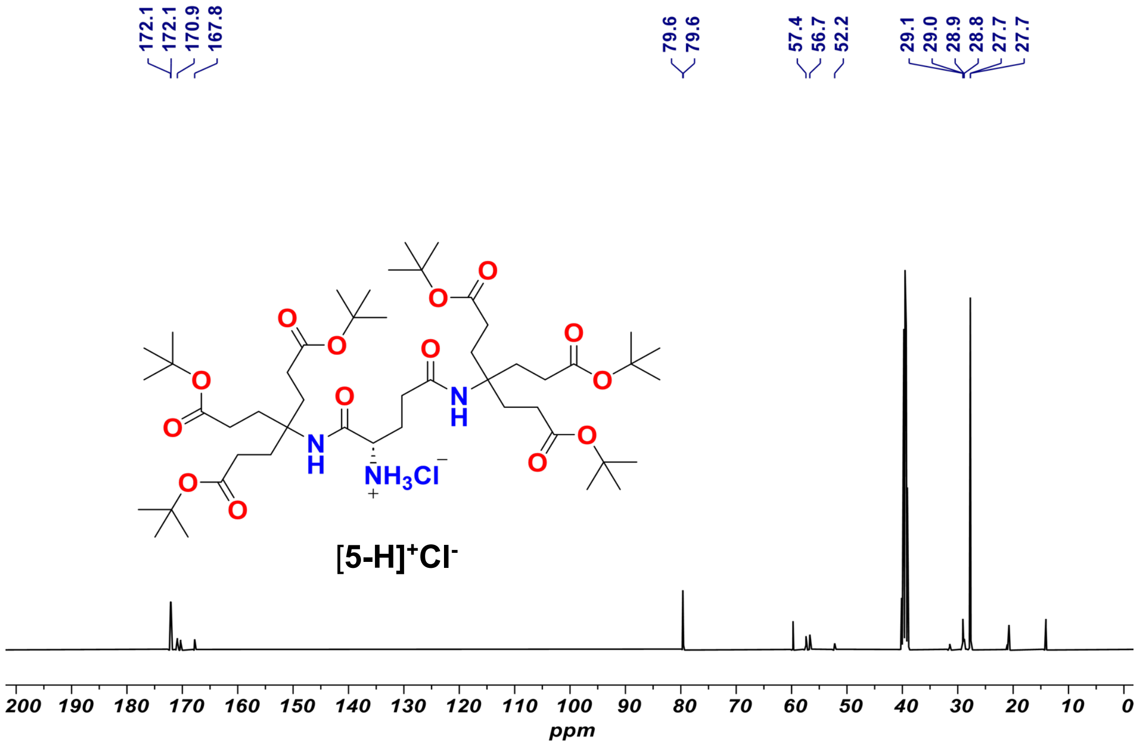

Figure S28. ^{13}C NMR spectrum (100 MHz, 298 K) of dendron **[5-H]$^{+}$Cl^{-}** in DMSO-d$_6$.

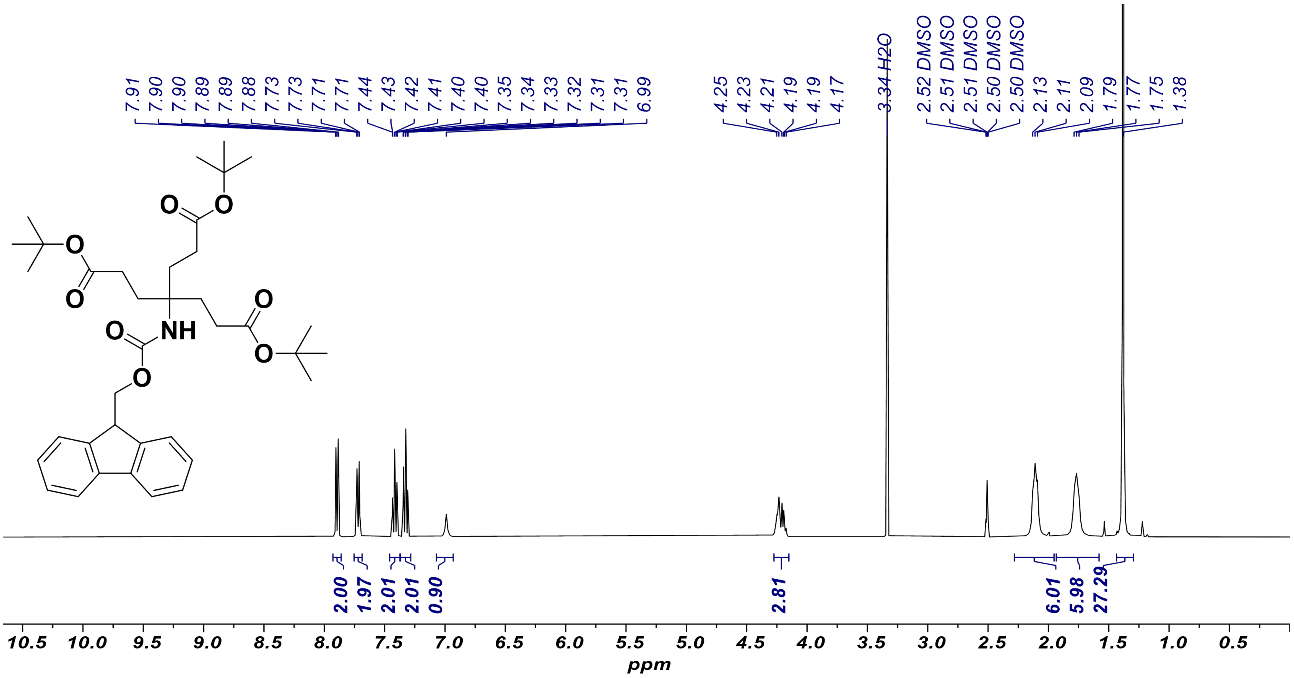

Figure S29. ^{1}H NMR spectrum (400 MHz, 298 K) of **6a** in DMSO-d_6.

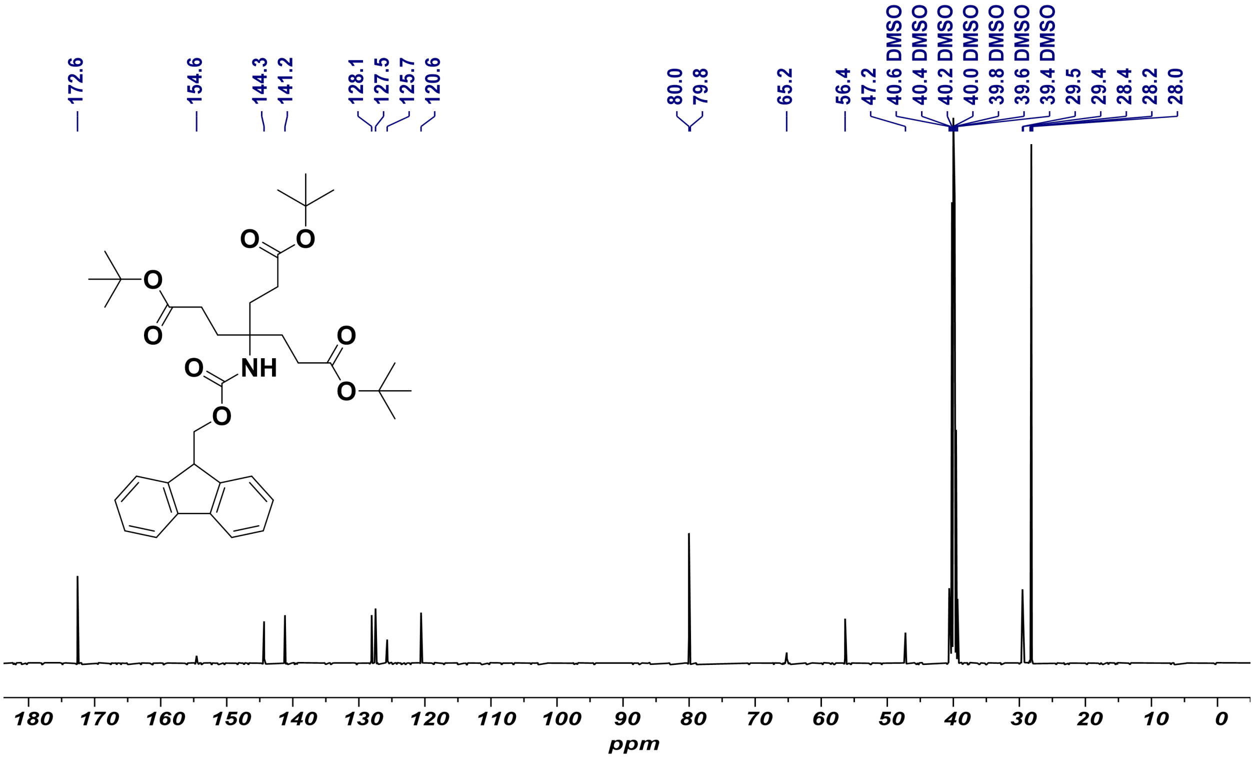

Figure S30. ^{13}C NMR spectrum (100 MHz, 298 K) of **6a** in DMSO-d_6.

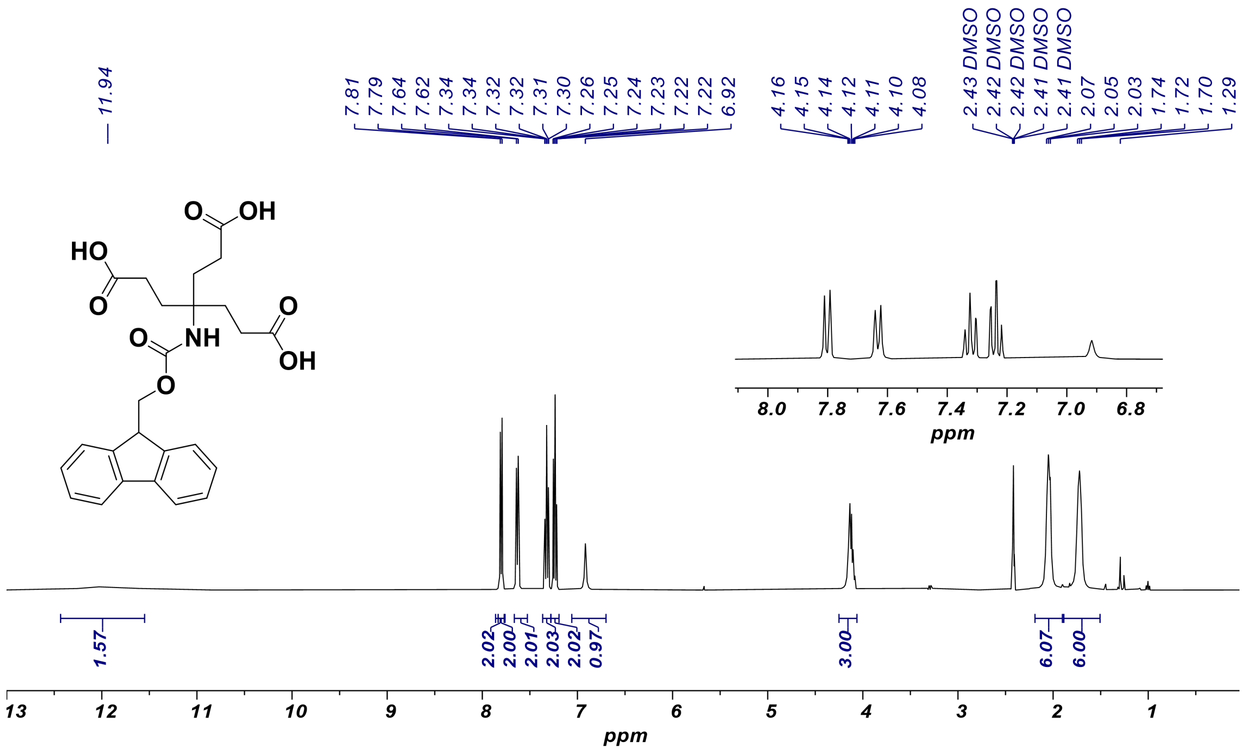

Figure S31. ^{1}H NMR spectrum (400 MHz, 298 K) of **6b** in DMSO-d$_6$.

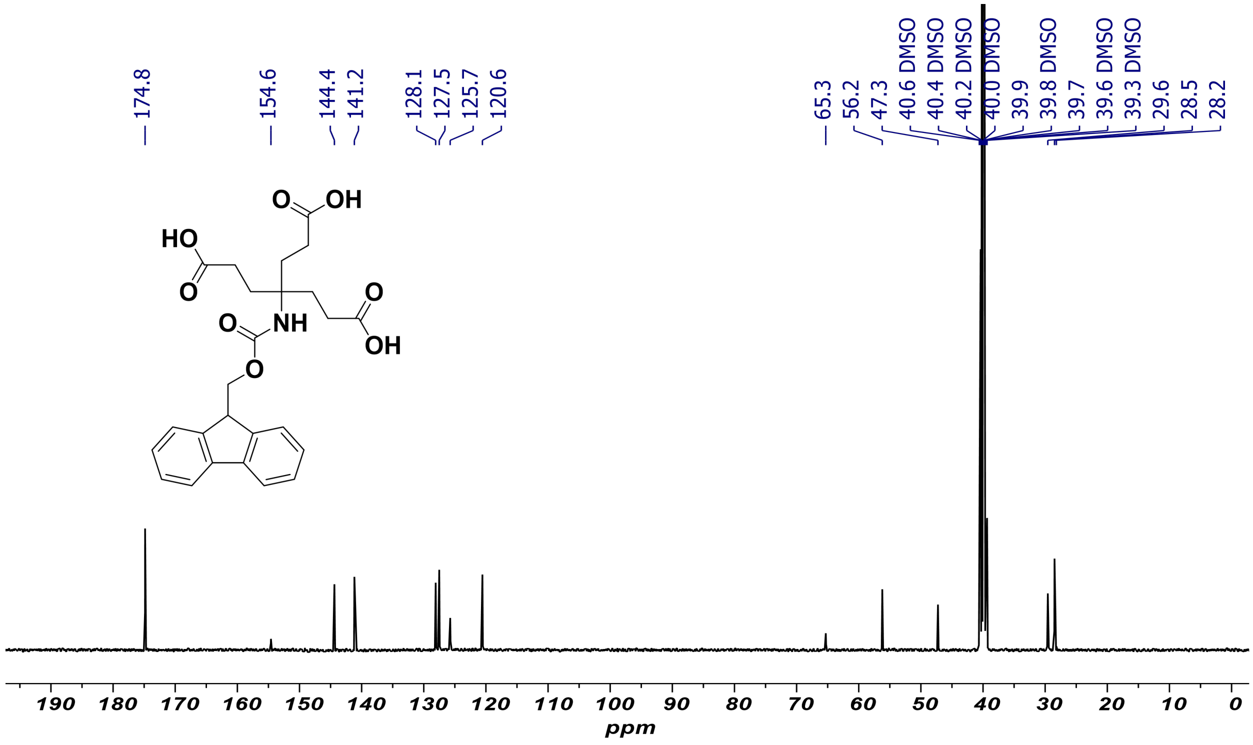

Figure S32. ^{13}C NMR spectrum (100 MHz, 298 K) of **6b** in DMSO-d$_6$.

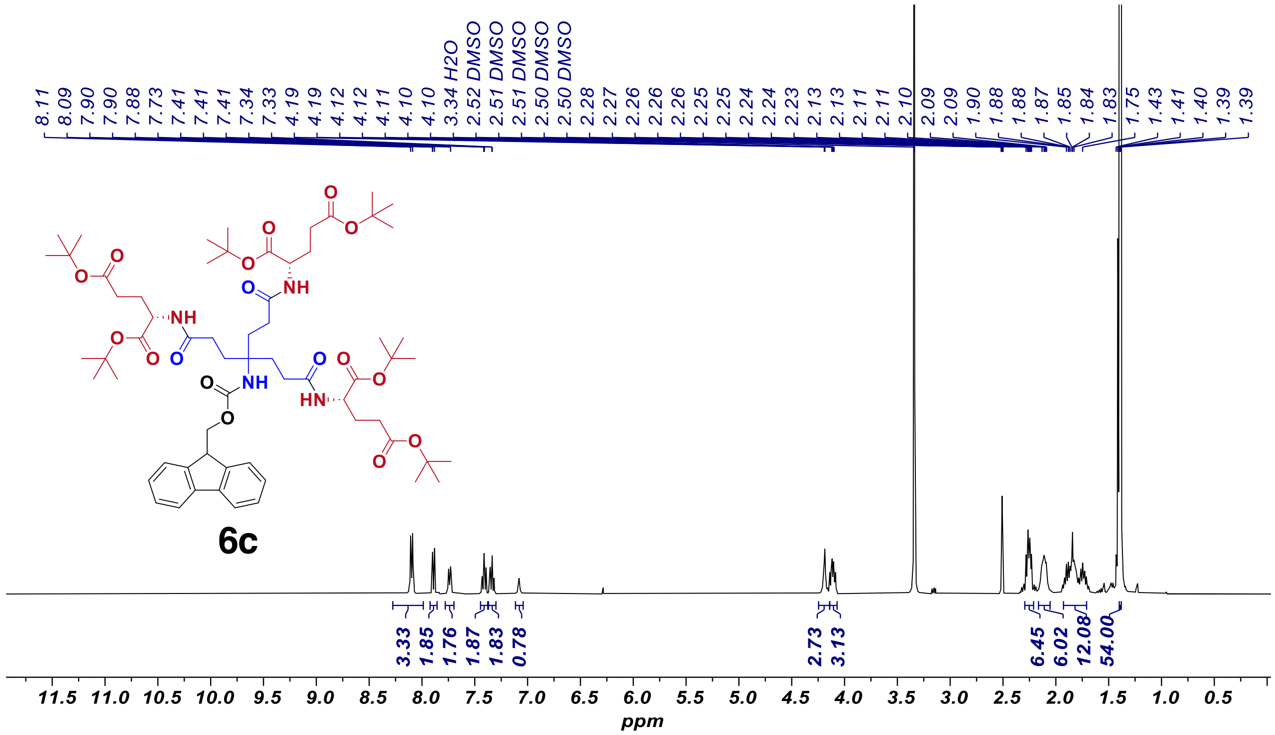

Figure S33. ^{1}H NMR spectrum (400 MHz, 298 K) of **6c** in DMSO-d_6.

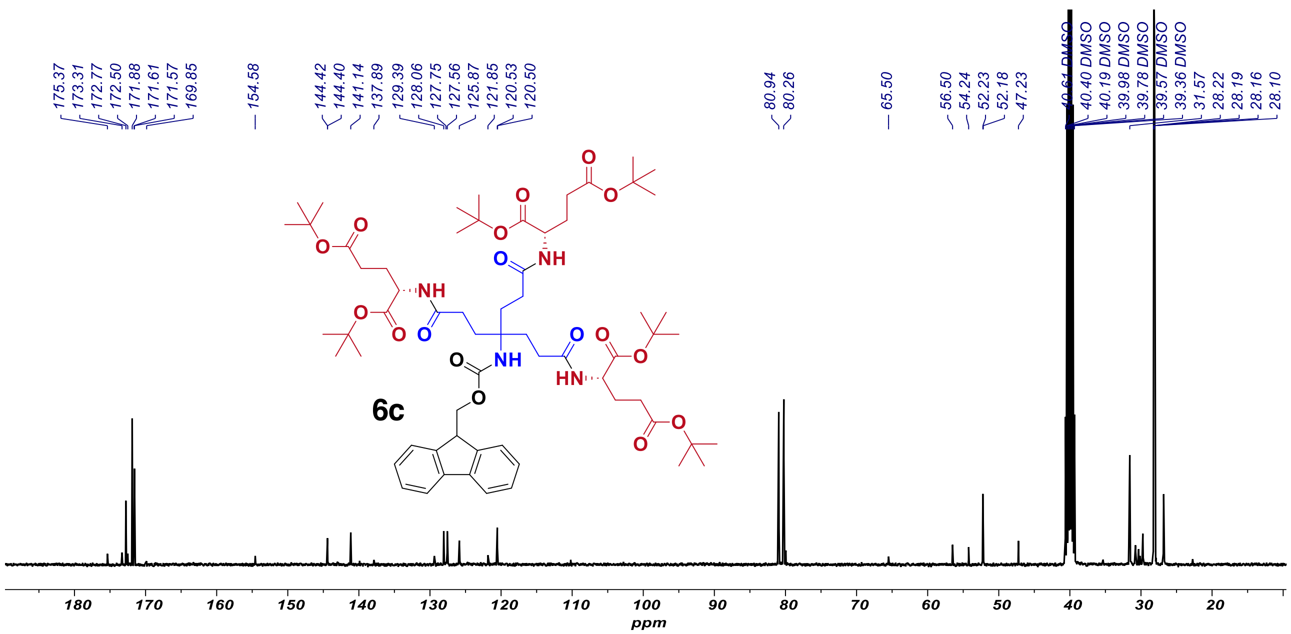

Figure S34. ^{13}C NMR spectrum (100 MHz, 298 K) of **6c** in DMSO-d_6.

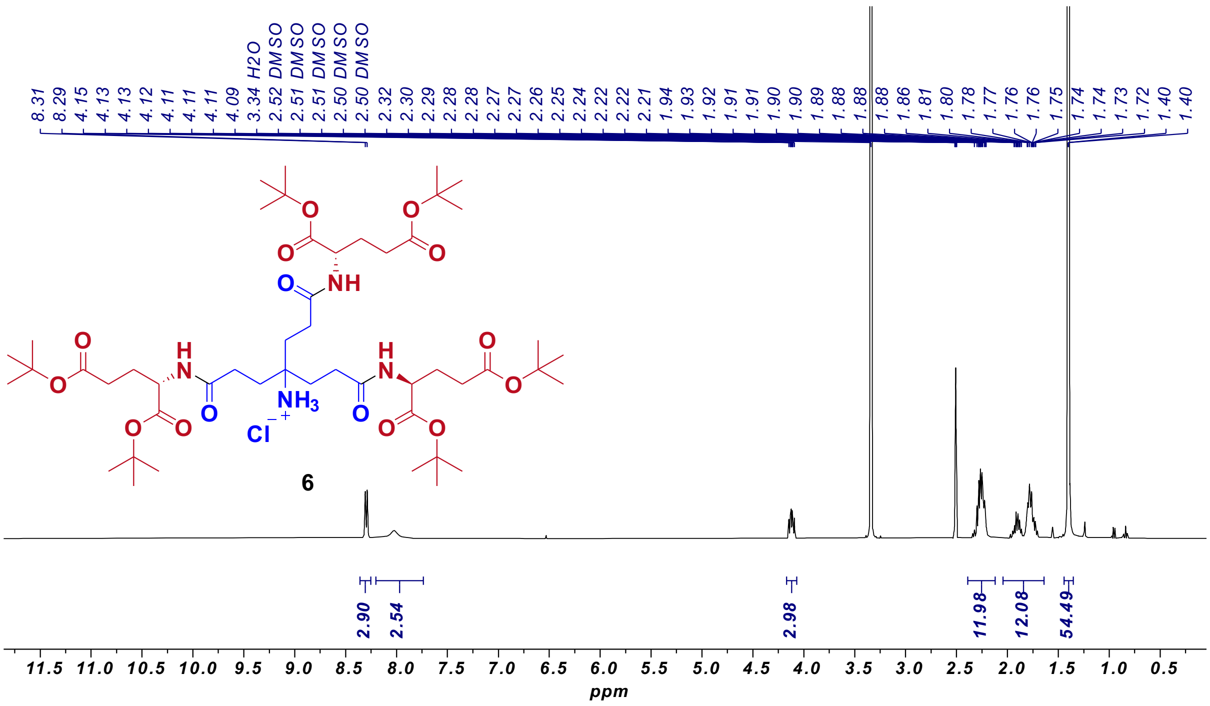

Figure S35. ^{1}H NMR spectrum (400 MHz, 298 K) of dendron **[6-H]$^{+}$Cl^{-}** in DMSO-d_6.

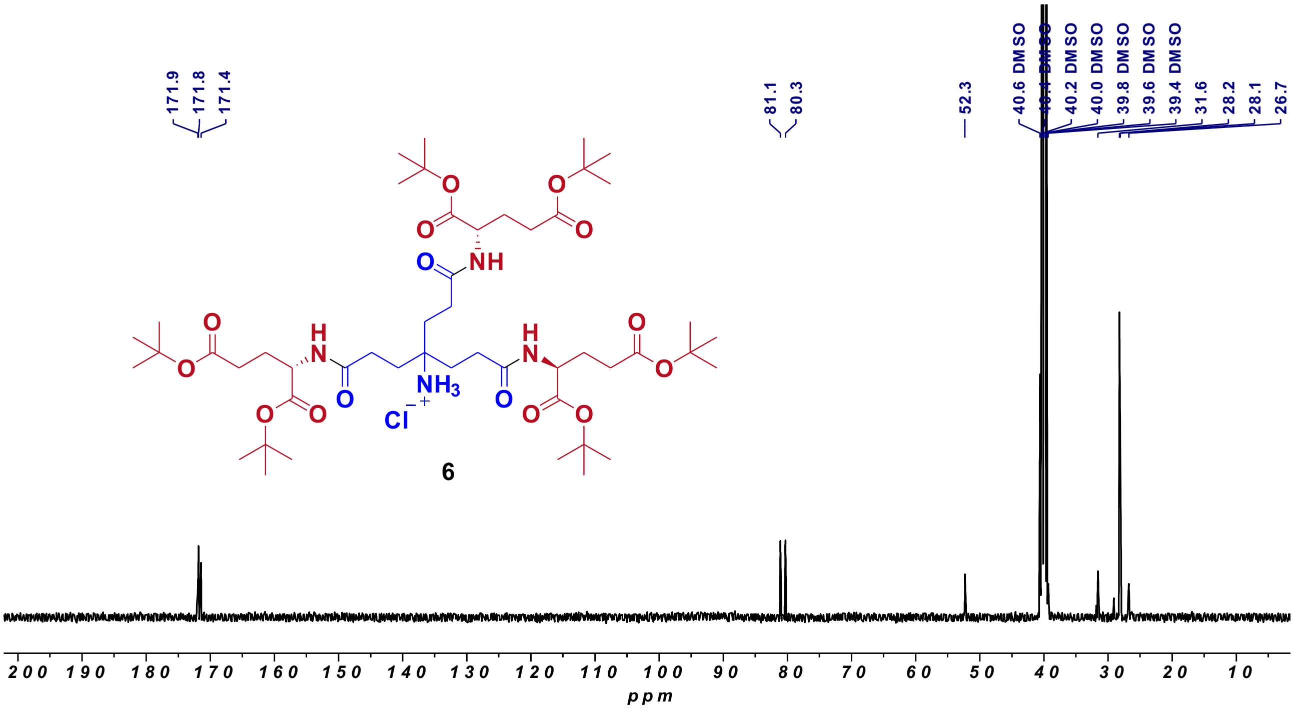

Figure S36. ^{13}C NMR spectrum (100 MHz, 298 K) of dendron **[6-H]$^{+}$Cl^{-}** in DMSO-d_6.

Synthesis and Resolution of Dendritic Pillar[6]Arenes *pS*/*pR*-11/12/13

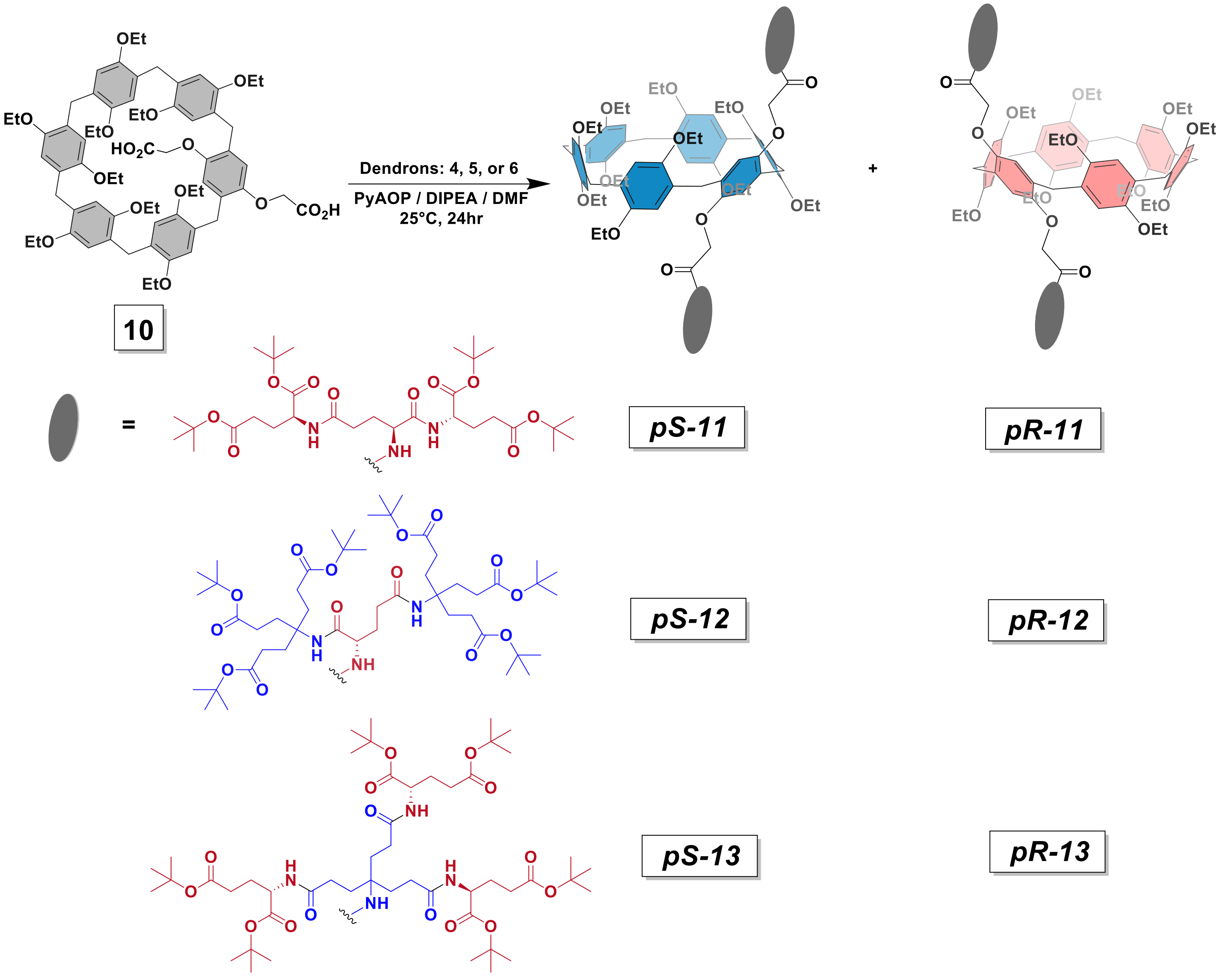

Scheme S3. Synthetic scheme describing the preparation of dendritic pillar[6]arenes *pS*/*pR*-**11**, *pS*/*pR*-**12**, and *pS*/*pR*-**13**.

Dendritic pillar[6]arenes *pR*-11 and *pS*-11. Pillar[6]arene **10** (25 mg, 0.02 mmol), dendron **$[4\text{-H}]^+Cl^-$** (59 mg, 0.09 mmol), and PyAOP reagent (46.2 mg, 0.09 mmol) were dissolved in 2.0 mL of anhydrous DMF. Diisopropylethylamine (30 μL, 0.172 mmol) was added and the reaction mixture was stirred at room temperature for 24 h. The reaction mixture was diluted with 10 mL of dichloromethane and washed with water (3 x 10 mL). The organic layer was dried with anhydrous sodium sulfate and concentrated under reduced pressure. Crude products were subjected to column chromatography (SiO_2, dichloromethane: acetone = 8:1) to give dendritic pillar[6]arenes *pR*-**11** (12.8 mg, R_f = 0.85) and *pS*-**11** (15.7 mg, R_f = 0.61) as colorless sticky oils (overall yield 55%).

Dendritic pillar[6]arene *pR*-11. ^{1}H NMR (600 MHz, CD_2Cl_2) δ (ppm) = 8.13 (d, *J* = 8.7 Hz, 2H), 7.77 (d, *J* = 8.7 Hz, 2H), 7.24 (d, *J* = 7.8 Hz, 2H), 6.74 (s, 2H), 6.72 (s, 4H), 6.71 (s, 2H), 6.64 (s, 2H), 6.63 (s, 2H),4.61 (td, *J* = 9.3, 4.5 Hz, 2H), 4.53 (td, *J* = 9.1, 4.6 Hz, 2H), 4.37 – 4.31 (m, 4H), 4.32 (d, *J* = 14.5 Hz, 2H), 4.22 (d, *J* = 14.6 Hz, 2H), 3.88 – 3.78 (m, 28H), 3.66 (d, *J* = 14.4 Hz, 2H), 2.42 – 2.26 (m, 12H), 2.22 – 2.09 (m, 6H), 1.98 – 1.84 (m, 6H), 1.53 (s, 18H), 1.52 (s, 18H), 1.47 (s, 18H), 1.44 (s, 18H), 1.36 – 1.29 (m, 18H), 1.27 – 1.22 (m, 12H).^{13}C NMR (151 MHz, CD_2Cl_2) δ (ppm) = 174.24, 173.91, 172.71, 171.98, 171.96, 171.42, 167.78, 151.22, 150.91, 150.77, 150.69, 150.67, 150.11, 128.98, 128.87, 128.14, 128.12, 128.05, 127.70, 116.15, 116.13, 115.66, 115.44, 115.05, 114.95, 83.37, 80.80, 80.71, 68.61, 64.86, 64.78, 64.54, 64.38, 64.34, 32.40, 32.13, 31.89, 31.53, 31.27, 29.75, 28.20, 28.18, 28.17, 28.14, 27.39, 27.18, 15.42, 15.38, 15.33, 15.29. HRMS (ESI-MS) *m/z* calculated for $C_{128}H_{186}N_6O_{34}$ $[M+2H]^{2+}$: 1176.6578; found: 1176.6585.

Dendritic pillar[6]arene *pS*-11. ^{1}H NMR (600 MHz, CD_2Cl_2) δ (ppm) = 8.10 (d, *J* = 8.6 Hz, 2H), 7.70 (d, *J* = 8.6 Hz, 2H), 7.10 (d, *J* = 7.7 Hz, 2H), 6.70 (s, 2H), 6.67 (s, 4H), 6.64 (s, 2H), 6.62 (s, 2H), 6.59 (s, 2H), 4.56 (td, *J* = 9.0, 4.7 Hz, 2H), 4.49 (td, *J* = 9.1, 4.7 Hz, 2H), 4.34 – 4.20 (m, 6H), 4.03 (d, *J* = 14.3 Hz, 2H), 3.82 – 3.74 (m, 28H), 3.62 (d, *J* = 14.3 Hz, 2H), 2.40 – 2.27 (m, 12H), 2.15 – 2.04 (m, 6H), 1.94 – 1.78 (m, 6H), 1.49 (s, 18H), 1.48 (s, 18H), 1.43 (s, 18H), 1.41 (s, 18H), 1.31 – 1.20 (m, 30H). ^{13}C NMR (214 MHz, CD_2Cl_2) δ (ppm) = 174.18, 173.77, 172.83, 172.81, 172.00, 171.93, 171.32, 167.98, 151.66, 151.00, 150.89, 150.82, 150.77, 150.63, 150.34, 129.29, 128.59, 128.19, 128.18, 128.05, 127.37, 116.39, 115.59, 115.57, 115.48, 115.27, 114.86, 83.40, 83.38, 80.82, 80.75, 68.94, 64.53, 64.52, 64.51, 64.42, 64.41, 52.83, 52.58, 51.67, 32.33, 32.13, 31.90, 31.47, 31.35, 31.30, 29.48, 28.21, 28.20, 28.18, 28.14, 27.43, 27.20, 25.91, 25.26, 23.10, 15.42, 15.40, 15.37. HRMS (ESI-MS) *m/z* calculated for $C_{128}H_{186}N_6O_{34}$ $[M+2H]^{2+}$: 1176.6578; found: 1176.6585.

Dendritic pillar[6]arenes *pS*-12 and *pR*-12. Pillar[6]arene **10** (25 mg, 0.02 mmol), dendron **$[5\text{-H}]^+Cl^-$** (86 mg, 0.09 mmol) and PyAOP reagent (46.2 mg, 0.09 mmol) were dissolved in 2.5 mL of anhydrous DMF. Diisopropylethylamine (30 μL, 0.172 mmol) was added and the reaction mixture was stirred at room temperature for 24 h. The reaction mixture was diluted with 10 mL of dichloromethane and washed with water (3 x 10 mL). The organic layer was dried with anhydrous sodium sulfate and concentrated under reduced pressure. Crude products were subjected to column chromatography (SiO_2, hexane: ethyl acetate = 3:1 to 1:1) to give dendritic pillar[6]arenes *pR*-**12** (14.0 mg, R_f = 0.41) and p*S*-**12** (15.3 mg, R_f = 0.34) as colorless sticky oils (overall yield 44%).

Dendritic pillar[6]arene *pR*-12. ^{1}H NMR (600 MHz, CD_2Cl_2) δ (ppm) = 7.76 (d, *J* = 7.4 Hz, 2H), 6.85 (s, 2H), 6.73 (s, 2H), 6.69 (s, 2H), 6.68 (s, 4H), 6.67 (s, 2H), 6.65 (s, 2H), 6.63 (s, 2H), 6.61 (s, 2H), 4.34-4.32 (m , 4H), 4.25 (d, *J* = 14.6 Hz, 2H), 4.14 (d, *J* = 14.5 Hz, 2H), 3.86 – 3.71 (m, 28H), 3.67 (d, *J* = 14.5 Hz, 2H), 2.19 (m, 28H), 1.95 (m, 28H), 1.43 (s, 54H), 1.40 (s, 54H), 1.32 – 1.19 (m, 30H). ^{13}C NMR (151 MHz, CD_2Cl_2) δ (ppm) = 173.2, 172.7, 172.2, 170.4, 169.3, 151.1, 150.9, 150.8, 150.8, 150.8, 150.1, 128.9, 128.9, 128.1, 128.1, 128.1,

127.8, 116.1, 116.1, 115.6, 115.4, 115.2, 115.2, 80.8, 80.6, 68.5, 64.8, 64.8, 64.5, 64.4, 58.2, 58.2, 52.8, 34.2, 31.9, 31.5, 31.4, 31.4, 30.3, 30.0, 29.9, 29.9, 28.2, 28.2, 15.4, 15.4, 15.4, 15.4, 15.3. HRMS (ESI-MS) *m*/*z* calculated for $C_{164}H_{250}N_6O_{42}$ $[M+2H]^{2+}$:1488.8878; found: 1488.8901.

Dendritic pillar[6]arene *pS*-12. ^{1}H NMR (600 MHz, CD_2Cl_2) δ (ppm) = 7.63 (d, J = 7.5 Hz, 2H), 6.95 (s, 2H), 6.73 (s, 2H), 6.69 (s, 2H), 6.66 (s, 2H), 6.65 (s, 2H), 6.64 (s, 2H), 6.64 (s, 2H), 6.60 (s, 2H), 4.47 – 4.38 (m, 2H), 4.37 – 4.28 (m, 2H), 4.04 (d, J = 14.0 Hz, 2H), 3.89 – 3.74 (m, 26H), 3.72 (d, J = 14.2 Hz, 2H), 3.66 (d, J = 13.9 Hz, 2H), 2.20 (m,28H), 2.08 – 1.77 (m, 28H), 1.43 (s, 54H), 1.40 (s, 54H), 1.30 – 1.23 (m, 30H). ^{13}C NMR (151 MHz, CD_2Cl_2) δ (ppm) = 173.24, 172.72, 172.27, 170.36, 169.44, 150.90, 150.85, 150.84, 150.83, 150.70, 150.33, 129.25, 128.56, 128.25, 128.18, 127.95, 127.24, 116.30, 115.47, 115.41, 115.38, 115.32, 115.08, 80.78, 80.56, 68.89, 64.45, 64.43, 64.36, 58.29, 58.21, 52.63, 34.13, 32.38, 31.61, 31.38, 30.33, 30.04, 29.96, 29.92, 28.25, 28.21, 15.50, 15.41, 15.38. HRMS (ESI-MS) *m*/*z* calculated for $C_{164}H_{250}N_6O_{42}$ $[M+2H]^{2+}$:1488.8878; found: 1488.8901.

Dendritic pillar[6]arenes *pS*-13/*pR*-13. Pillar[6]arene **10** (15 mg, 0.013 mmol), dendron **$[6\text{-H}]^+Cl^-$** (52 mg 0.05 mmol) and PyAOP reagent (28 mg, 0.05 mmol) were dissolved in 2.5 mL of anhydrous DMF. Diisopropylethylamine (18.5 µL, 0.106 mmol) was added and the reaction mixture was stirred at room temperature for 24 h. The reaction mixture was diluted with 10 mL of dichloromethane and washed with water (3 x 10 mL). The organic layer was dried with anhydrous sodium sulfate and concentrated under reduced pressure. Crude products were subjected to column chromatography (SiO_2, dichloromethane: acetone = 6:1) to give a mixture of dendritic pillar[6]arenes *pS*/*pR*-**13** (R_f = 0.30) as a colorless sticky oil (17.3 mg, 43%). ^{1}H NMR (600 MHz, CD_2Cl_2) δ (ppm) = 6.84 (d, J = 7.9 Hz), 6.71 (s), 6.70 (s), 6.69 (d, J = 1.4 Hz), 6.68 (s), 6.66 (d, J = 2.0 Hz), 6.64 (s), 6.64 (s), 6.61 (s), 6.60 (s), 6.58 (s), 6.57 (s), 6.48 (s), 6.22(s)(40 H , including amide-H and aromatic-H from both the diastereomers), 4.41 (m, 12H), 4.33 – 4.19 (m, 8H), 4.05 (m, 4H), 3.93 – 3.71 (m, 56H), 3.55 (m, 4H), 2.44 – 1.76 (m, 96H), 1.45 (2 singlets merged 108H), 1.42 (2 singlets merged 108H), 1.35 – 1.13 (m, 60H). ^{13}C NMR (214 MHz, CD_2Cl_2) δ (ppm) = 172.68, 172.59, 172.34, 172.28, 171.92, 171.72, 168.37, 168.16, 151.11, 151.04, 150.88, 150.85, 150.81, 150.68, 149.88, 149.57, 130.32, 130.15, 128.80, 128.72, 128.61, 128.26, 128.21, 128.14, 128.07, 128.00, 127.85, 127.59, 115.94, 115.76, 115.68, 115.60, 115.53, 115.42, 115.19, 115.16, 115.06, 82.38, 82.35, 80.75, 80.72, 68.53, 68.09, 64.81, 64.77, 64.64, 64.49, 64.47, 64.41, 64.39, 58.24, 58.10 52.73, 52.60, 39.74, 37.18, 36.12, 32.34, 32.32, 31.98, 31.93, 31.55, 31.40, 31.34, 31.01, 30.90, 30.55, 30.27, 30.19, 30.10, 30.04, 29.92, 29.77, 29.73, 29.69, 29.66, 29.62, 29.56, 28.24, 28.22, 28.17, 28.13, 27.99, 27.58, 27.55, 27.32, 26.23, 25.91, 15.44, 15.43, 15.41, 15.39, 15.38, 15.30. HRMS (ESI-MS) *m*/*z* calculated for $C_{164}H_{248}N_8O_{44}$ $[M+2H]^{2+}$:1517.8780; found: 1517.8787.

Spectroscopic Characterizations of Dendritic Pillar[6]Arenes *pS*/*pR*-11/12/13

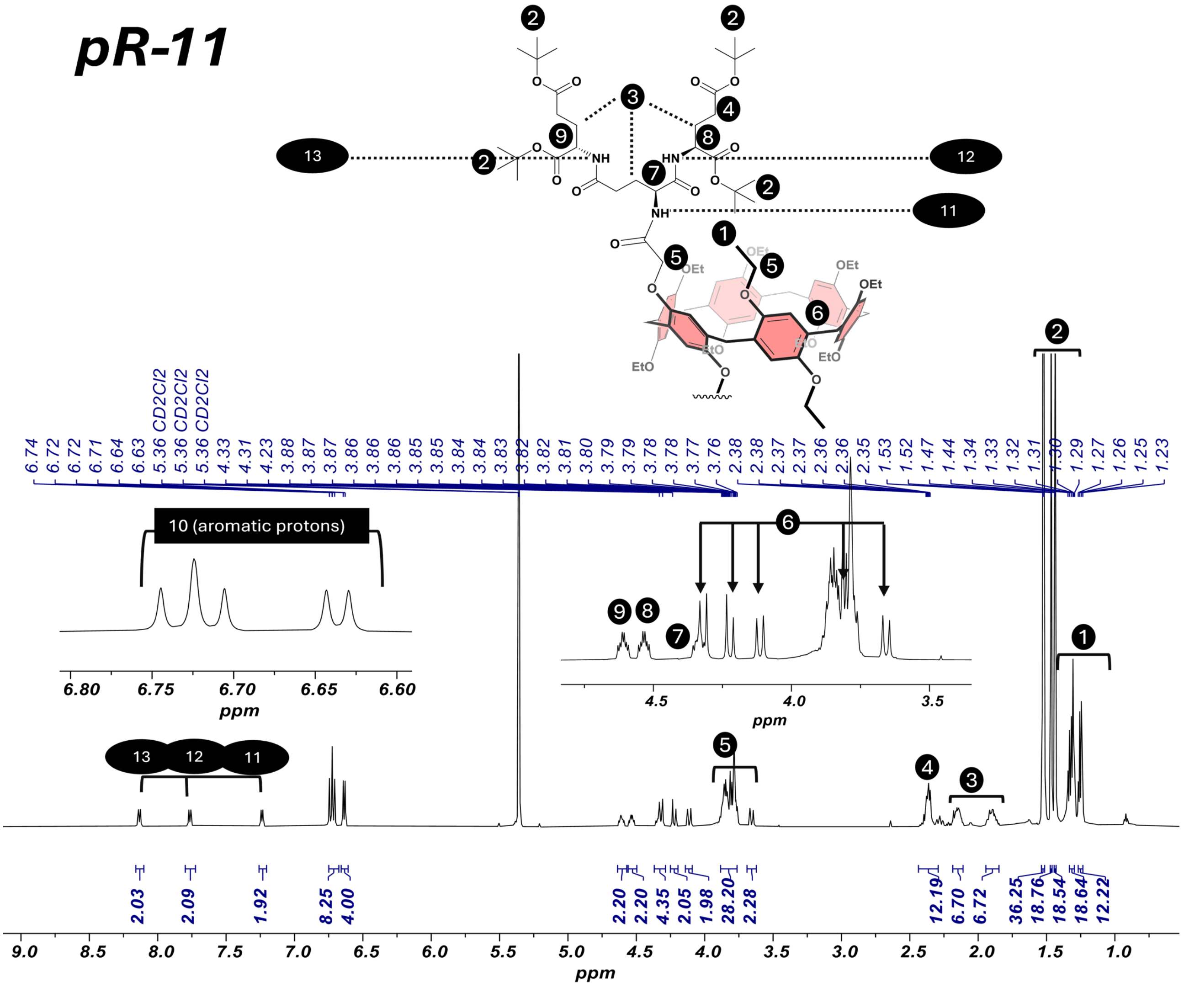

Figure S37. ^{1}H NMR spectrum (600 MHz, 298 K) of dendritic pillar[6]arene *pR*-**11** in CD_2Cl_2.

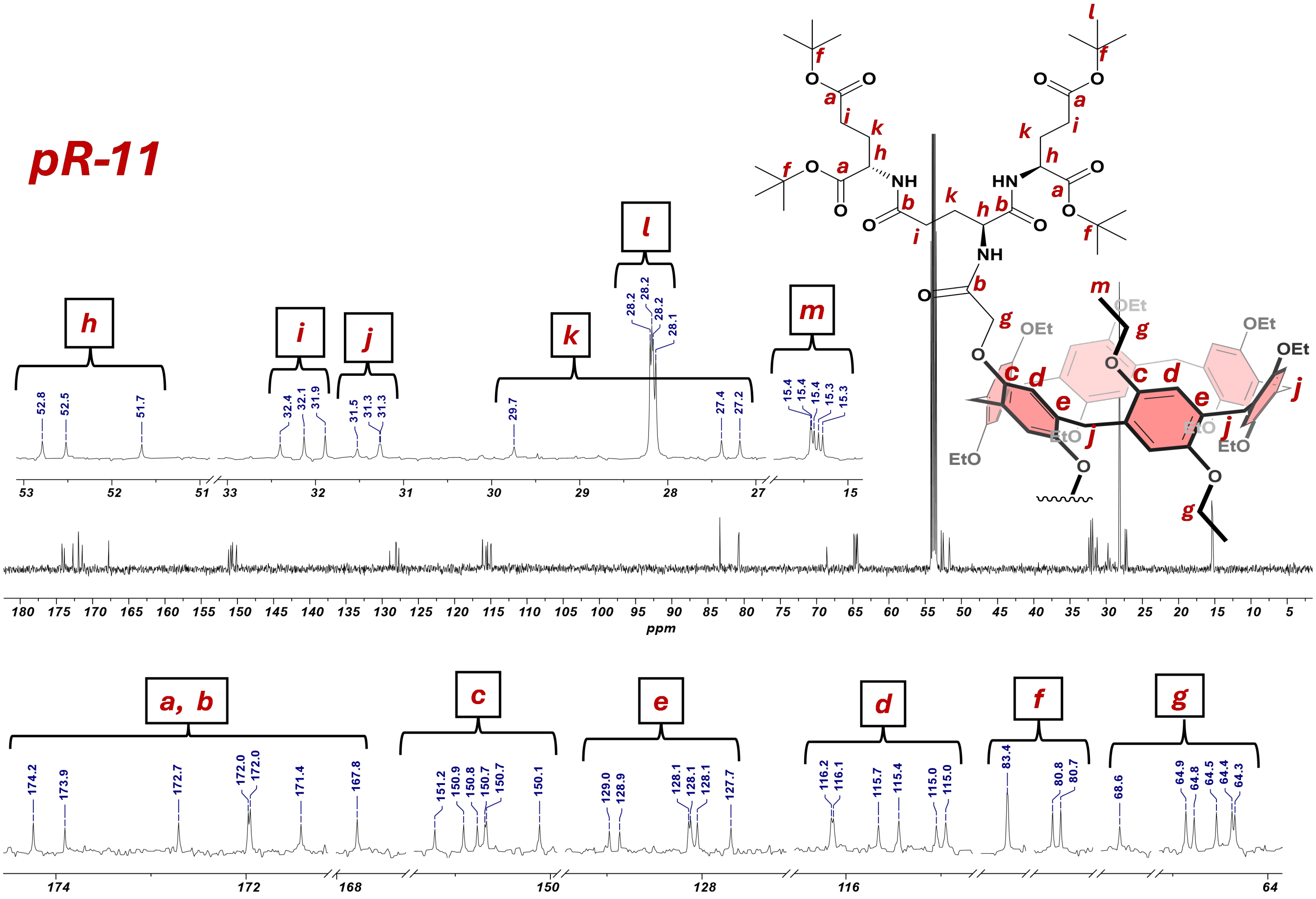

Figure S38. ^{13}C NMR spectrum (150 MHz, 298 K) of dendritic pillar[6]arene *pR*-**11** in CD_2Cl_2.

*p*S-11

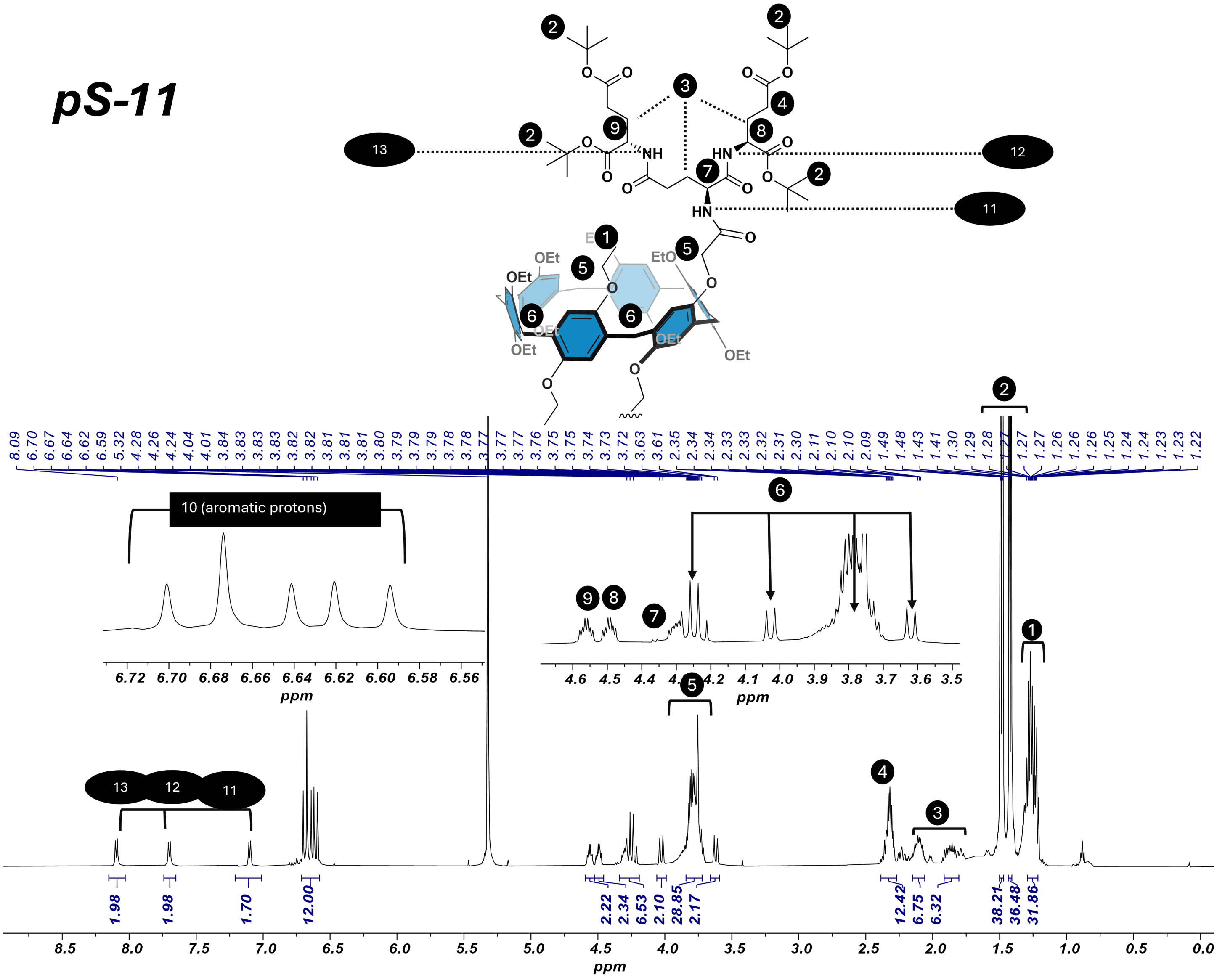

Figure S39. ^{1}H NMR spectrum (600 MHz, 298 K) of dendritic pillar[6]arene *p*S-**11** in CD_2Cl_2.

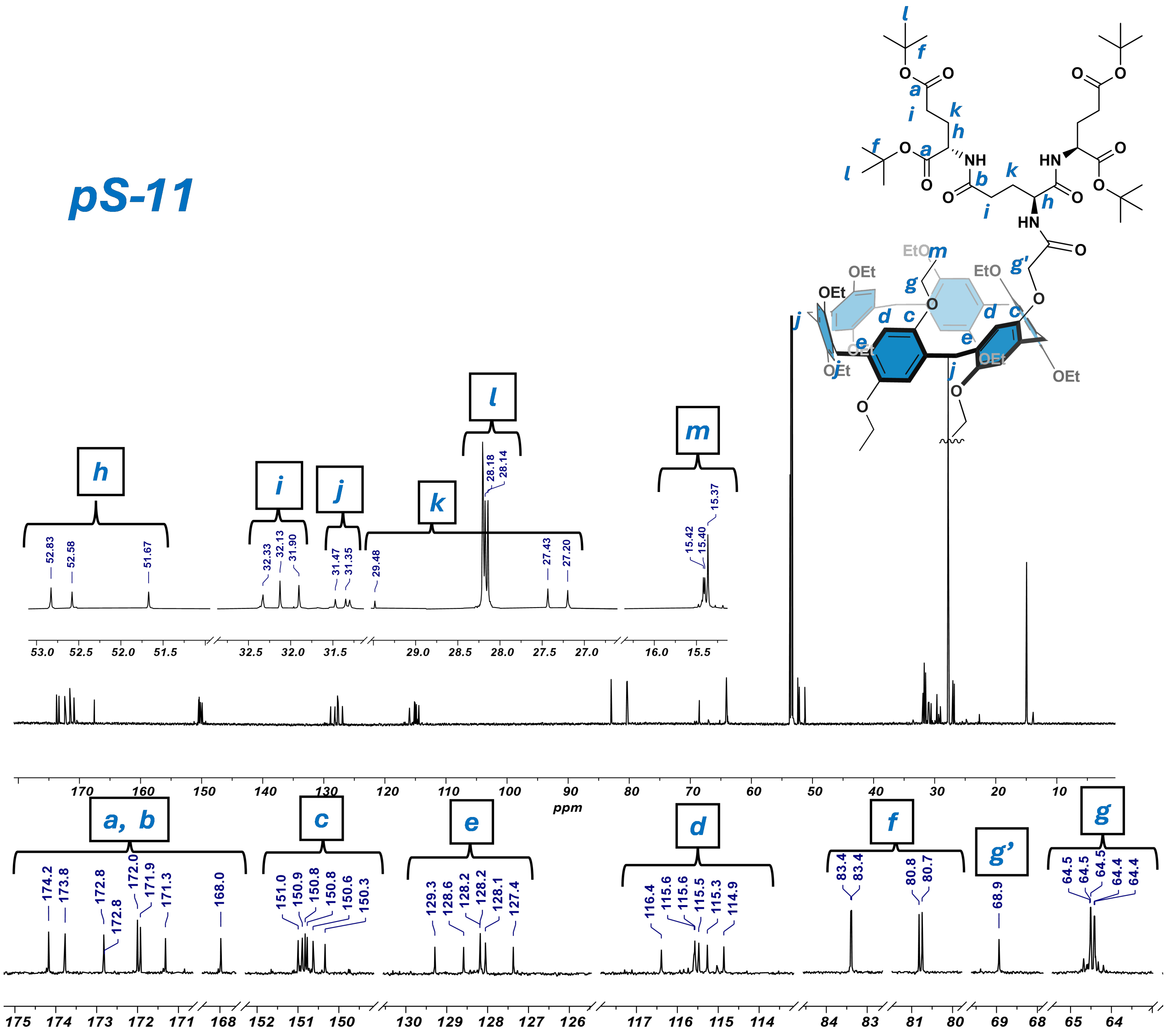

Figure S40. ${}^{13}C$ NMR spectrum (150 MHz, 298 K) of dendritic pillar[6]arene *pS*-**11** in CD_2Cl_2.

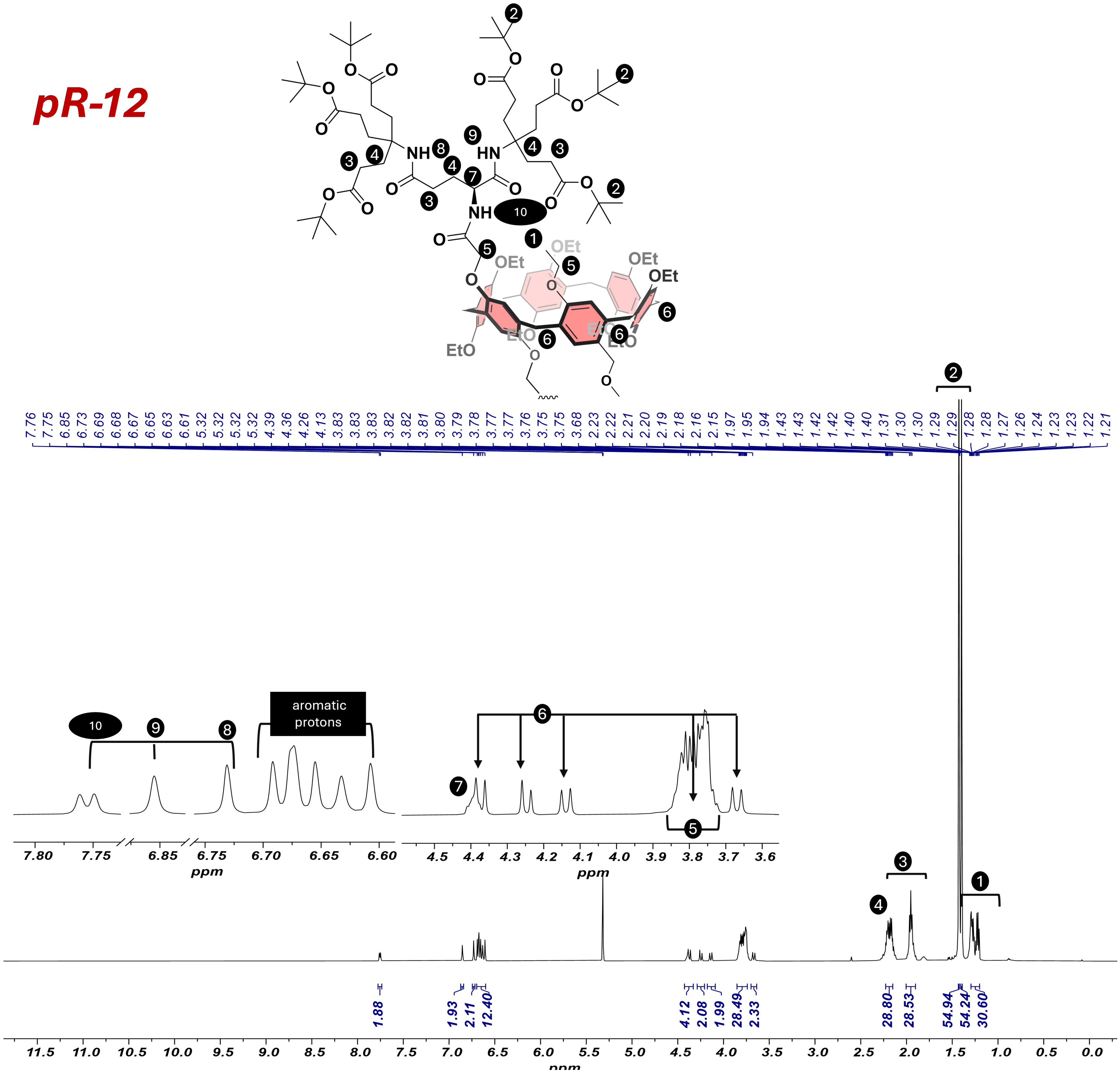

Figure S41. ^{1}H NMR spectrum (600 MHz, 298 K) of dendritic pillar[6]arene *pR*-**12** in CD_2Cl_2.

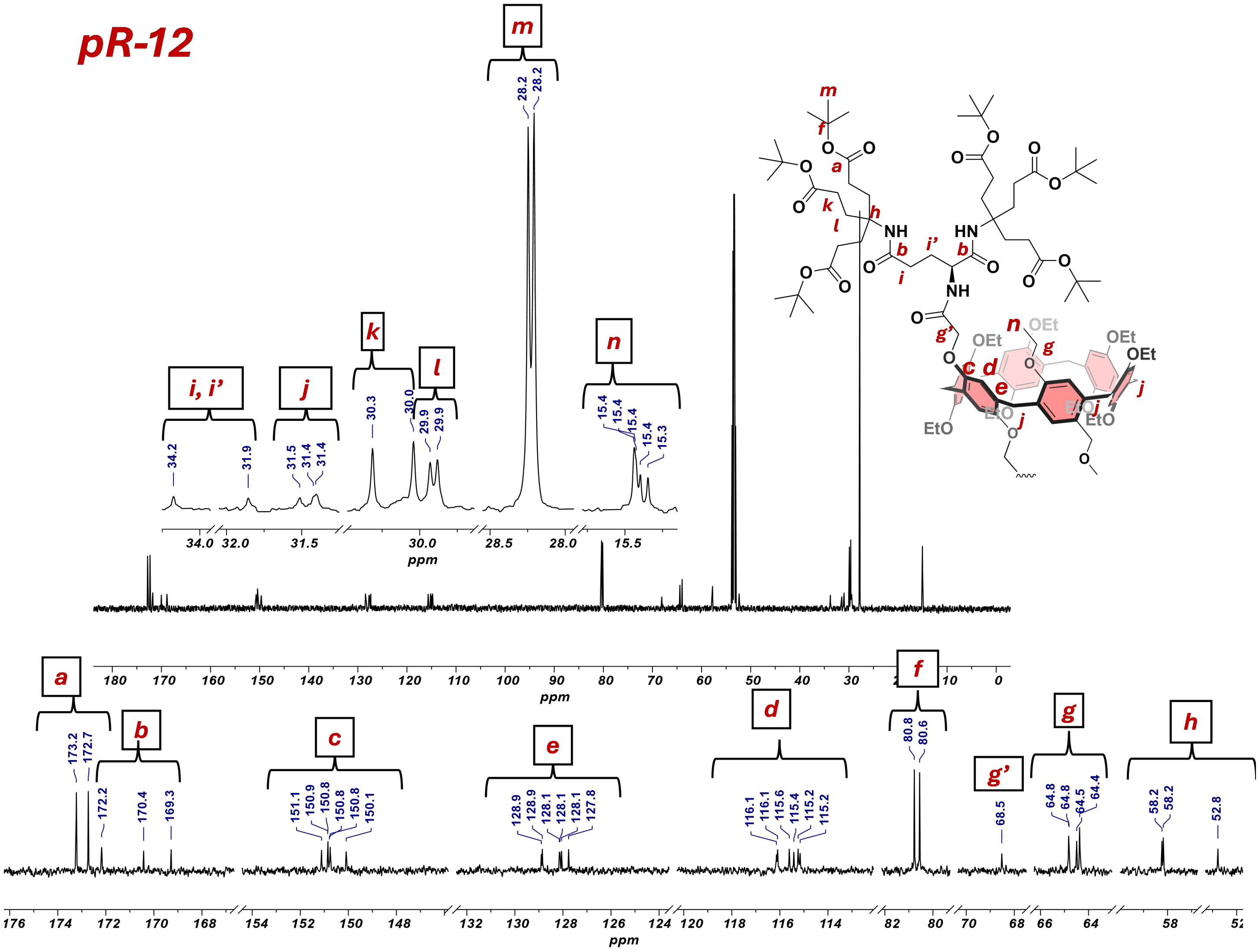

Figure S42. ^{13}C NMR spectrum (150 MHz, 298 K) of dendritic pillar[6]arene *pR*-**12** in CD_2Cl_2.

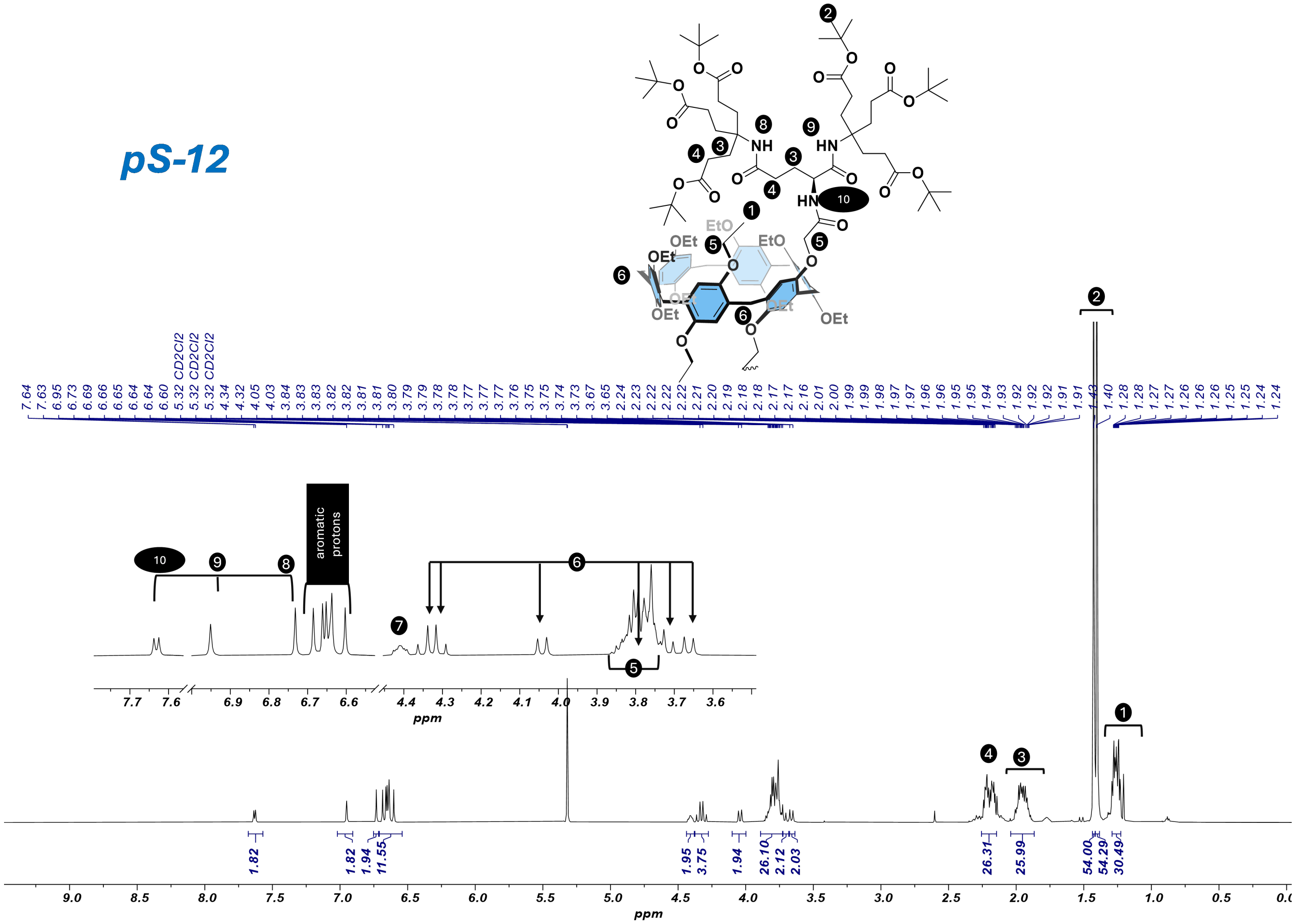

Figure S43. ^{1}H NMR spectrum (600 MHz, 298 K) of dendritic pillar[6]arene *pS*-**12** in CD_2Cl_2.

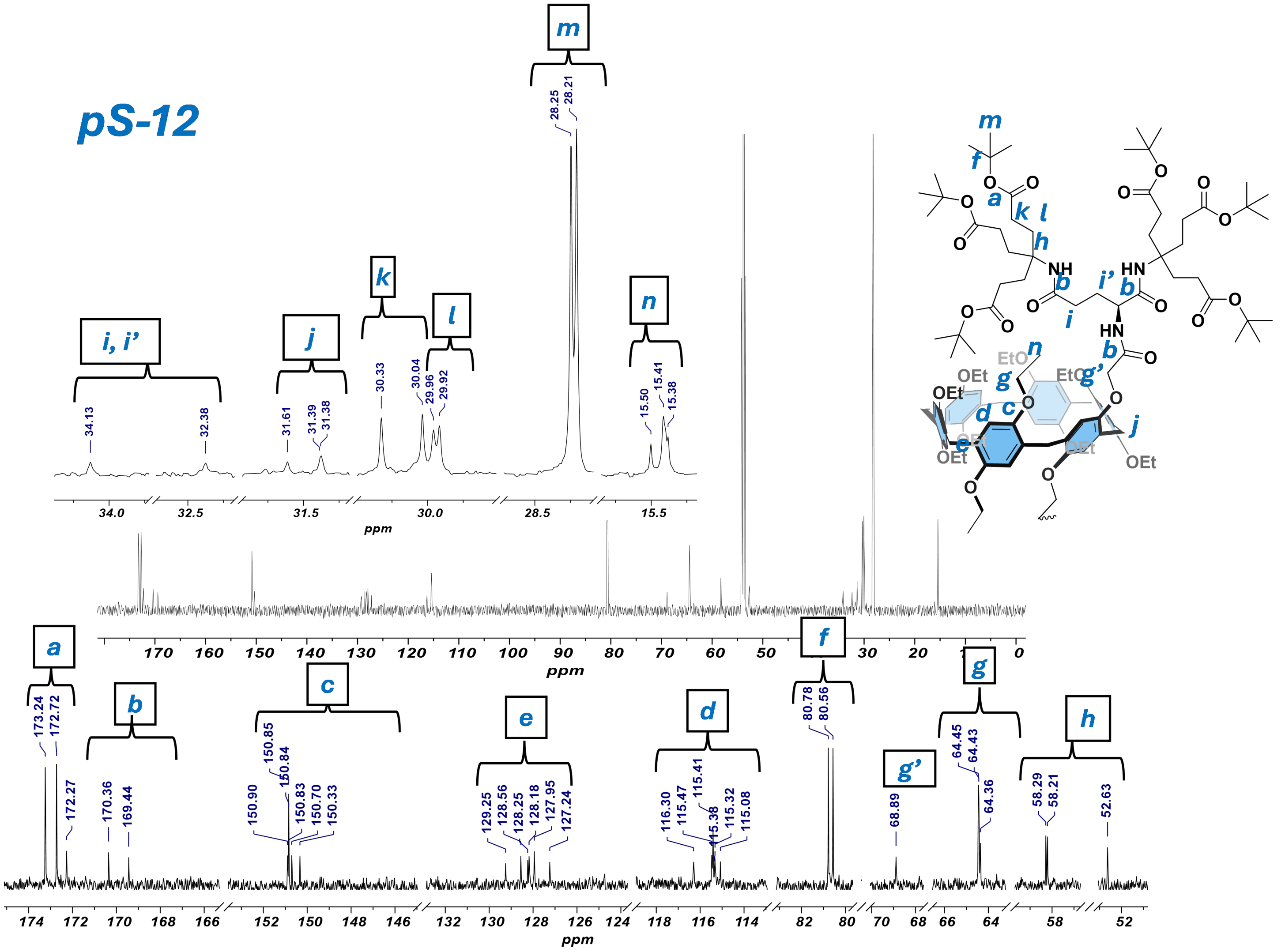

Figure S44. ^{13}C NMR spectrum (150 MHz, 298 K) of dendritic pillar[6]arene *pS*-**12** in CD_2Cl_2.

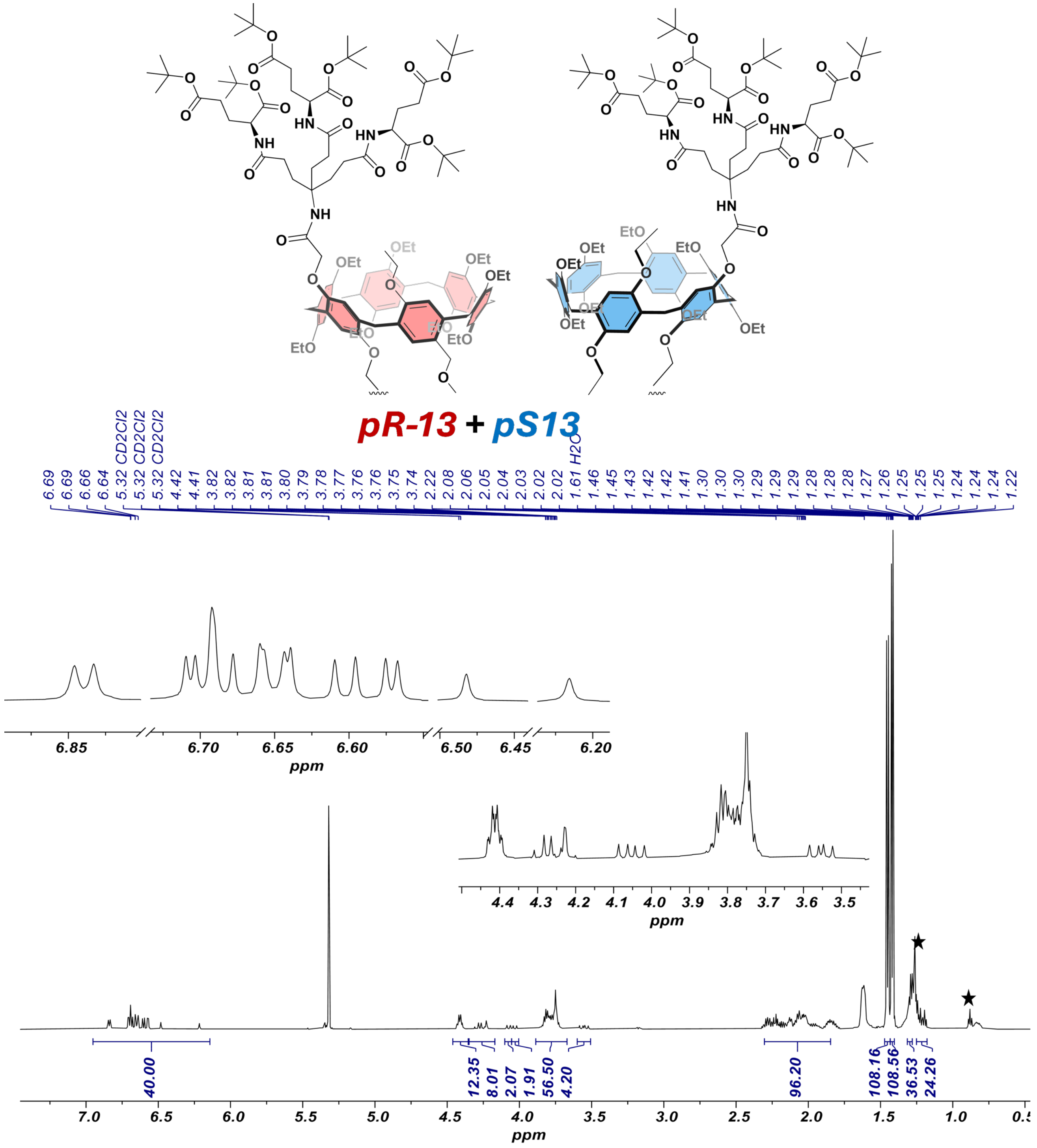

Figure S45. ^{1}H NMR spectrum (600 MHz, 298 K) of dendritic pillar[6]arenes *pS*/*pR*-**13** in CD_2Cl_2; note that grease and hexane impurities are labeled with stars.

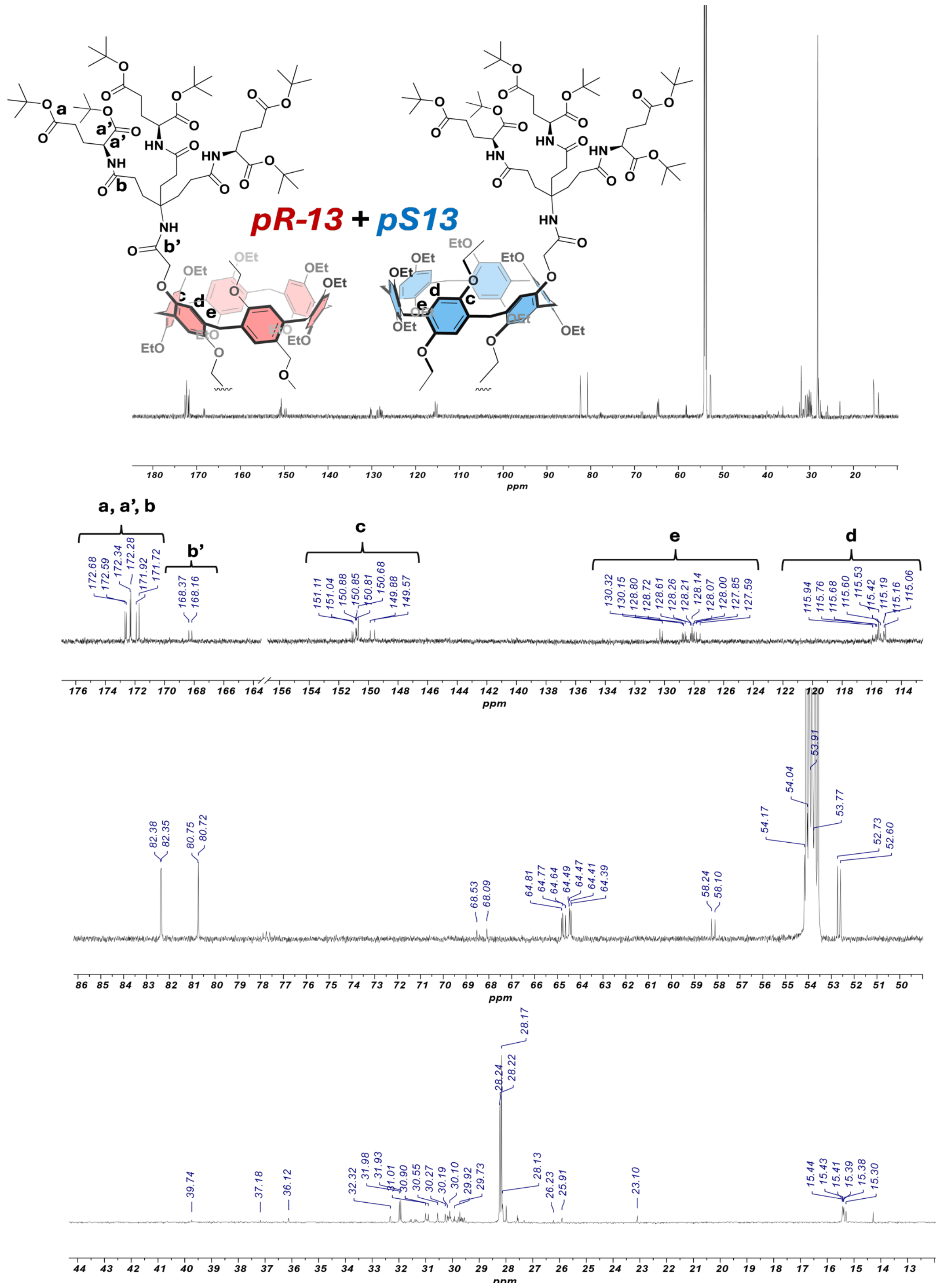

Figure S46. ^{13}C NMR spectrum (600 MHz, 298 K) of dendritic pillar[6]arenes *pS*/*pR*-**13** in CD_2Cl_2.

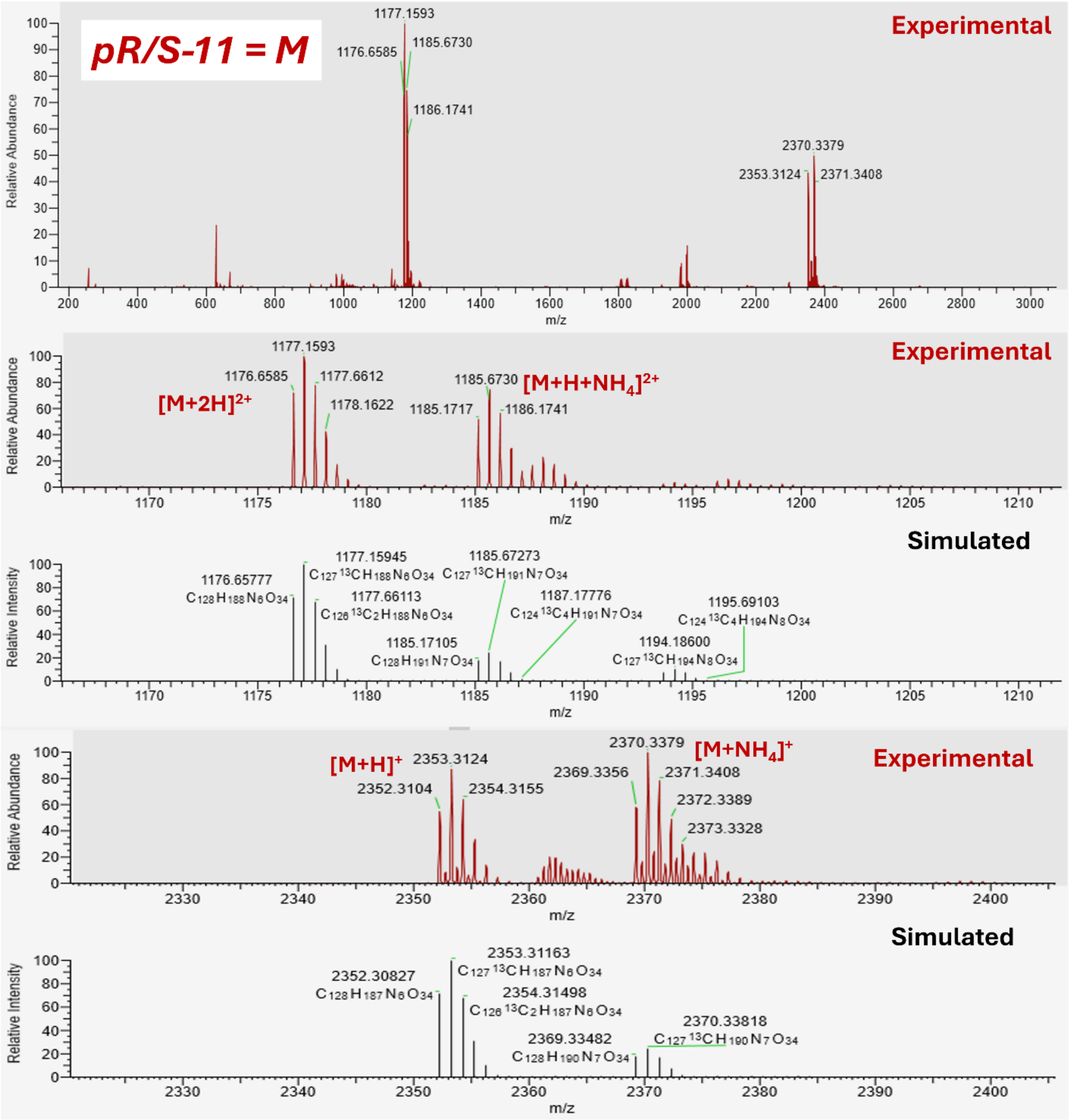
pR/S-11 = M
Experimental
1177.1593
1176.6585
1185.6730
1186.1741
2370.3379
2353.3124
2371.3408
Relative Abundance
m/z
[M+2H]2+
[M+H+NH4]2+
1177.6612
1178.1622
1185.1717
Simulated
Relative Intensity
1176.65777
C128H188N8O34
1177.15945
C127 13C H188N8O34
1177.66113
C126 13C2 H188N8O34
1185.67273
C127 13C H191N7O34
1185.17105
C128H191N7O34
1187.17776
C124 13C4 H191N7O34
1195.69103
C124 13C4 H194N8O34
1194.18600
C127 13C H194N8O34
[M+H]+
[M+NH4]+
2352.3104
2354.3155
2369.3356
2372.3389
2373.3328
2352.30827
C128H187N8O34
2353.31163
C127 13C H187N8O34
2354.31498
C126 13C2 H187N8O34
2369.33482
C128H190N7O34
2370.33818
C127 13C H190N7O34

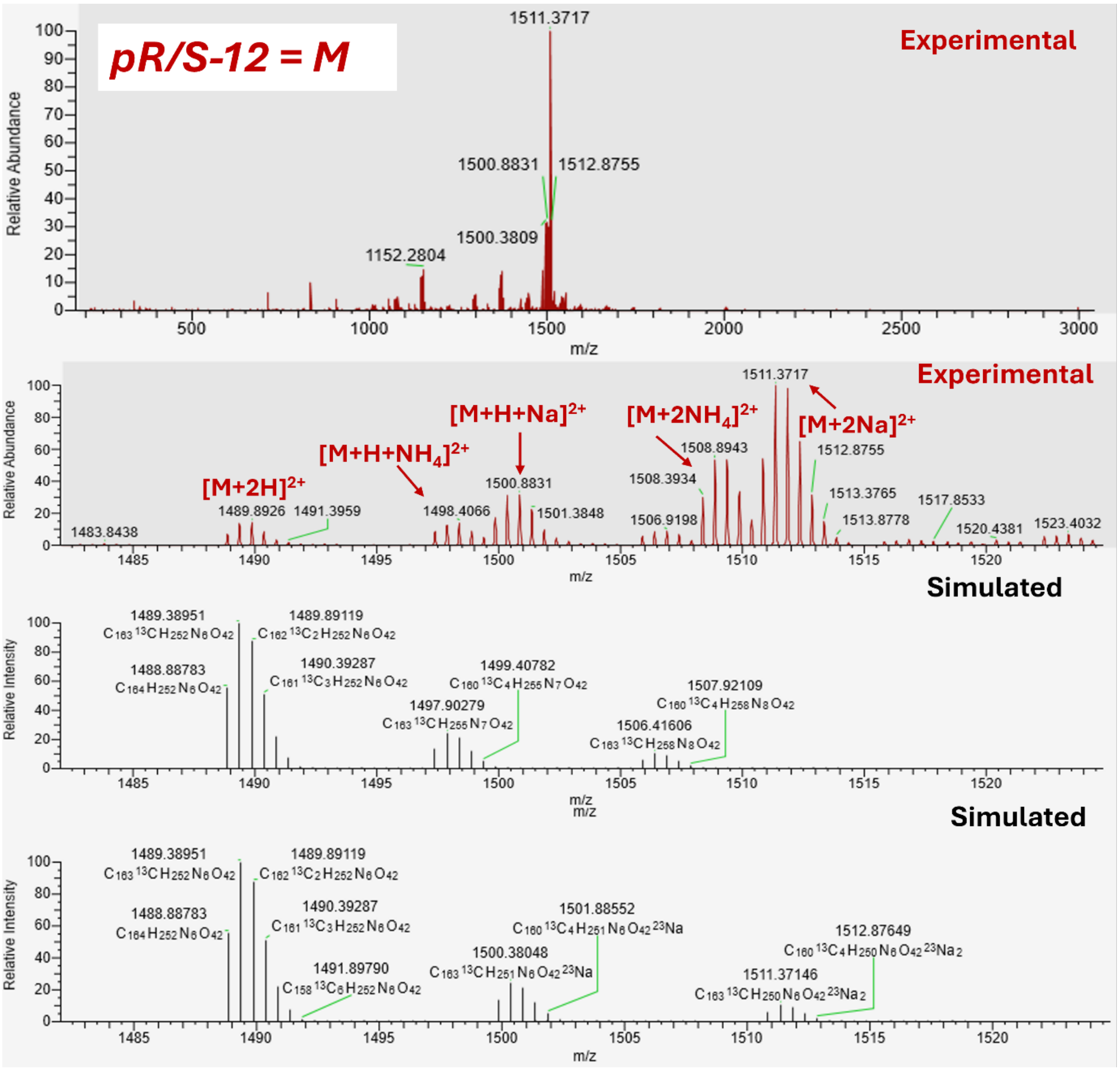

pR/S-12 = M
Experimental
Relative Abundance
m/z
1511.3717
1500.8831
1512.8755
1500.3809
1152.2804
[M+2H]2+
[M+H+NH4]2+
[M+H+Na]2+
[M+2NH4]2+
[M+2Na]2+
1483.8438
1489.8926
1491.3959
1498.4066
1501.3848
1506.9198
1508.3934
1508.8943
1513.3765
1513.8778
1517.8533
1520.4381
1523.4032
Simulated
Relative Intensity
1489.38951
1489.89119
1488.88783
1490.39287
1499.40782
1497.90279
1507.92109
1506.41606
1491.89790
1501.88552
1500.38048
1512.87649
1511.37146

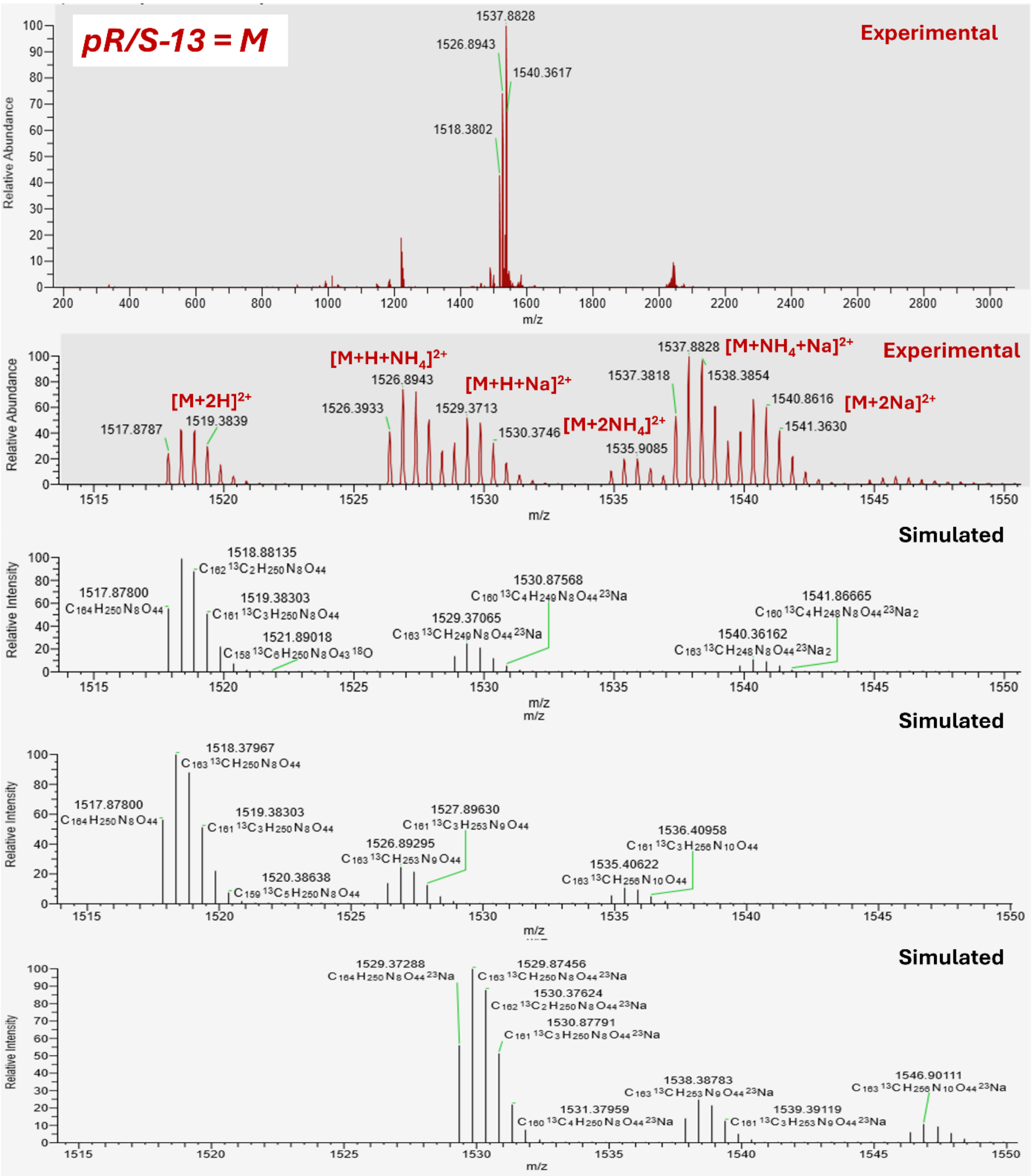

Figure S47. Experimental (red) and simulated (black) mass spectrometry (ESI-MS) signals (with isotope distribution) of the observed ions from *pR*/*pS*-**11**(top left), *pR*/*pS*-**12** (top right) and *pR*/*pS*-**13** (bottom). Full mass spectrometry data are shown for *pR*/*pS*-**11**, *pR*/*pS*-**12** and *pR*/*pS*-**13**.

Diffusion (DOSY) NMR Analysis of Pillar[6]arenes

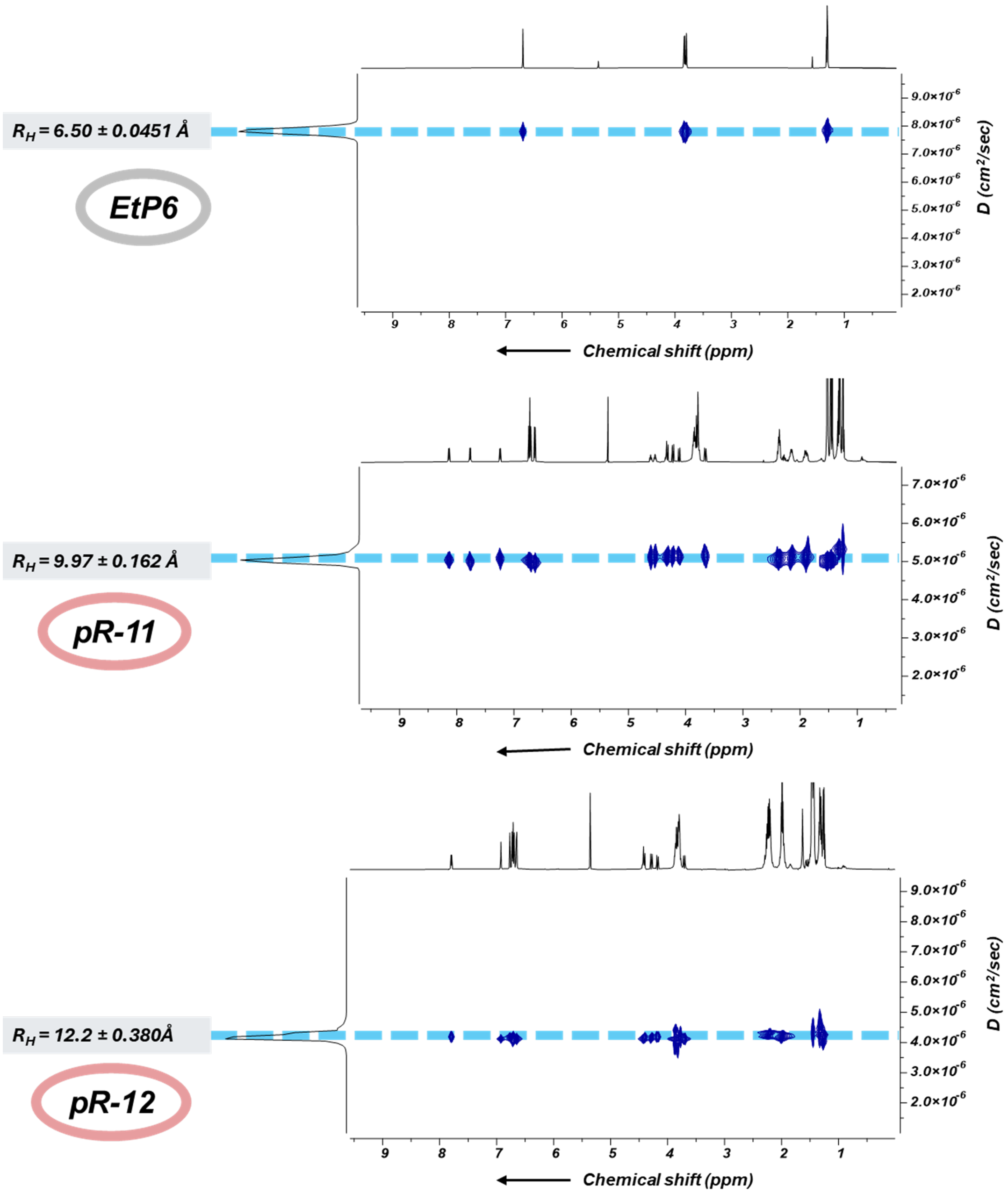

Figure S48. DOSY NMR spectra (600 MHz, 298.0 K) of **EtP6** (1.5 mM), *pR-**11*** (1.5 mM) and *pR-**12*** (1.5 mM) in CD_2Cl_2. Change in intensities of individual peaks over the magnetic field gradient strength were fit to Stejskal-Tanner equation to obtain diffusion coefficients corresponding to each peak using Mnova NMR processing software by Bruker (Tables S1-S3, below). Hydrodynamic radii for **EtP6**, *pR-**11*** and *pR-**12*** were $R_{H} =$ 6.50 ± 0.04 Å, 10.0 ± 0.2 Å and 12.2 ± 0.4 (mean ± standard deviation), respectively. These values were calculated using the Stokes-Einstein equation for which the viscosity of CD_2Cl_2 and at 298.0 K was taken as η = 0.43 cP. Solvent peaks were excluded from the fit.

Chemical Shift (ppm)	D(cm^2/sec)	Error (cm2/sec)	R_H(m)
6.70	7.82E-06	9.08E-08	6.49E-10
3.85	7.71E-06	1.09E-07	6.58E-10
3.84	7.85E-06	9.73E-08	6.46E-10
3.83	7.84E-06	9.35E-08	6.48E-10
3.81	7.75E-06	1.25E-07	6.55E-10
3.80	7.81E-06	9.24E-08	6.50E-10
3.81	7.75E-06	1.25E-07	6.55E-10
1.32	7.81E-06	1.11E-07	6.50E-10
1.29	7.86E-06	1.17E-07	6.46E-10
1.30	7.87E-06	9.58E-08	6.45E-10
		Average =	6.50E-10
		Std Dev =	4.51E-12

Table S1. Diffusion coefficients and hydrodynamic radii corresponding to DOSY NMR signals from **EtP6** pillar[6]arene.

Chemical Shift (ppm)	D(cm²/sec)	Error (cm2/sec)	R_H(m)
8.14	5.06E-06	6.56E-08	1.00E-09
8.13	5.02E-06	6.46E-08	1.01E-09
7.77	5.02E-06	6.74E-08	1.01E-09
7.76	4.99E-06	6.47E-08	1.02E-09
7.25	5.09E-06	6.62E-08	9.97E-10
7.24	5.02E-06	7.46E-08	1.01E-09
6.74	5.05E-06	5.46E-08	1.00E-09
6.72	5.02E-06	4.45E-08	1.01E-09
6.70	4.98E-06	6.86E-08	1.02E-09
6.64	4.98E-06	4.70E-08	1.02E-09
6.63	4.97E-06	6.53E-08	1.02E-09
4.61	5.09E-06	1.00E-07	9.97E-10
4.61	5.08E-06	1.07E-07	9.99E-10
4.54	5.09E-06	8.63E-08	9.96E-10
4.53	5.17E-06	9.45E-08	9.81E-10
4.33	5.13E-06	6.65E-08	9.89E-10
4.31	5.17E-06	6.80E-08	9.82E-10
4.24	5.10E-06	6.78E-08	9.95E-10
4.21	5.18E-06	6.30E-08	9.80E-10
4.13	5.17E-06	6.95E-08	9.82E-10
4.10	5.08E-06	6.09E-08	9.99E-10
3.67	5.15E-06	9.27E-08	9.86E-10
3.64	5.12E-06	9.71E-08	9.92E-10
2.40	5.10E-06	8.67E-08	9.96E-10
2.38	4.98E-06	4.64E-08	1.02E-09
2.37	5.05E-06	4.69E-08	1.01E-09
2.36	5.08E-06	5.11E-08	1.00E-09
2.35	5.10E-06	5.60E-08	9.95E-10
2.17	5.03E-06	8.64E-08	1.01E-09
2.16	5.07E-06	6.33E-08	1.00E-09
2.15	5.10E-06	6.89E-08	9.96E-10
2.14	5.16E-06	6.56E-08	9.84E-10
2.14	5.15E-06	7.57E-08	9.85E-10
1.92	5.09E-06	6.61E-08	9.98E-10
1.90	5.00E-06	7.01E-08	1.02E-09
1.89	5.12E-06	8.03E-08	9.91E-10
1.88	5.19E-06	9.19E-08	9.77E-10
1.86	5.30E-06	1.20E-07	9.58E-10
1.53	5.00E-06	4.78E-08	1.02E-09
1.52	5.07E-06	5.59E-08	1.00E-09
1.47	5.05E-06	5.54E-08	1.01E-09
1.44	5.04E-06	5.48E-08	1.01E-09
1.32	5.33E-06	7.29E-08	9.53E-10
1.25	5.30E-06	2.42E-07	9.58E-10
	=average(	Average	9.97E-10
		Std Dev	1.61629E-11

Table S2. Diffusion coefficients and hydrodynamic radii corresponding to DOSY NMR signals from *pR*-**11** pillar[6]arene.

Chemical shift(ppm)	D(cm^2/sec)	Error (cm2/sec)	R_H(m)
7.80	4.17E-06	5.80E-08	1.22E-09
7.79	4.16E-06	5.77E-08	1.22E-09
6.93	4.10E-06	3.14E-08	1.24E-09
6.77	4.16E-06	3.23E-08	1.22E-09
6.73	4.12E-06	3.70E-08	1.23E-09
6.71	4.05E-06	5.45E-08	1.25E-09
6.71	4.18E-06	4.79E-08	1.22E-09
6.69	4.10E-06	4.38E-08	1.24E-09
6.66	4.11E-06	3.65E-08	1.24E-09
6.65	4.14E-06	5.21E-08	1.23E-09
4.42	4.11E-06	3.93E-08	1.23E-09
4.40	4.17E-06	5.03E-08	1.22E-09
4.30	4.12E-06	4.18E-08	1.23E-09
4.27	4.20E-06	4.85E-08	1.21E-09
4.19	4.18E-06	5.81E-08	1.21E-09
4.16	4.19E-06	5.81E-08	1.21E-09
3.88	3.96E-06	1.27E-07	1.28E-09
3.87	3.96E-06	1.20E-07	1.28E-09
3.86	3.96E-06	5.62E-08	1.28E-09
3.86	4.40E-06	6.31E-08	1.15E-09
3.85	4.14E-06	2.52E-08	1.23E-09
3.84	4.09E-06	2.40E-08	1.24E-09
3.83	3.92E-06	1.70E-07	1.30E-09
3.81	4.13E-06	2.73E-08	1.23E-09
3.81	4.12E-06	2.61E-08	1.23E-09
3.80	4.17E-06	2.76E-08	1.22E-09
3.80	4.10E-06	2.09E-08	1.24E-09
3.79	4.15E-06	2.94E-08	1.22E-09
3.79	4.18E-06	5.49E-08	1.21E-09
3.77	4.22E-06	1.26E-07	1.20E-09
3.72	4.14E-06	5.87E-08	1.22E-09
3.69	4.09E-06	5.75E-08	1.24E-09
2.24	4.20E-06	1.84E-08	1.21E-09
2.23	4.29E-06	2.08E-08	1.18E-09
2.21	4.32E-06	2.26E-08	1.18E-09
2.20	4.32E-06	2.48E-08	1.18E-09
1.99	4.19E-06	1.56E-08	1.21E-09
1.98	4.27E-06	3.00E-08	1.19E-09
1.45	4.33E-06	1.69E-07	1.17E-09
1.33	4.47E-06	2.78E-07	1.14E-09
1.33	4.28E-06	4.73E-08	1.19E-09
1.32	4.30E-06	5.00E-08	1.18E-09
1.32	4.59E-06	1.13E-07	1.11E-09
1.31	4.47E-06	6.33E-08	1.14E-09
1.27	4.06E-06	8.49E-08	1.25E-09
1.27	4.06E-06	8.49E-08	1.25E-09
1.26	4.23E-06	4.96E-08	1.20E-09
		Avrage	1.22E-09
		Std Dev	3.79664E-11

Table S3. Diffusion coefficients and hydrodynamic radii corresponding to DOSY NMR signals from *pR*-**12** pillar[6]arene.

Variable temperature ^{1}H NMR Spectroscopic Studies of Pillar[6]Arenes

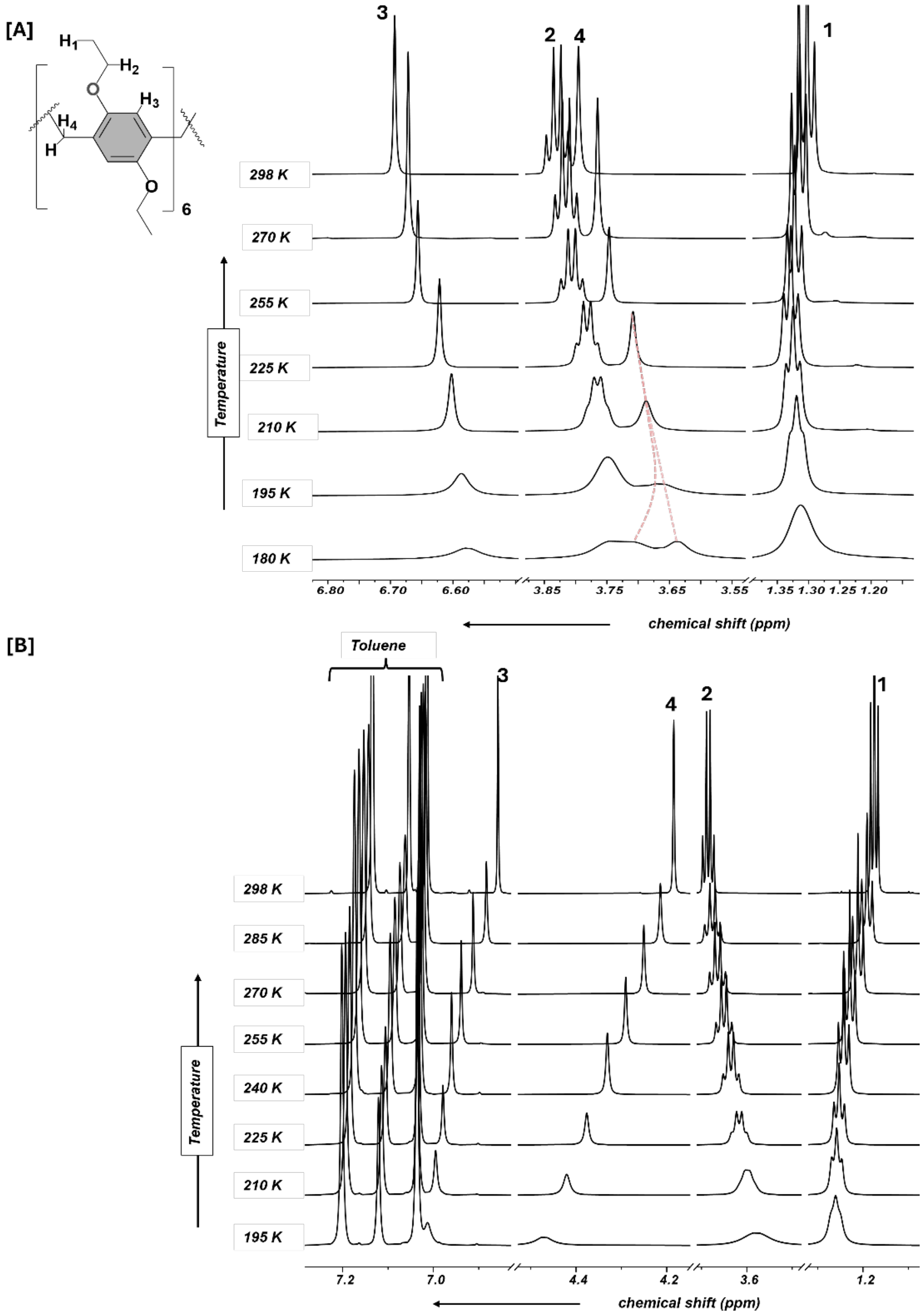

Figure S49. Variable temperature ^{1}H NMR spectra (600 MHz) of pillar[6]arene ***EtP6*** in (A) CD_2Cl_2 and (B) toluene-d_8. Note that signals from diastereotopic CH_2 protons labeled as 2 and 4 underwent a greater degree of broadening than others.

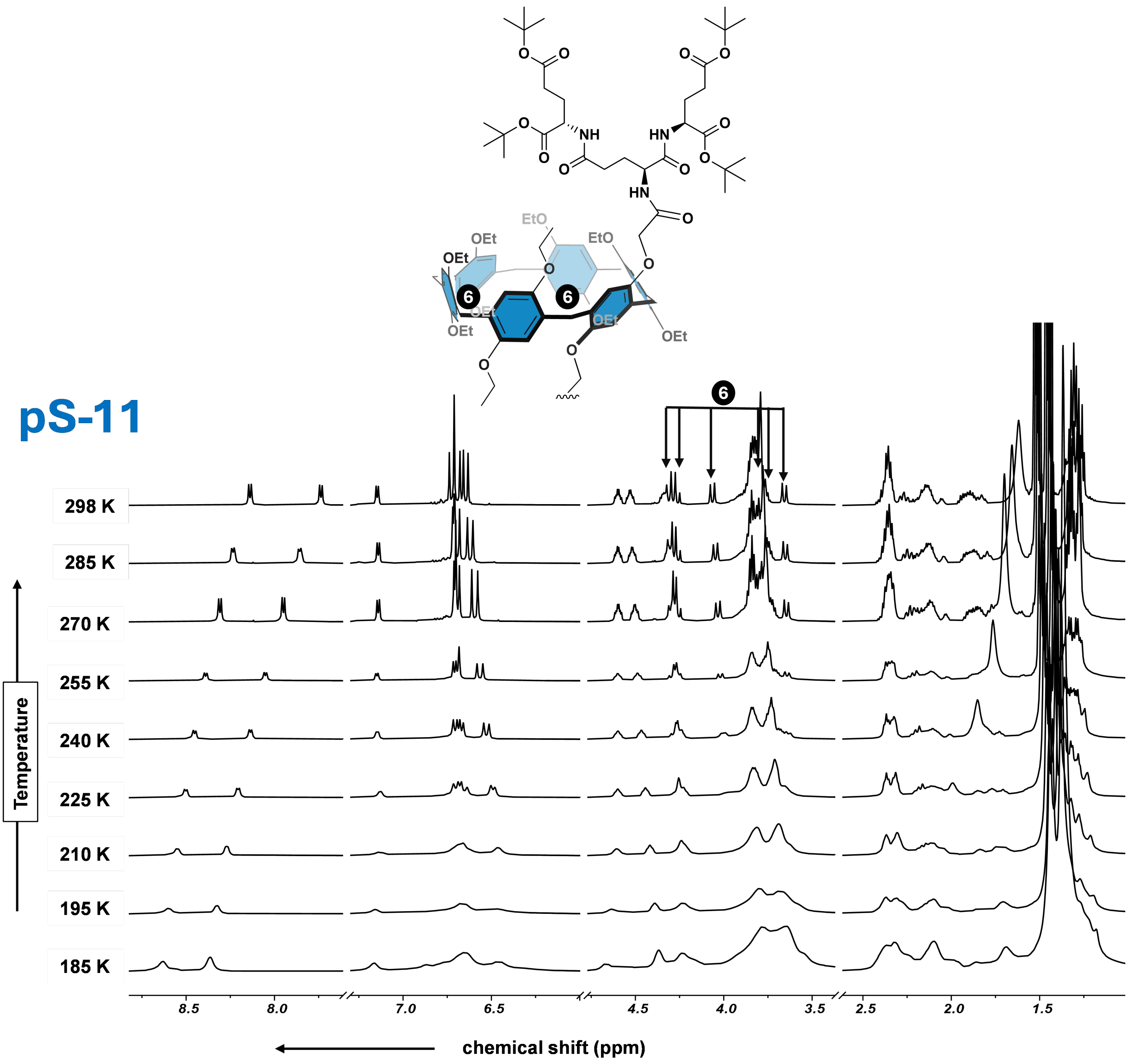

Figure S50. Variable temperature ^{1}H NMR spectra (600 MHz) of dendritic pillar[6]arene *pS*-**11** in CD_2Cl_2.

Synthesis and Characterization of Dendritic Pillar[6]Arenes *pS*/*pR*-11^{8-} and *pS*/*pR*-11^{8-}

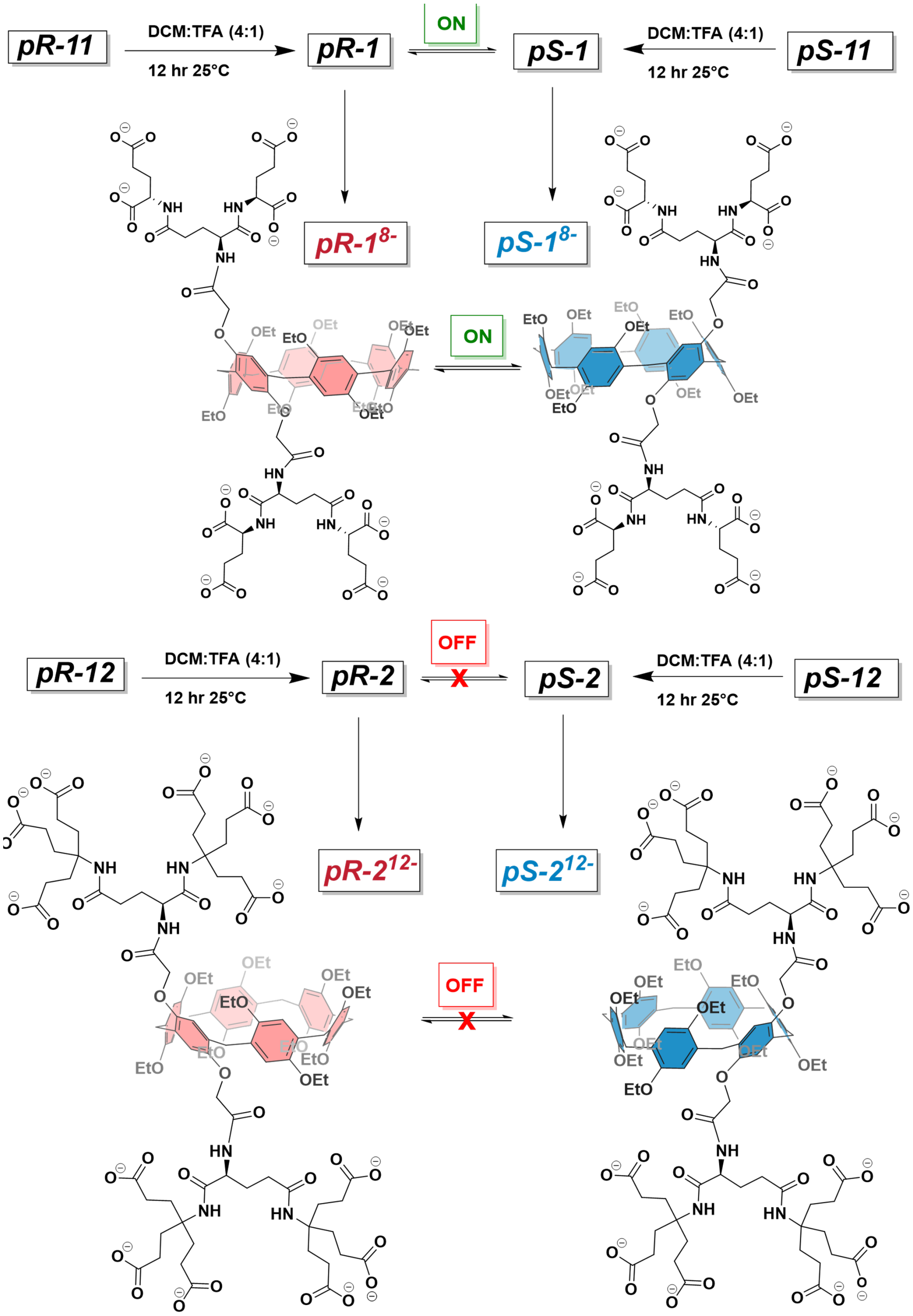

Scheme S4. Synthetic scheme describing the preparation of dendritic pillar[6]arenes *pR/pS*-**1**$^{8-}$ and *pR/pS*-**2**$^{12-}$.

Dendritic pillar[6]arenes *pR*-1^{8-} and *pS*-1^{8-}. Pillar[6]arene *pR*-**11** (10 mg) was dissolved in 2 mL of dichloromethane: trifluoroacetic acid = 8:1 and the reaction mixture was stirred at room temperature for 12 h. The solvent was evaporated under reduced pressure and the remaining solid suspended in 1 mL of distilled water. 1N HCl was slowly added to the suspension to bring the pH of such aqueous solution between 1 and 2. The precipitate was isolated via centrifugation, and the purification procedure repeated. After lyophilization and dissolution of the expected product *pR*-**1** (7.93 mg, 98%) in 30 mM phosphate buffer at pH = 7.0 (Figure S51), the formation of diastereomeric *pR*-**1**$^{8-}$/*pS*-**1**$^{8-}$ mixture ensued (Figure S51); when the alleged product *pR*-**1** was dissolved in DMSO-d^6, the formation of *pR*-**1** and *pS*-**1** was observed (Figure S52). HRMS (ESI-MS) *m*/*z* calculated for $C_{96}H_{122}N_6O_{34}$ [M+2H]$^{2+}$: 952.4074; found: 952.4079.

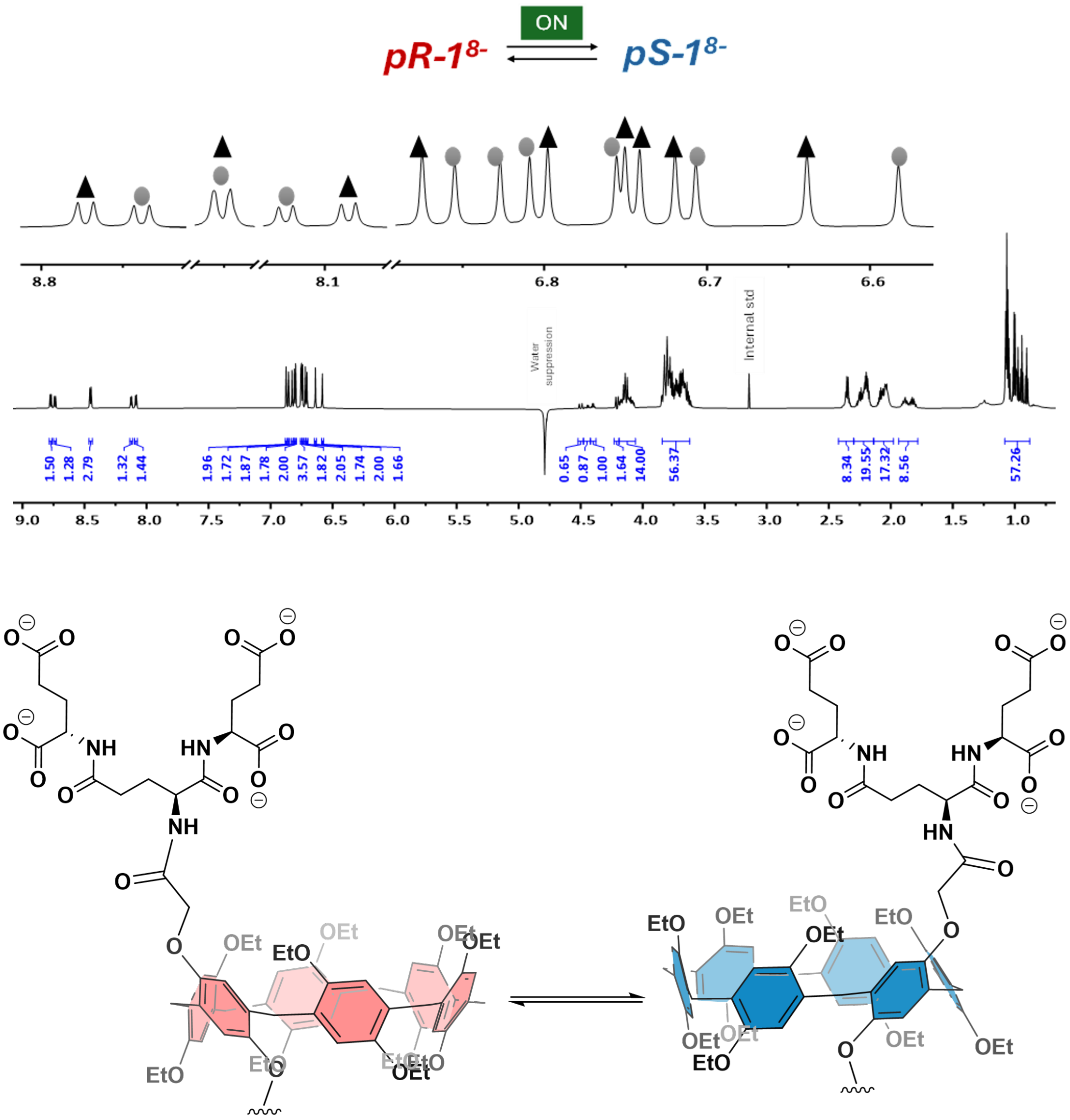

Figure S51. ^{1}H NMR spectrum (600 MHz, 298 K; water suppression) of dendritic pillar[6]arenes *pS*-**1**$^{8-}$ and *pR*-**1**$^{8-}$ in 30 mM phosphate buffer at pH = 7.0 (10% D_2O) was obtained after deprotection of *pR*-**11**.

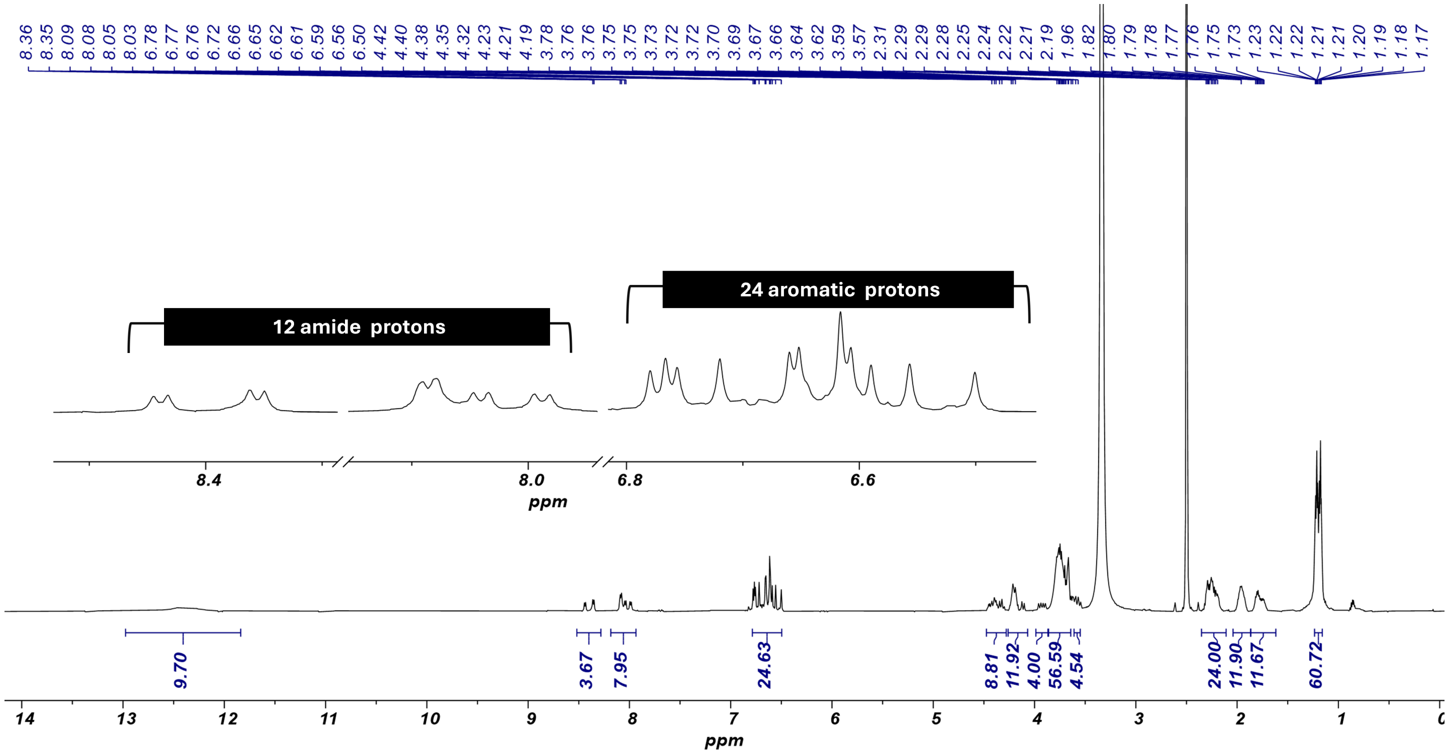

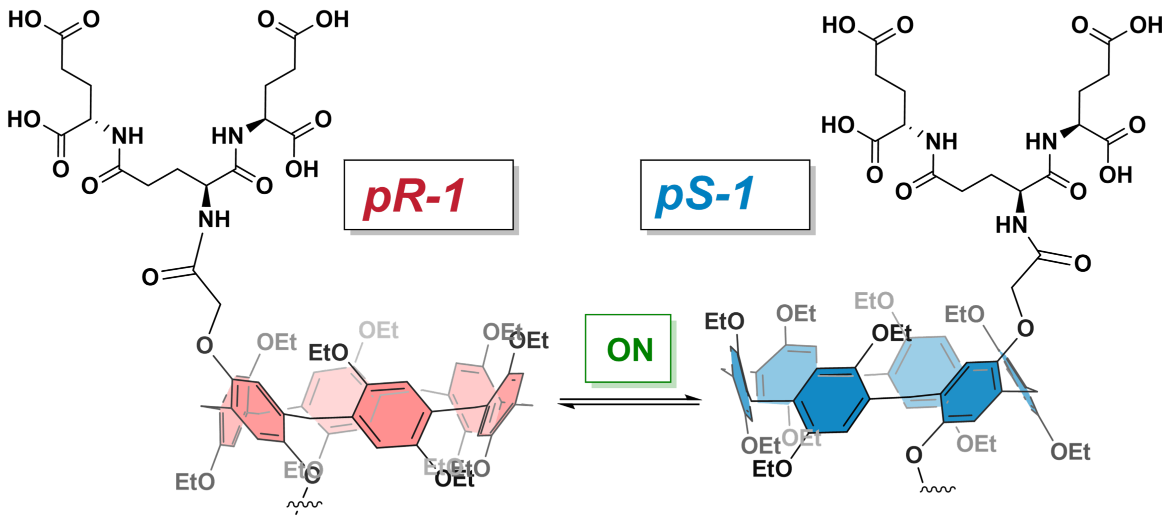

Figure S52.^{1}H NMR spectrum (600 MHz, 298 K; DMSO-d$_6$) of dendritic pillar[6]arenes *pS*-**1** and *pR*-**1** obtained after deprotection of *pR*-**11.**

Dendritic pillar[6]arene *pR*-2. Dendritic pillar[6]arene *pR*-**12** (10 mg) was dissolved in 2 mL of dichloromethane: trifluoroacetic acid = 8:1 and the reaction mixture stirred at room temperature for 12 h. The solvent was evaporated under reduced pressure and the remaining solid was suspended in 1 mL of distilled water. 1N HCl was slowly added to the suspension to bring the pH of to bring the pH of such aqueous solution between 1 and 2. The precipitate was isolated via centrifugation, and the purification procedure repeated. After lyophilization, 7.65 mg (99%) of *pR*-**2** was obtained as an off white solid. ^{1}H NMR (600 MHz, DMSO-d^{6}) δ (ppm) = 12.04 (s, 12H), 7.97 (d, *J* = 7.7 Hz, 2H), 7.42 (s, 2H), 7.17 (s, 2H), 6.79 (s, 2H), 6.73 (s, 2H), 6.65 (s, 2H), 6.62 (s, 2H), 6.60 (s, 2H), 6.54 (s, 2H), 4.16 (d, *J* = 14.1 Hz, 4H), 3.94 (d, *J* = 13.4 Hz, 2H), 3.63 (d, *J* = 14.4 Hz 2H), 3.59 -3.56(m, 30H), 2.25 – 1.98 (m, 28H), 1.83 (m, 28H), 1.57 – 0.73 (m, 30H). ^{13}C NMR (214 MHz, DMSO-d^{6}) δ (ppm) = 174.43, 174.38, 174.36, 171.31, 170.57, 167.55, 149.82, 149.79, 149.73, 127.79, 127.09, 126.96, 126.81, 126.75, 115.79, 114.82, 114.70, 114.65, 114.59, 68.01, 63.52, 63.45, 63.42, 63.40, 56.70, 56.38, 52.50, 39.97, 39.88, 39.81, 39.78, 39.72, 39.62, 39.52, 39.42, 39.32, 39.23, 32.47, 30.96, 30.88, 30.76, 29.12, 29.03, 29.00, 28.04, 27.99, 14.91, 14.89, 14.83. HRMS (ESI-MS) *m*/*z* calculated for C116H154N6O42, [M+2H]$^{2+}$: 1152.5122; found: 1152.5122.

Dendritic pillar[6]arene *pS*-2. By following the protocol described above for deprotection of dendritic pillar[6]arene *pR*-**12**, 10 mg of *pS*-**12** was converted into 7.52 mg (97%) of dendritic pillar[6]arene *pS*–**2** as an off white solid. ^{1}H NMR (600 MHz, DMSO-d^{6}) δ (ppm) = 12.04 (s, 12H), 7.96 (d, *J* = 7.9 Hz, 2H), 7.48 (s, 2H), 7.18 (s, 2H), 6.81 (s, 2H), 6.75 (s, 2H), 6.67 (s, 2H), 6.63 (s, 1H), 6.60 (s, 2H), 6.51 (s, 2H), 4.35 (d, *J* = 13.6 Hz, 4H), 4.15 (d, *J* = 13.7 Hz, 2H), 3.93 (d, *J* = 13.4 Hz, 2H), 3.87 – 3.62 (m, 28H),3.57 (d, *J* = 13.2 Hz, 2H), 2.11 (m, 28H), 1.99 – 1.70 (m, 28H), 1.42 – 1.02 (m, 30H). ^{13}C NMR (214 MHz, DMSO-d^{6}) δ (ppm) = 174.44, 174.35, 171.31, 170.49, 167.37, 149.84, 149.80, 149.78, 149.77, 149.70, 149.68, 127.88, 126.94, 126.92, 126.82, 126.77, 126.68, 115.77, 114.84, 114.72, 114.64, 114.58, 114.36, 67.90, 63.46, 63.40, 63.37, 63.29, 63.27, 56.73, 56.37, 52.31, 32.29, 30.99, 29.14, 29.02, 28.05, 28.00, 14.92, 14.89, 14.88. HRMS (ESI-MS) *m*/*z* calculated for C116H154N6O42, [M+2H]$^{2+}$: 1152.5122; found: 1152.5122.

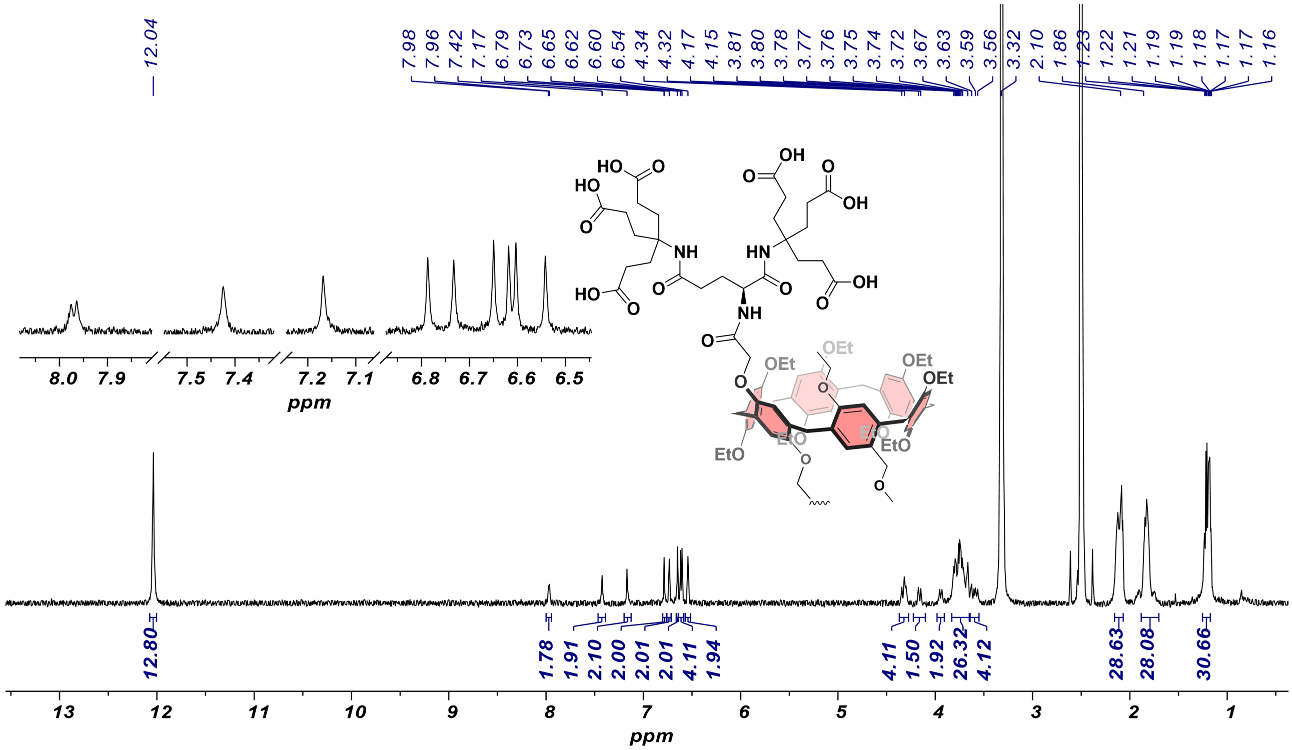

Figure S53. ^{1}H NMR spectrum (600 MHz, 298 K) of dendritic pillar[6]arene *pR*-**2** in DMSO-d_6.

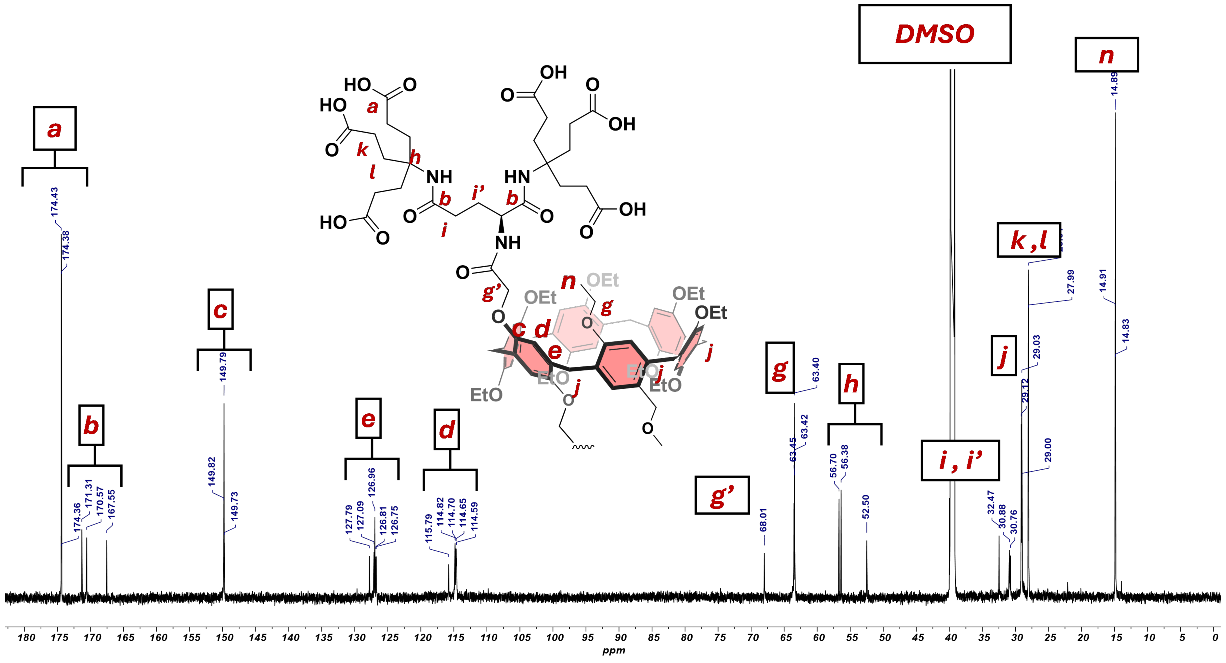

Figure S54. ^{13}C NMR spectrum (200 MHz, 298 K) of dendritic pillar[6]arene *pR*-**2** in DMSO-d_6.

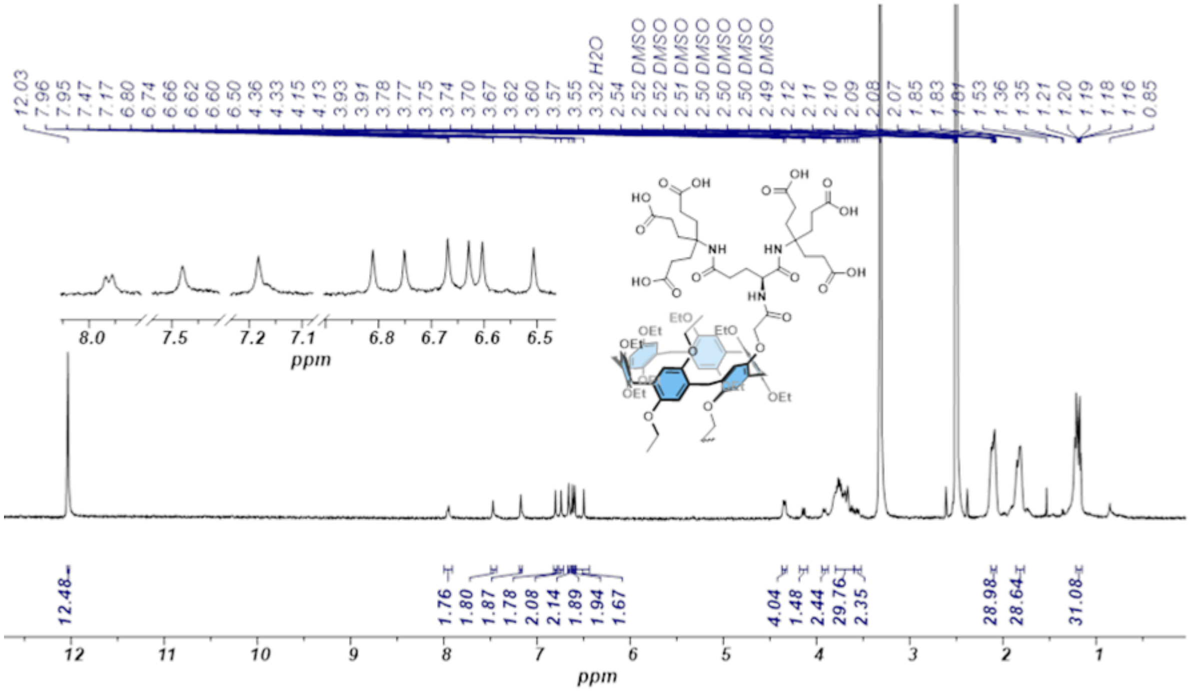

Figure S55. ^{1}H NMR spectrum (600 MHz, 298 K) of dendritic pillar[6]arene *pS*-**2** in DMSO-d_6.

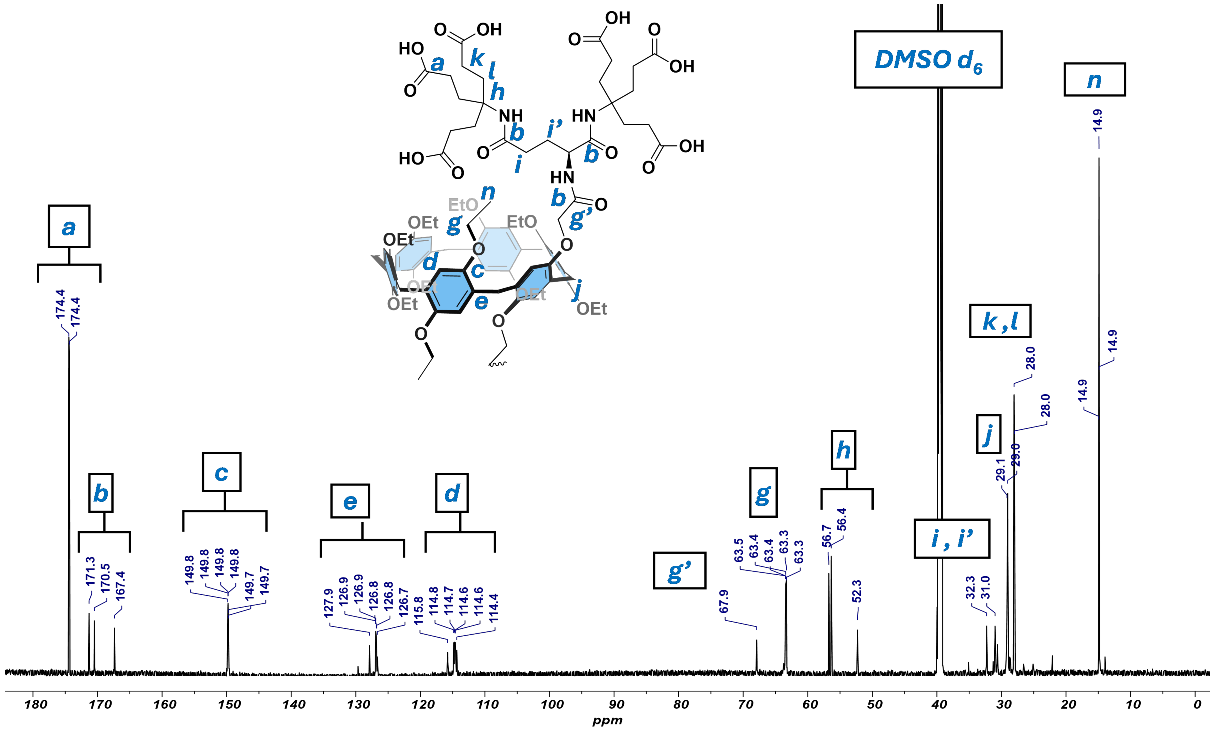

Figure S56. ^{13}C NMR spectrum (200 MHz, 298 K) of dendritic pillar[6]arene *pS*-**2** in DMSO-d_6.

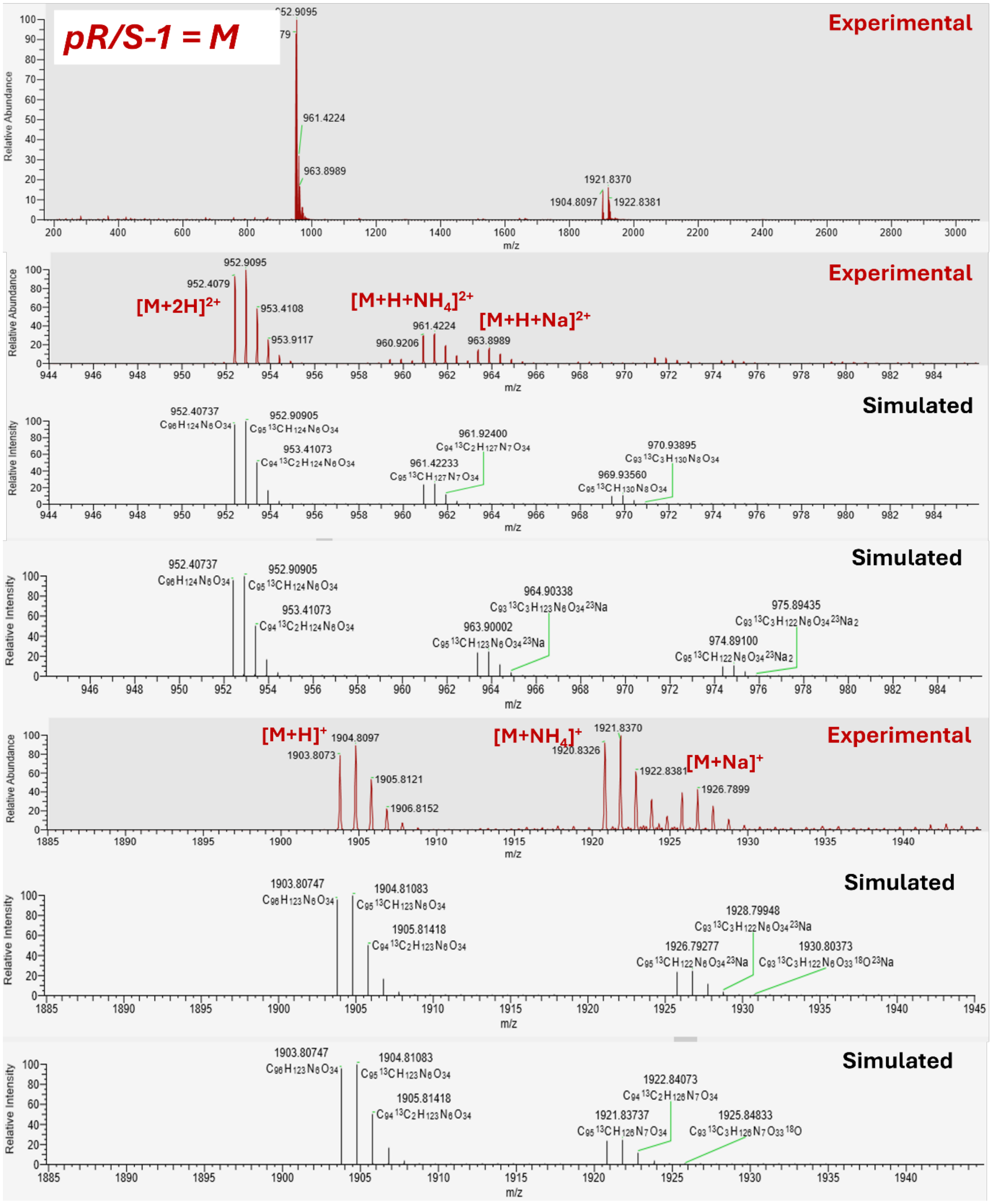

pR/S-1 = M
Experimental
Simulated
[M+2H]2+
[M+H+NH4]2+
[M+H+Na]2+
[M+H]+
[M+NH4]+
[M+Na]+
952.9095
961.4224
963.8989
1921.8370
1904.8097
1922.8381
952.4079
953.4108
953.9117
960.9206
952.40737
952.90905
953.41073
961.42233
961.92400
969.93560
970.93895
963.90002
964.90338
974.89100
975.89435
1903.8073
1905.8121
1906.8152
1920.8326
1926.7899
1903.80747
1904.81083
1905.81418
1926.79277
1928.79948
1930.80373
1921.83737
1922.84073
1925.84833
Relative Abundance
Relative Intensity
m/z

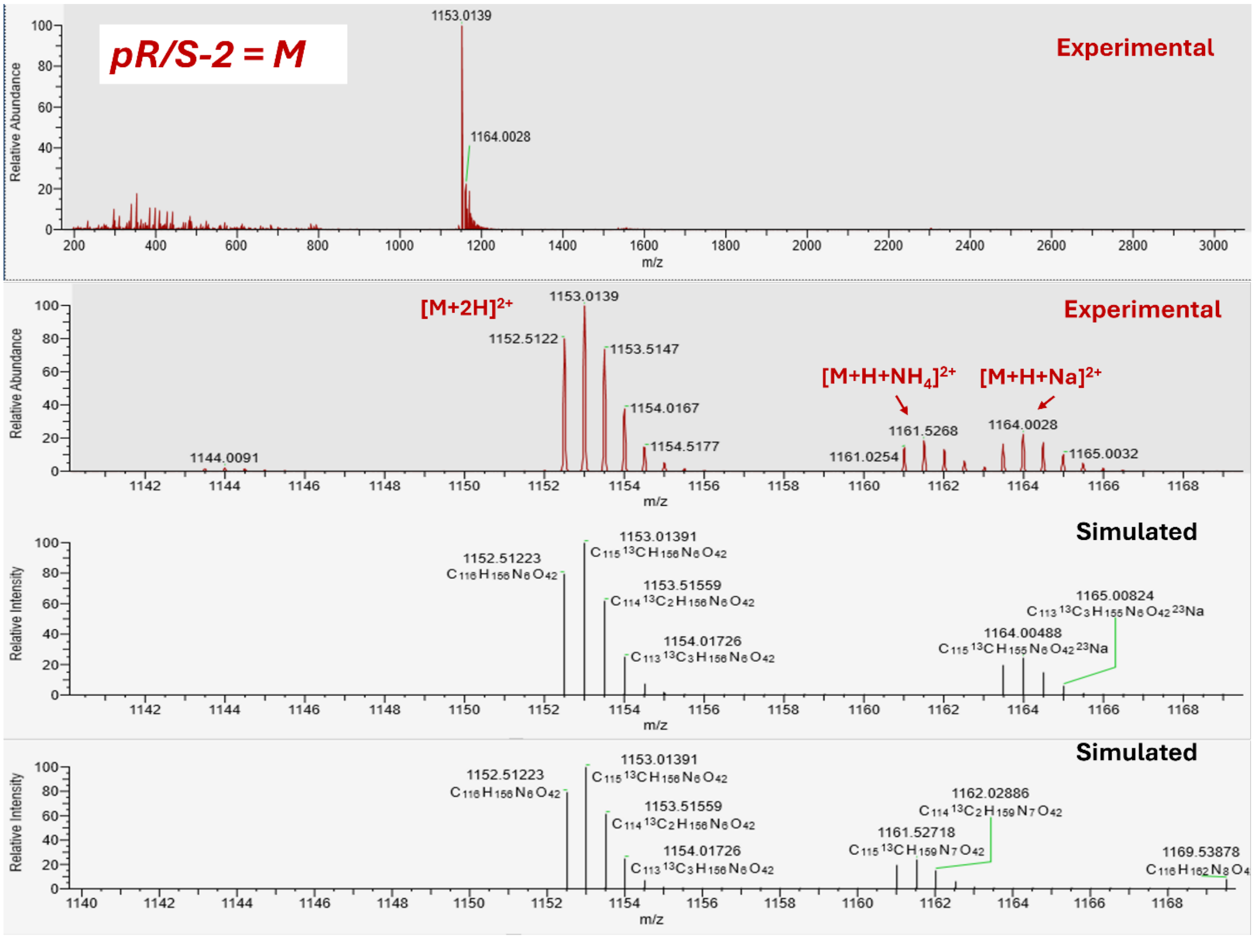

Figure S57. Experimental and simulated mass spectrometry (ESI-MS) data for dendritic *pR*/*pS*-**1** and *pR*/*pS*-**2** confirming their structure.

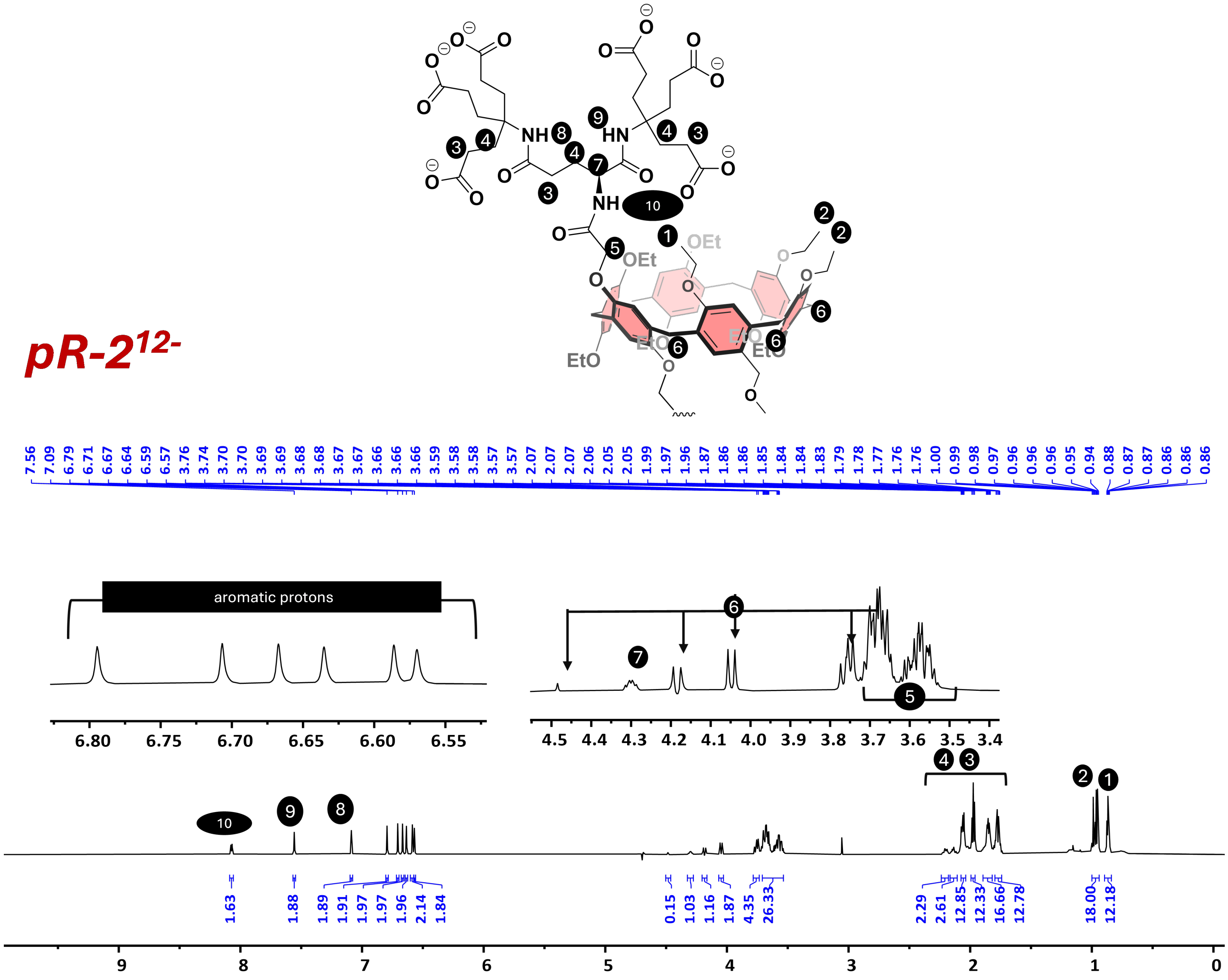

Figure S58. ^{1}H-NMR spectrum (600 MHz, 298 K) of dendritic pillar[6]arene *pR*-$\mathbf{2}^{12-}$ in 30 mM phosphate buffer at pH = 7.0 (10% D_2O) with dimethyl sulfone ($CH_3SO_2CH_3$) as an internal standard.

pS-2[12-]

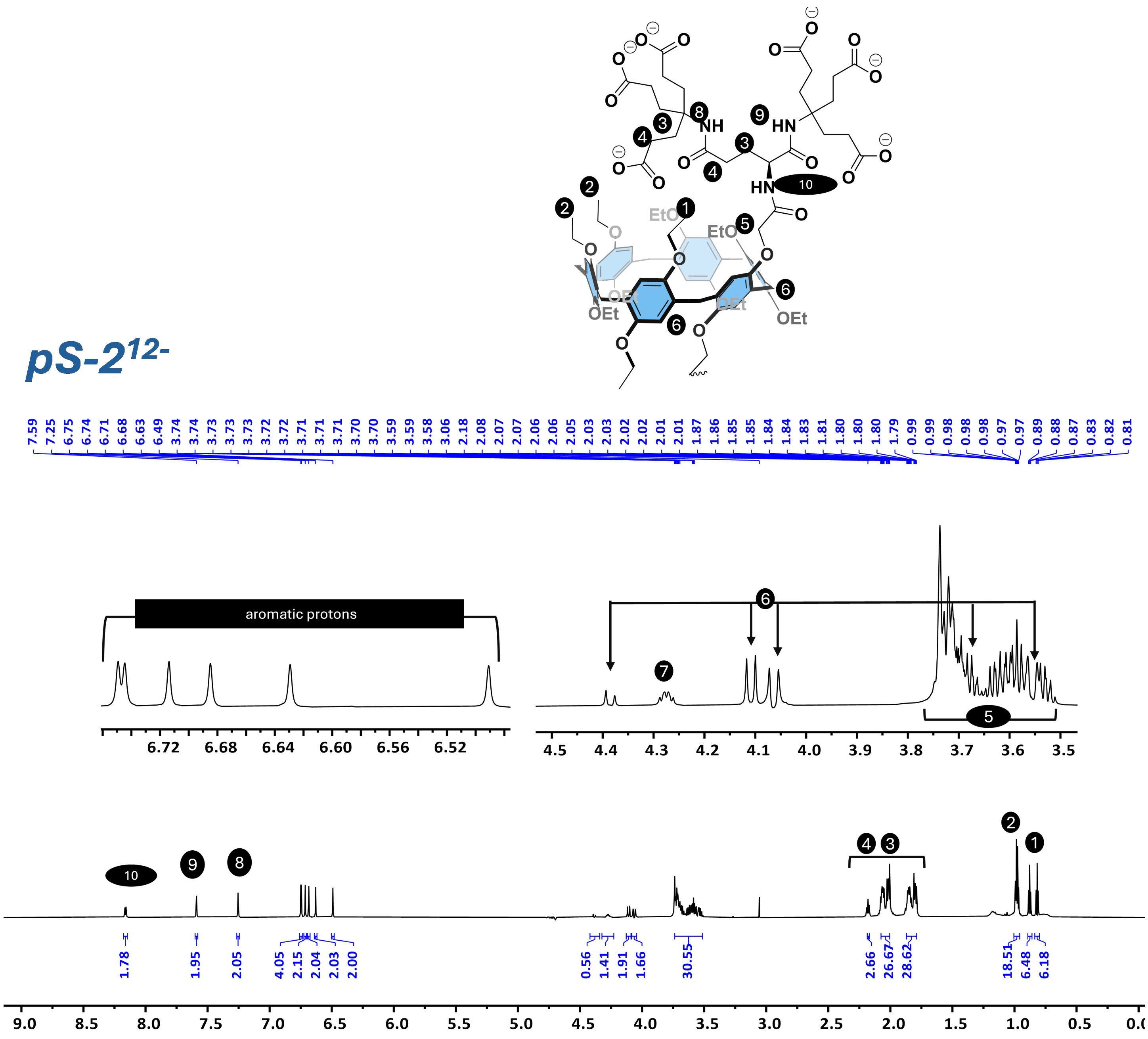

Figure S59. ^{1}H-NMR spectrum (600 MHz, 298 K) of dendritic pillar[6]arene *pS*-$\mathbf{2}^{12-}$ in 30 mM phosphate buffer at pH = 7.0 (10% D_2O) with dimethyl sulfone ($CH_3SO_2CH_3$) as an internal standard.

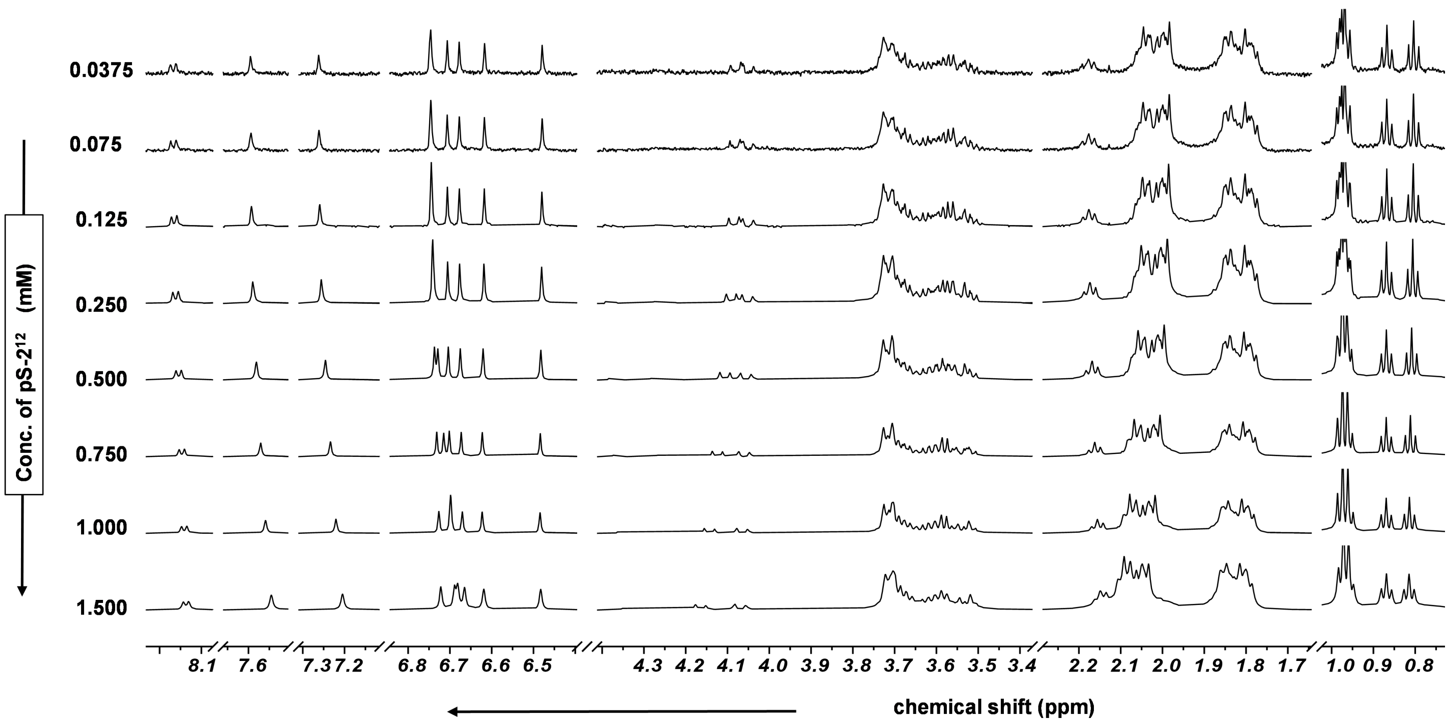

Figure S60. [1] H-NMR spectra (600 MHz, 298 K; water suppression) of 0.037-1.50 mM solutions of *pS-**2**$^{12-}$* in in 30 mM phosphate buffer at pH = 7.0 (10% D_2O). Dimethyl sulfone ($CH_3SO_2CH_3$) was used as an internal standard.

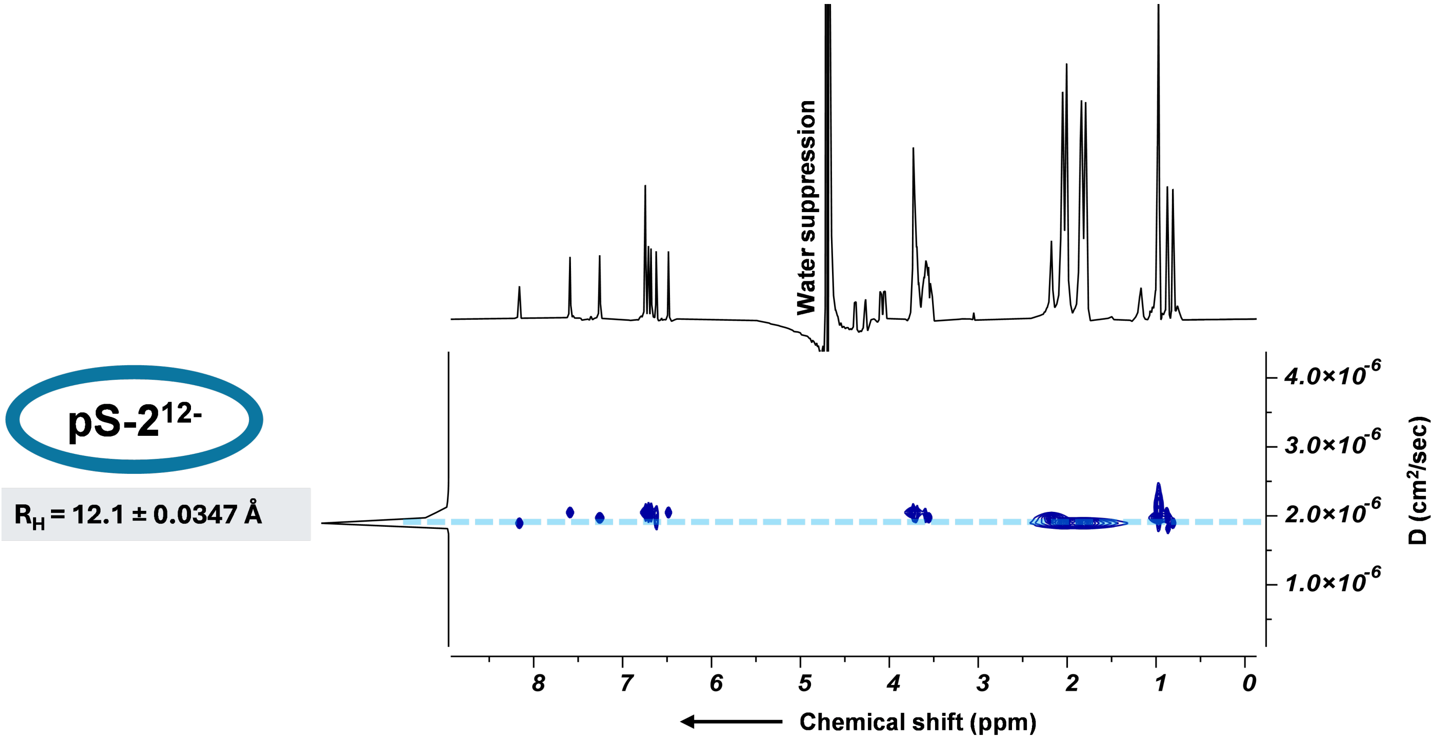

Figure S61. DOSY NMR spectrum (850 MHz, 298.0 K; water suppression) of dendritic pillar[6]arene *pS*-$\mathbf{2}^{12-}$ (0.25 mM) in 30 mM phosphate buffer at pH = 7.0 (10% of D_2O). Change in intensities of individual peaks with the magnetic field gradient strength were fitted to Stejskal-Tanner equation to obtain diffusion coefficient corresponding to each peak using Mnova NMR processing software by Bruker (Table S4). Hydrodynamic radius for *pS*-$\mathbf{2}^{12-}$ was R_H = 12.1 ± 0.4 Å, (mean ± standard deviation). It was calculated using the Stokes-Einstein equation for which the viscosity of water with 10% D_2O and at 298.0 K was taken as η = 0.911209 cP. Solvent peaks were excluded from the fitting.

Chemical Shift (ppm)	D(cm^2/sec)	Error (cm2/sec)	R_H(m)
8.16	1.93E-06	2.76E-08	1.24E-09
7.59	2.05E-06	2.55E-08	1.17E-09
7.26	2.00E-06	1.74E-08	1.20E-09
6.74	2.07E-06	3.12E-08	1.16E-09
6.71	2.04E-06	4.67E-08	1.17E-09
6.68	2.05E-06	3.82E-08	1.17E-09
6.62	1.99E-06	8.71E-08	1.20E-09
6.48	2.04E-06	3.18E-08	1.17E-09
3.73	2.05E-06	2.82E-08	1.17E-09
3.72	2.05E-06	2.38E-08	1.17E-09
3.71	1.97E-06	2.89E-08	1.22E-09
3.69	2.03E-06	5.10E-08	1.18E-09
3.60	2.01E-06	4.27E-08	1.19E-09
3.59	2.00E-06	4.20E-08	1.20E-09
3.56	1.98E-06	2.31E-08	1.21E-09
3.54	1.95E-06	3.19E-08	1.23E-09
2.18	1.95E-06	1.05E-08	1.23E-09
2.05	1.90E-06	7.17E-09	1.26E-09
2.01	1.93E-06	8.20E-09	1.24E-09
2.00	1.92E-06	3.60E-09	1.25E-09
1.84	1.90E-06	4.03E-09	1.26E-09
1.79	1.90E-06	8.24E-09	1.26E-09
0.98	1.97E-06	1.30E-08	1.22E-09
0.88	2.03E-06	1.50E-08	1.18E-09
0.87	1.96E-06	6.86E-09	1.22E-09
0.81	1.88E-06	2.36E-08	1.28E-09
		Average	1.21E-09
		Std dev	3.46898E-11

Table S4. Diffusion coefficients and hydrodynamic radii corresponding to DOSY NMR signals from dendritic pillar[6]arene *pS*-$\mathbf{2}^{12-}$.

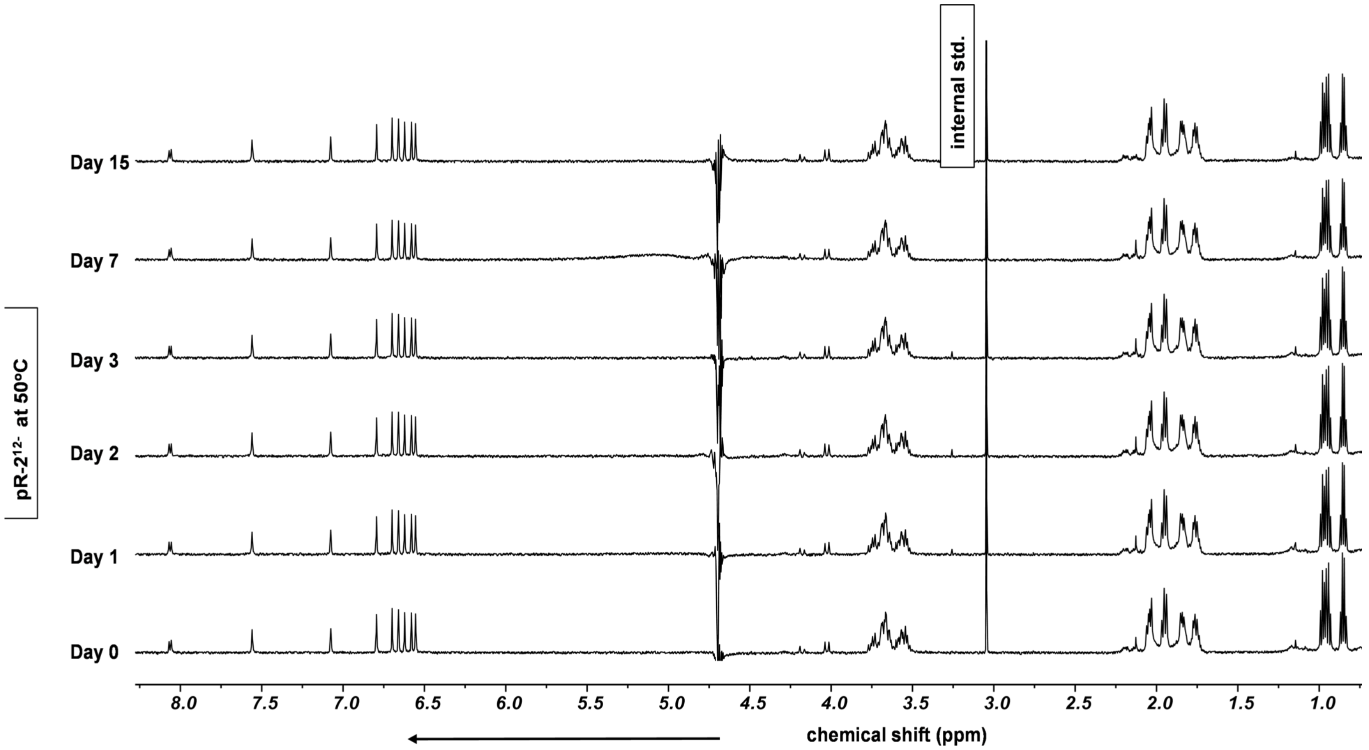
internal std.
Day 15
Day 7
Day 3
Day 2
Day 1
Day 0
pR-2[12-] at 50ºC
8.0 7.5 7.0 6.5 6.0 5.5 5.0 4.5 4.0 3.5 3.0 2.5 2.0 1.5 1.0
chemical shift (ppm)

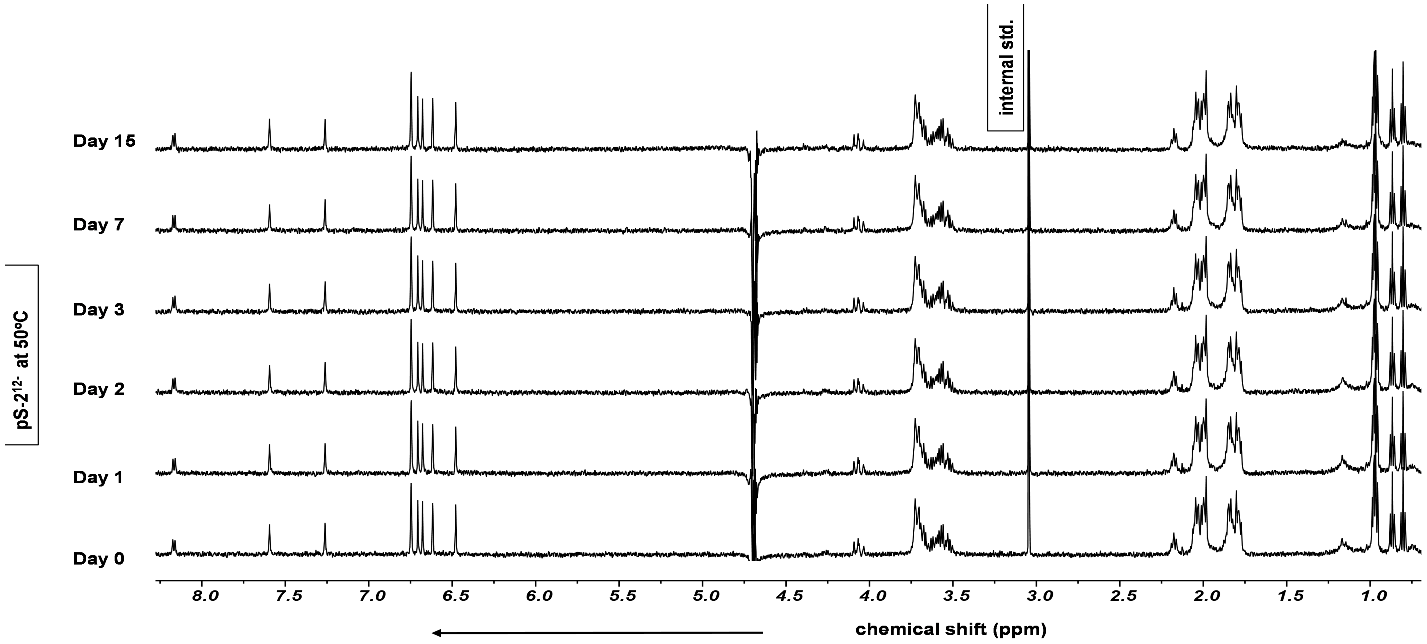
internal std.
Day 15
Day 7
Day 3
Day 2
Day 1
Day 0
pS-2[12-] at 50ºC
8.0 7.5 7.0 6.5 6.0 5.5 5.0 4.5 4.0 3.5 3.0 2.5 2.0 1.5 1.0
chemical shift (ppm)

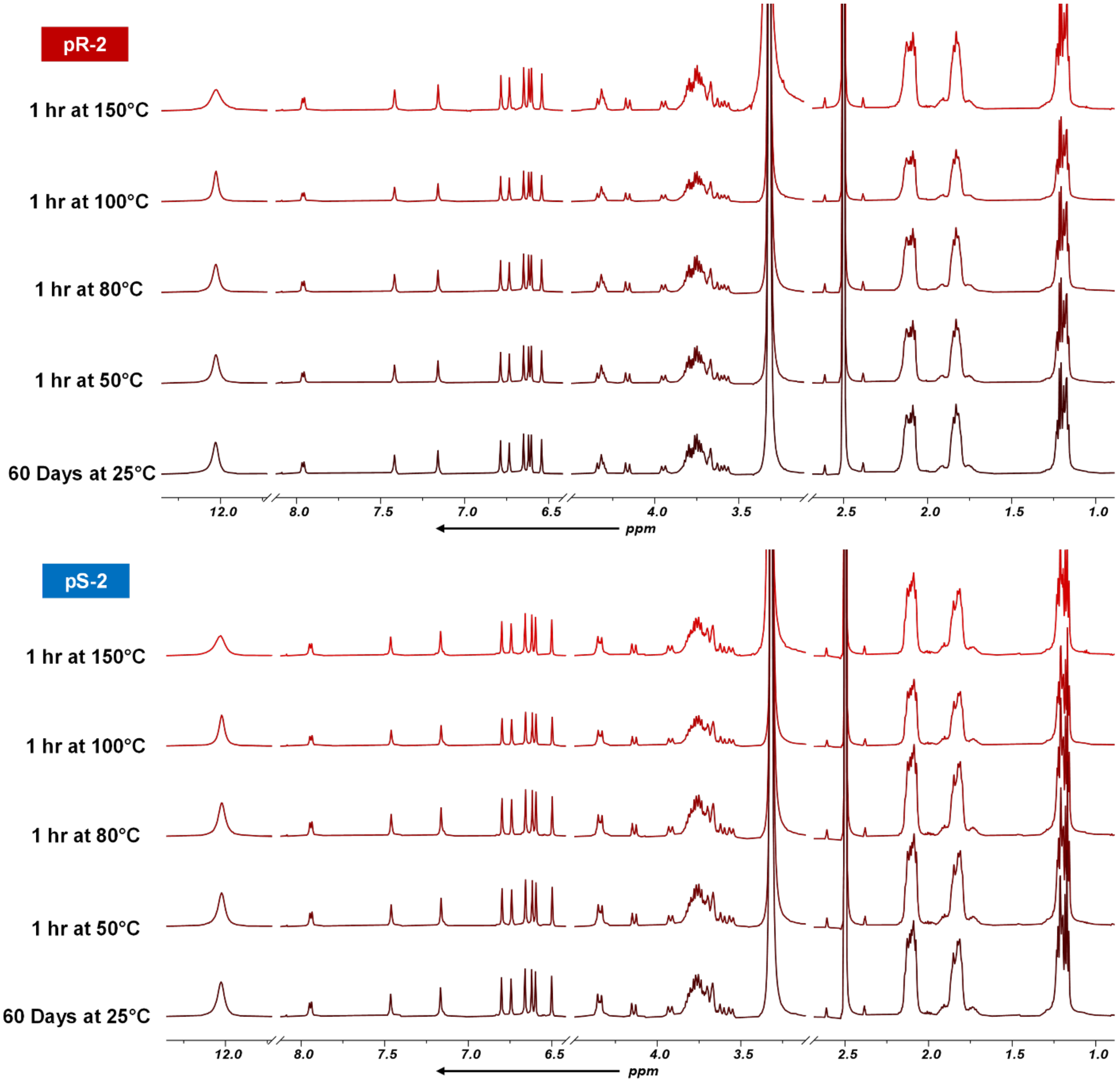

Figure S62. (Top two sets) ^{1}H-NMR spectra (600 MHz, water suppression) of dendritic pillar[6]arene *pR*-$\mathbf{2}^{12-}$ (0.1 mM) in 30 mM phosphate buffer at pH = 7.0 with (10% D_2O) recorded over two weeks with the solution kept at 50˚C. Dimethyl sulfone was used an internal standard. ^{1}H-NMR spectra (600 MHz, water suppression) of dendritic pillar[6]arene *pS*-$\mathbf{2}^{12-}$ (0.1 mM) in 30 mM phosphate buffer at pH = 7.0 with (10% D_2O) recorded over two weeks with the solution kept at 50ºC. Dimethyl sulfone was used an internal standard. (Bottom two sets) ^{1}H-NMR spectra (600 MHz at 298 K) of dendritic pillar[6]arenes *pR*–**2** (top) and *pS*–**2** (bottom) in DMSO-d_6. A solution of *pR*–**2** (top) and *pS*–**2** in DMSO were each stored for 60 days followed by heating at different temperatures for one hour. ^{1}H-NMR spectra were recorded at room temperature.

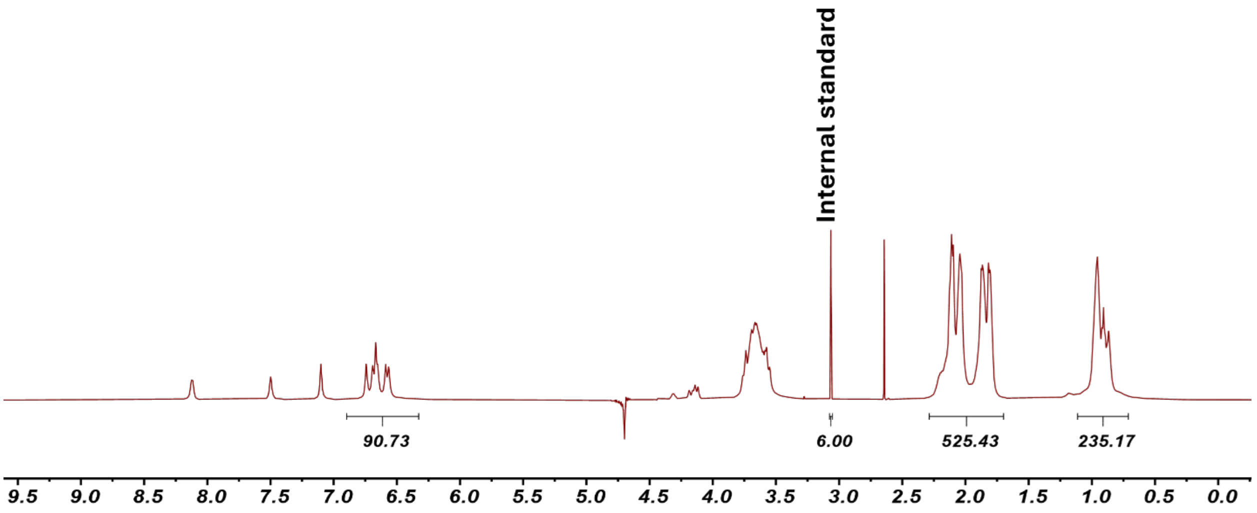

Figure S63. A saturated solution of dendritic pillar[6]arene *pR*–$\mathbf{2}^{12-}$ was prepared in 30 mM phosphate buffer at pH = 7.0 (10% D_2O). The solution contained 250 µM dimethyl sulfone ($CH_3SO_2CH_3$) as an internal standard. Quantitative ^{1}H-NMR spectrum (600 MHz, 298 K) of the solution was obtained using water suppression and Bruker pulse program zgesgp with relaxation time d_1 = 20 sec. From the integration ratios (i.e., internal standard versus cavitand), the concentration of *pR*–$\mathbf{2}^{12-}$ was found to be 2.01 mM. With identical negative charge and structural characteristics, we assume that *pS*–$\mathbf{2}^{12-}$ has comparable solubility in water.

Inclusion Complexation Studies of Levamisole (LEV$^+$) and Dexamisole (DEX$^+$) with Dendritic Pillar[6]Arenes *pS*-2^{12-} and *pR*-2^{12-}

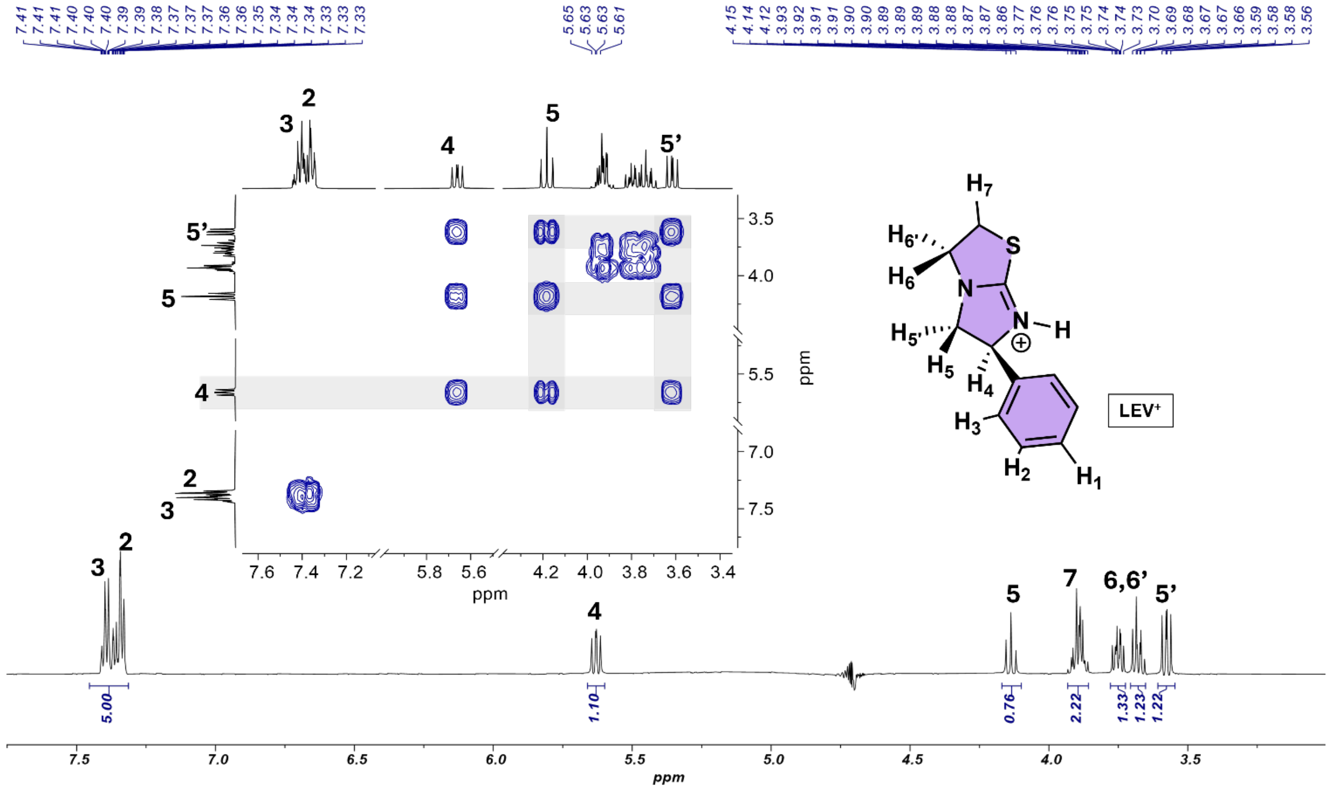

Figure S64. ^{1}H-NMR spectrum (600 MHz, 298 K; water suppression) of LEV$^+$ (0.25 mM) in 30 mM phosphate buffer (10% D_2O) at pH = 7.0. The inset is a segment of ^{1}H-^{1}H COSY NMR spectrum of LEV$^+$ in D_2O, with the cross peaks facilitating the assignment; note that resonances from H_1 are buried within multiplets assigned to H_2 and H_3 (7.3-7.5 ppm).

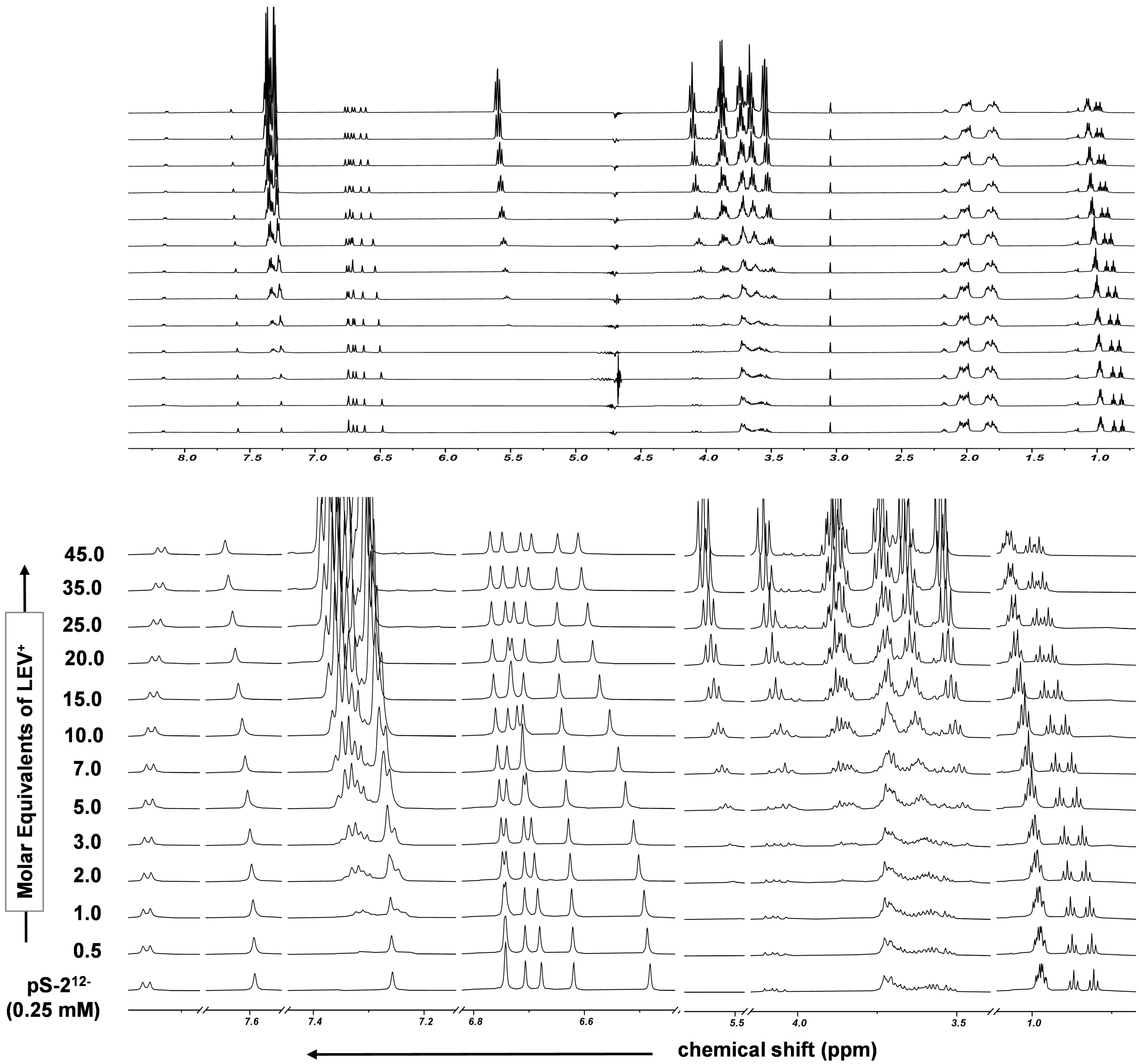

Figure S65. (Top) ^{1}H NMR spectra (600 MHz, 298 K; water suppression) of dendritic pillar[6]arene *pS*-$\mathbf{2}^{12-}$ (0.25 mM) obtained upon an incremental addition of 50 mM solution of LEV$^+$ in 30 mM phosphate buffer at pH = 7.0 (10% D_2O. (Bottom) Selected region of top ^{1}H NMR spectra are magnified for easier visualization.

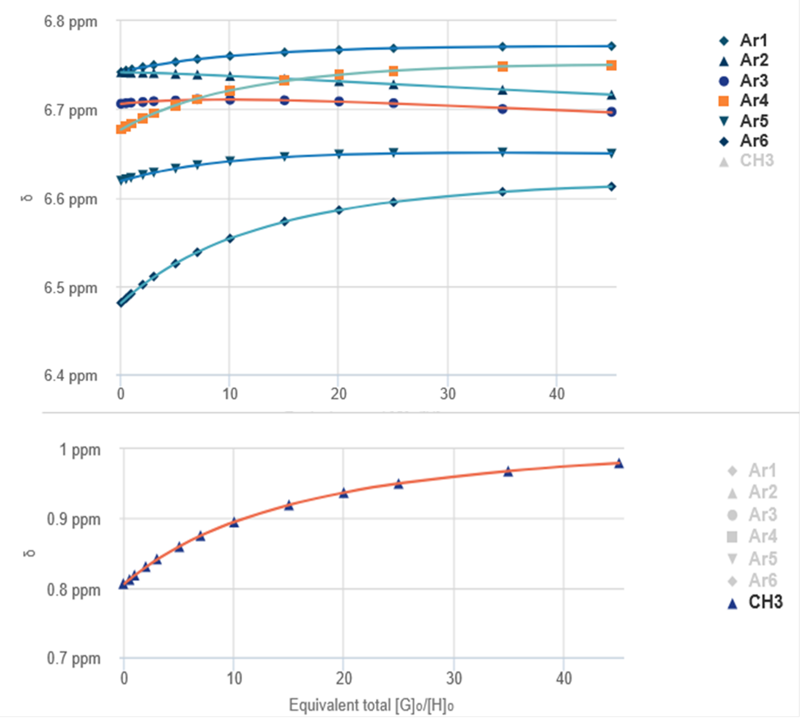

Fitter: NMR 1:2 | Fit | Summary | Save

Details

Time to fit 0.7424 s

SSR 7.2267e-6

Fitted datapoints 91

Fitted params 23

Parameters

Parameter (bounds)	Optimised	Error	Initial
K_{11} ($0 \rightarrow \infty$)	221.94 M^{-1}	± 0.3065 %	1000.00 M^{-1}
K_{12} ($0 \rightarrow \infty$)	22.02 M^{-1}	± 1.1394 %	100.00 M^{-1}

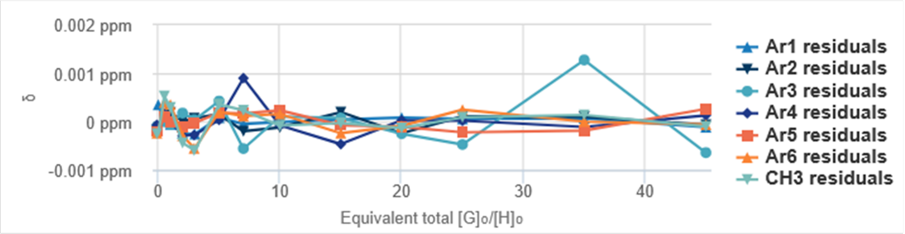

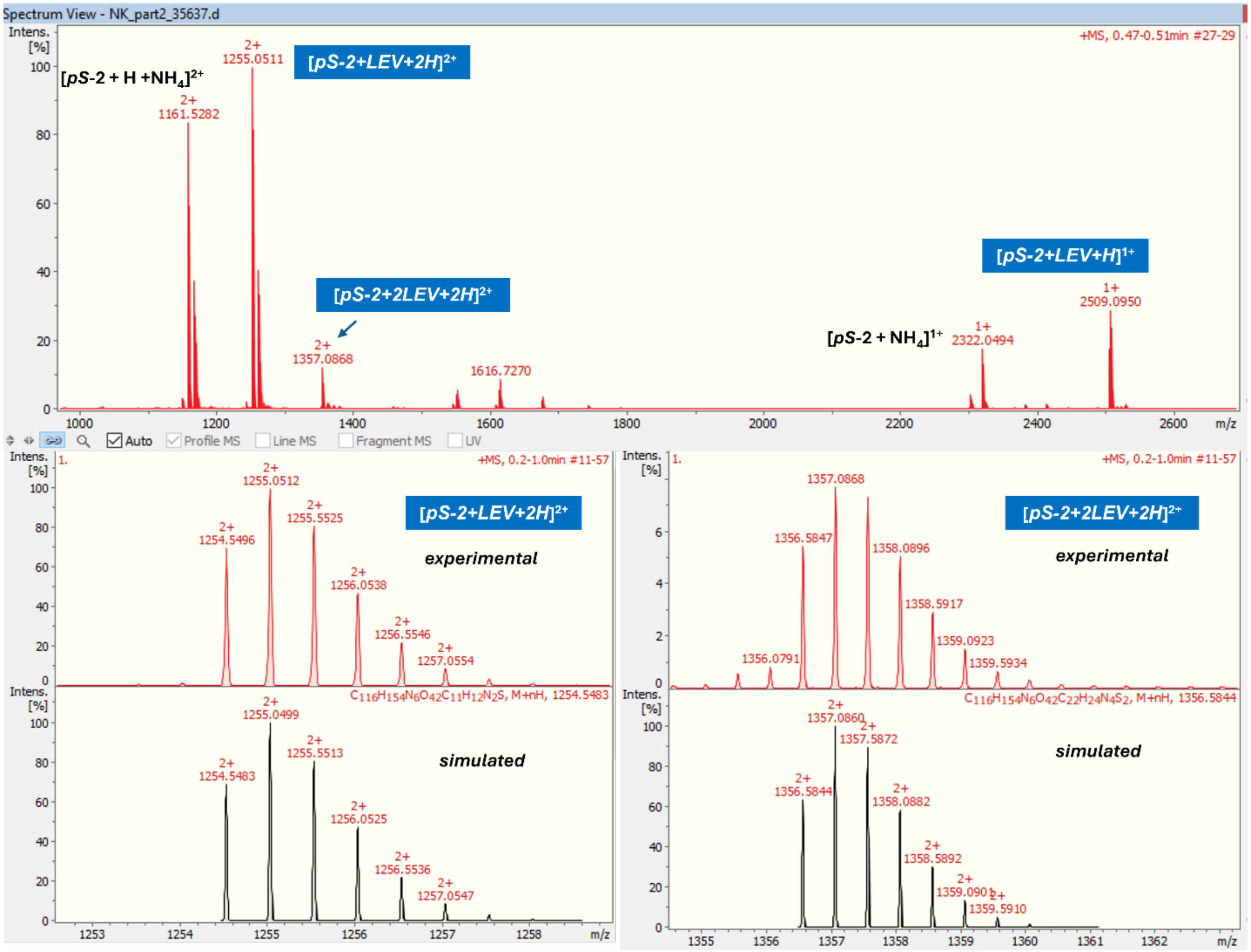

Figure S66. (Top) Using titration data from Figure S65, chemical shifts of seven signals from dendritic pillar[6]arene *pS*-**2**$^{12-}$ as a function of the increasing concentration of LEV^{+} were fit to 1-to-2 model of complexation (www.supramolecular.org) to give association constants $K_1 = 222 \pm 1$ M^{-1} and $K_2 = 22 \pm 1$ M^{-1} corresponding to the formation of binary [LEV⊂*pS*-**2**]$^{11-}$ and ternary [LEV$_2$⊂*pS*-**2**]$^{10-}$complexes. (Bottom) Experimental and simulated electron-spray ionization (ESI) data of 25 μM solution of *pS*–**2** containing ten molar equivalents of levamisole LEV^{+} in 25 mM ammonium bicarbonate buffer at pH = 7.0. Experimental and simulated signals corresponding to the formation of 1:1 complex [*pS*-**2**⊂LEV⊂2H]$^{2+}$ and 1:2 complex [*pS*-**2**⊂2LEV⊂2H]$^{2+}$ are at m/z = 1255.0512 and 1357.0868, respectively.

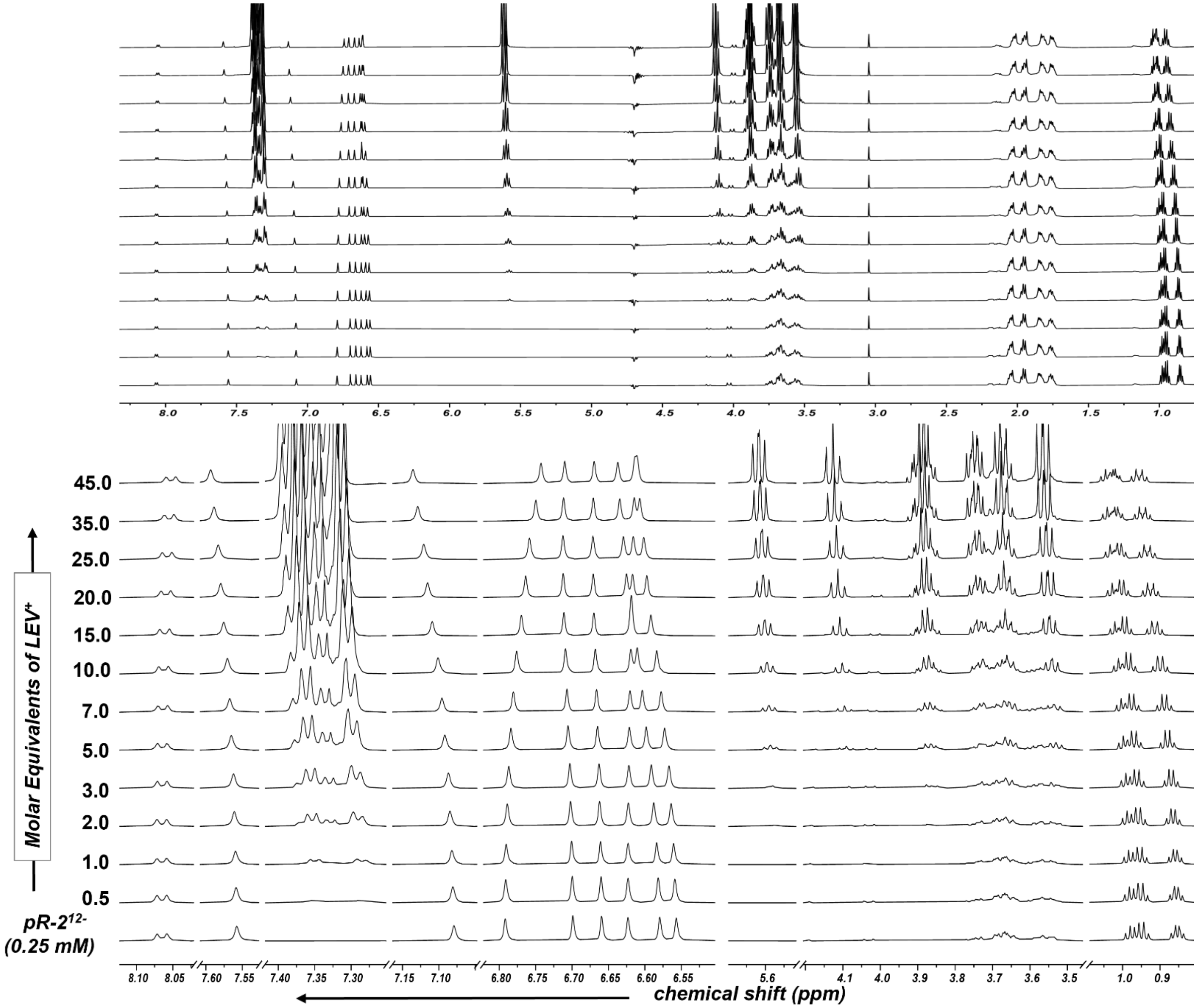

Figure S67: (Top) ^{1}H NMR spectra (600 MHz, 298 K; water suppression) of dendritic pillar[6]arene *pR*-$\mathbf{2}^{12-}$ (0.25 mM) were obtained upon an incremental addition of 50 mM solution of LEV^{+} in 30 mM phosphate buffer at pH = 7.0 (10% D_2O). (Bottom) Selected region of top ^{1}H NMR spectra are magnified for easier visualization.

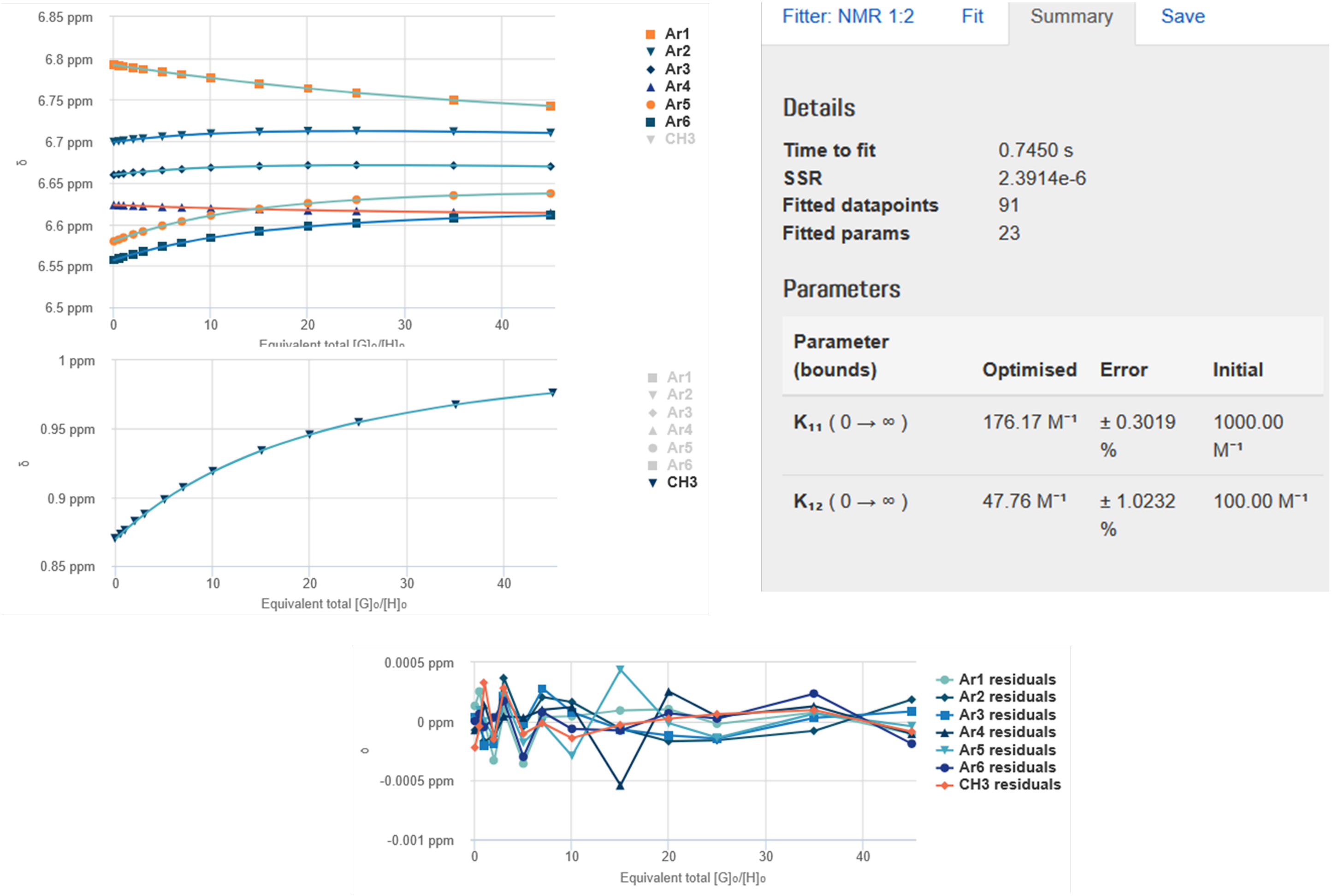

Figure S68. Using titration data from Figure S67, chemical shifts of seven signals from dendritic pillar[6]arene *pR*-**2**$^{12-}$ as a function of the increasing concentration of LEV^{+} were fit to 1-to-2 model of complexation (www.supramolecular.org) to give association constants $K_1 = 176 \pm 1$ M^{-1} and $K_2 = 48 \pm 1$ M^{-1} corresponding to the formation of binary [LEV⊂*pR*-**2**]$^{11-}$ and ternary [LEV$_2$⊂*pR*-**2**]$^{10-}$complexes.

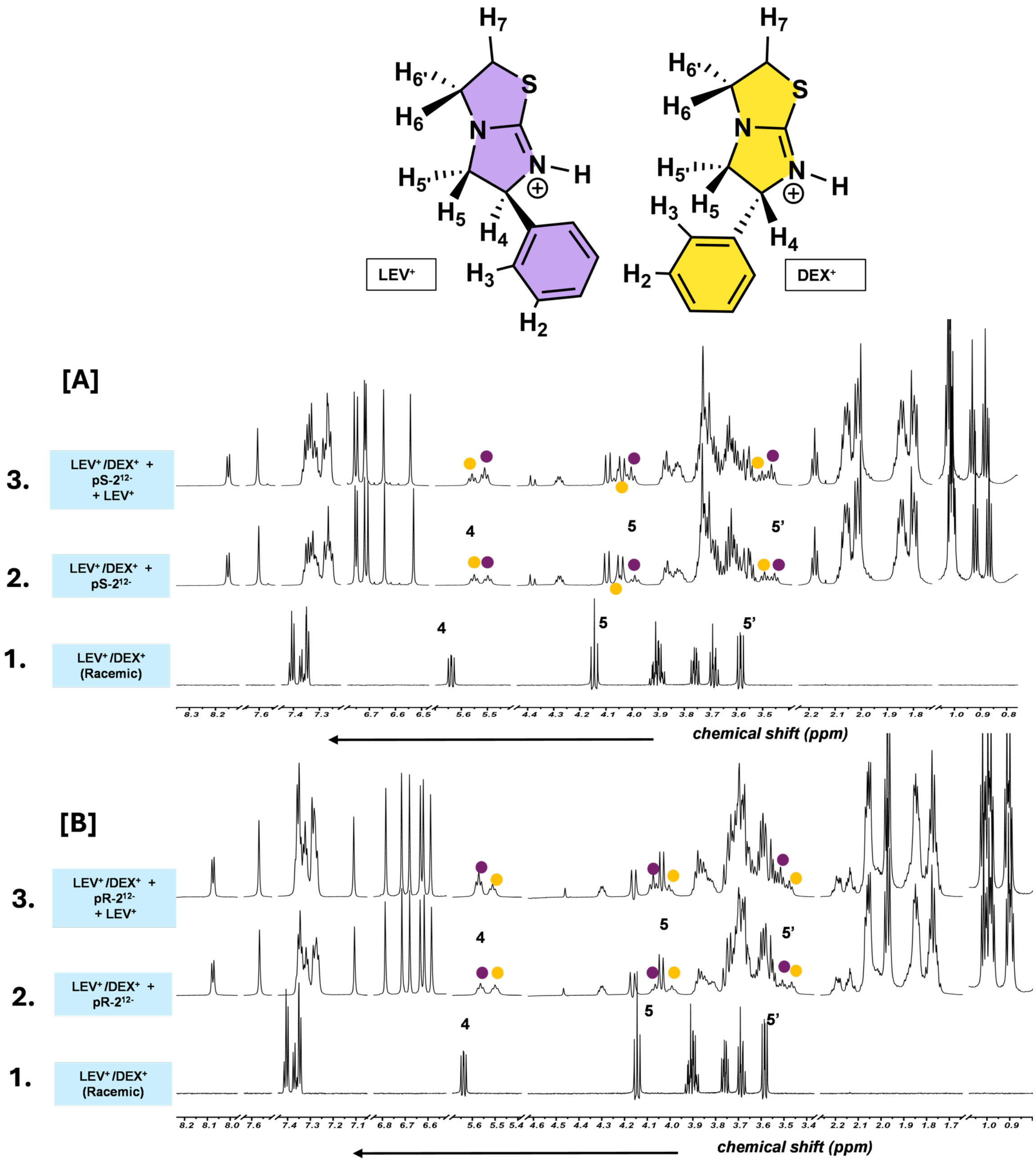

Figure S69. (A, bottom) ^{1}H NMR spectrum (850 MHz, 298.0 K; water suppression) of 0.5 mM of tetramisole (racemic LEV^+ and DEX^+) in 30 mM phosphate buffer at pH = 7.0. (A, middle) To a solution of tetramisole, one molar equivalent of *pS*-$\mathbf{2}^{12-}$ was added resulting in two distinct set of ^{1}H NMR resonances from the drug (yellow and magenta). (A, top) After spiking the sample with one molar equivalent of LEV^+, the intensity of more magnetically shielded magenta signals increased the intensity therefore corresponding to LEV^+. (B, bottom) ^{1}H NMR spectrum (850 MHz, 298.0 K; water suppression) of 0.5 mM of tetramisole (racemic LEV^+ and DEX^+) in 30 mM phosphate buffer at pH = 7.0. (B, middle) To a solution of tetramisole, one molar equivalent of *pR*-$\mathbf{2}^{12-}$ was added resulting in two distinct set of ^{1}H NMR resonances from the drug (yellow and magenta). (B, top) After spiking the sample with one molar equivalent of LEV^+, the intensity of less magnetically shielded magenta signals increased the intensity therefore corresponding to LEV^+.

Computational Study of [LEV⊂*pS*-**2**]$^{11-}$ Complex

For examining docking poses of levamisole (LEV+) in the cavity of dendritic pillar[6]arene *pS*-**2**$^{11-}$, we run Monte Carlo conformational search of [LEV⊂*pS*-**2**]$^{11-}$ inclusion complex (Maestro, Schrodinger). Note that for C_2 symmetric *pS*-**2**$^{11-}$, the docking of LEV^{+} to either side of its cylindrical pillar[6]arene cavity is equivalent. After docking the drug, the conformational search was initiated using OPLS4 force field (torsional sampling MCMM) and implicit water solvation. The maximum number of steps was set to 10000 with 300 steps per rotatable bond while the window was saving structures was 21 kJ/mol. The results are shown in Figure SX below.

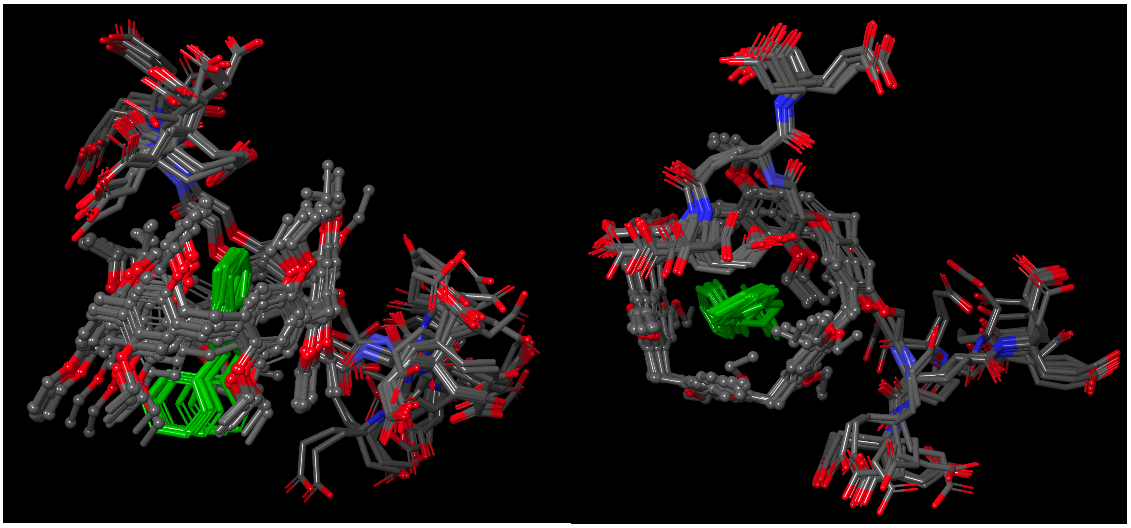

Row	In	Title	Stars	Added	Date Modified	Mark	Potential Energy-S-OPLS	Relative Potential Energy-S-OPLS	Source File	Source Path	Job Name
1	○	Structure1	☆☆☆	r 1…	17 Apr 14:52						
	15	mmod_csearch_54-out (597)									
2	●	Structure1	☆☆☆	r 0…	18 Apr 00:29		-5018.779	0.000	mmod_csea…	/Users/ba…	mmod_csearch_54
3	●	Structure1	☆☆☆	r 0…	18 Apr 00:29		-5018.515	0.264	mmod_csea…	/Users/ba…	mmod_csearch_54
4	●	Structure1	☆☆☆	r 0…	18 Apr 00:29		-5015.924	2.854	mmod_csea…	/Users/ba…	mmod_csearch_54
5	●	Structure1	☆☆☆	r 0…	18 Apr 00:29		-5015.559	3.220	mmod_csea…	/Users/ba…	mmod_csearch_54
6	●	Structure1	☆☆☆	r 0…	18 Apr 00:29		-5014.855	3.923	mmod_csea…	/Users/ba…	mmod_csearch_54
7	●	Structure1	☆☆☆	r 0…	18 Apr 00:29		-5014.702	4.077	mmod_csea…	/Users/ba…	mmod_csearch_54
8	●	Structure1	☆☆☆	r 0…	18 Apr 00:29		-5014.232	4.546	mmod_csea…	/Users/ba…	mmod_csearch_54
9	●	Structure1	☆☆☆	r 0…	18 Apr 00:29		-5014.033	4.746	mmod_csea…	/Users/ba…	mmod_csearch_54
10	●	Structure1	☆☆☆	r 0…	18 Apr 00:29		-5013.946	4.833	mmod_csea…	/Users/ba…	mmod_csearch_54
11	●	Structure1	☆☆☆	r 0…	18 Apr 00:29		-5013.783	4.996	mmod_csea…	/Users/ba…	mmod_csearch_54
12	●	Structure1	☆☆☆	r 0…	18 Apr 00:29		-5013.706	5.073	mmod_csea…	/Users/ba…	mmod_csearch_54
13	●	Structure1	☆☆☆	r 0…	18 Apr 00:29		-5013.690	5.088	mmod_csea…	/Users/ba…	mmod_csearch_54
14	●	Structure1	☆☆☆	r 0…	18 Apr 00:29		-5013.529	5.250	mmod_csea…	/Users/ba…	mmod_csearch_54
15	●	Structure1	☆☆☆	r 0…	18 Apr 00:29		-5013.413	5.366	mmod_csea…	/Users/ba…	mmod_csearch_54
16	●	Structure1	☆☆☆	r 0…	18 Apr 00:29		-5013.293	5.486	mmod_csea…	/Users/ba…	mmod_csearch_54
17	○	Structure1	☆☆☆	r 0…	18 Apr 00:29		-5013.167	5.612	mmod_csea…	/Users/ba…	mmod_csearch_54
18	○	Structure1	☆☆☆	r 0…	18 Apr 00:29		-5012.710	6.069	mmod_csea…	/Users/ba…	mmod_csearch_54
19	○	Structure1	☆☆☆	r 0…	18 Apr 00:29		-5012.641	6.138	mmod_csea…	/Users/ba…	mmod_csearch_54
20	○	Structure1	☆☆☆	r 0…	18 Apr 00:29		-5012.589	6.190	mmod_csea…	/Users/ba…	mmod_csearch_54
21	○	Structure1	☆☆☆	r 0…	18 Apr 00:29		-5012.397	6.381	mmod_csea…	/Users/ba…	mmod_csearch_54
22	○	Structure1	☆☆☆	r 0…	18 Apr 00:29		-5012.389	6.390	mmod_csea…	/Users/ba…	mmod_csearch_54
23	○	Structure1	☆☆☆	r 0…	18 Apr 00:29		-5012.171	6.607	mmod_csea…	/Users/ba…	mmod_csearch_54
24	○	Structure1	☆☆☆	r 0…	18 Apr 00:29		-5012.125	6.654	mmod_csea…	/Users/ba…	mmod_csearch_54
25	○	Structure1	☆☆☆	r 0…	18 Apr 00:29		-5012.104	6.675	mmod_csea…	/Users/ba…	mmod_csearch_54

Figure S70. (Top) Side and top views of energy minimized [LEV⊂*pS*-**2**]$^{11-}$ inclusion complex (OPLS4, Maestro). Sixteen most stable host-guest complexes are shown (hydrogens are removed for clarity); these complexes are within 5.486 kJ/mol (1.3 kcal/mol) window of relative steric energies. (Bottom) Table shows steric (potential) energies of first 25 out of 598 computed structures.

Dihedral Drive Scans of Dendritic Pillar[6]Arenes

1. Computational Methods

Dendritic pillar[6]arene containing single dendron **4** (akin to **M1**/**M2**, Figure S71A) was derived from the most stable pose of [LEV⊂*pS*-**2**]$^{11-}$ computed via a Monte Carlo conformational search in implicit water solvent (OPLS4).(1) The guest and one of the dendritic arms were removed, and then the carboxylate moieties were converted to methyl (**M1**, Figure S71A) or *tert*-butyl esters (**M2**, Figure S71a). Methyl esters were used as model compounds in order to avoid the formal charge, while not adding significant steric bulk. Additionally, the single dendritic pillar[6]arene containing single dendron **5** was drawn from the previous structure using GaussView6(2) to give **M3 (**Figure S71b) and **M4** (Figure S71b). Finally, both dendritic arms were removed and replaced with the corresponding ethoxy group to investigate the rotation about a single ring for the pillar[6]arene with 12 ethoxy groups (i.e., **EtP6**; **M5**, Figure S71c). All geometry optimizations and scans were performed using semi-empirical methods at the PM6 level of theory (Gaussian 16).(3)

2. Results

i. Geometry Optimization

We investigated pillar[6]arenes **M1**-**M5** (Figure S71), with **M1**-**M4** having single dendritic arm and **M5** being **EtP6** (Figure 1A in main text). **M1**-**M4** thus included methyl and *tert*-butyl esters of dendrons **4** (**M1** and **M2**, Figure S71a) and **5** (**M3** and **M4**, Figure S71b), while **M5** has twelve ethoxy groups (Figure S71c). Each structure was derived using GaussView6 from a Monte Carlo conformation search of [LEVpS-2]$^{11-}$ in implicit water solvent, using the OPLS4 molecular mechanics force field. Geometry optimizations were calculated at the semi-empirical PM6 level of theory, and four atoms used in the dihedral angle scan are shown in Figure S71 using cyan color.

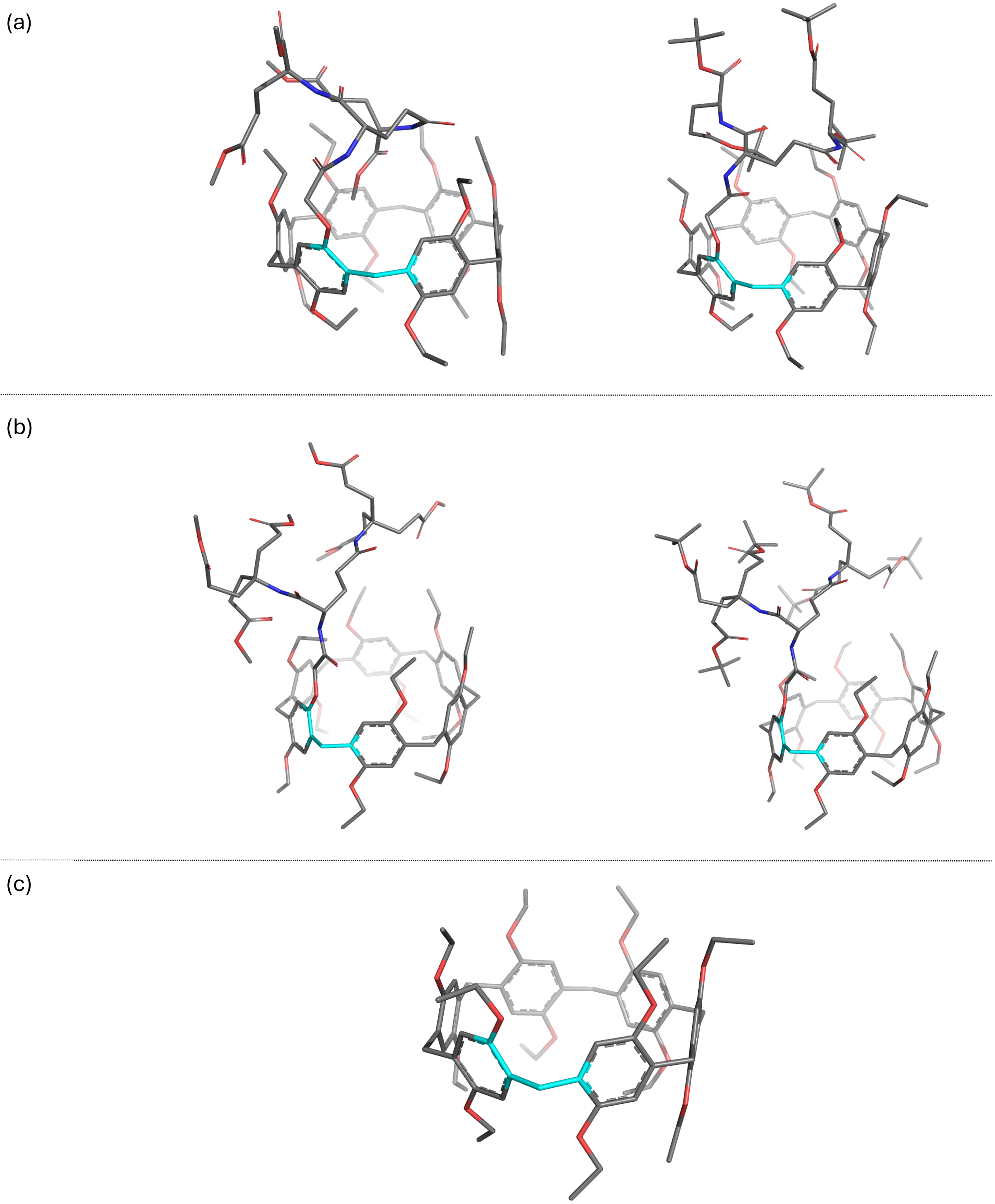

Figure S71. (a) Optimized structures of the methyl esters of **M1** (left) and the *tert*-butyl esters of **M2** (right). (b) Optimized structures of the methyl esters of **M3** (left) and the *tert*-butyl esters of **M4** (right). (c) Optimized structure of **M5**. All structures were computed at the PM6 level of theory. The four atoms used in dihedral scans are shown in cyan.

ii. Dihedral Angle Scans

The scan of 2,5-*bis*(ethoxy)pillar[6]arene EtP6 (**M5**) was set to 36 steps at dihedral $\varphi = 5°$ increments (180° total). It showed a 4.2 kcal/mol increase in energy over the first 50° before decreasing to 1.5 kcal/mol at $\varphi = 90°$, suggesting that, once the ethoxy group is inside the cavity, it can freely move. The energy remains approximately the same until reaching $\varphi = 170°$, where it drops to 0.9 kcal/mol. At $\varphi = 180°$, the energy of the system is slightly higher than the original value, likely due to the rotation of the other ethoxy groups and benzene rings. Initial and final structures, as well as a summary of the scan, are shown in Figure S72.

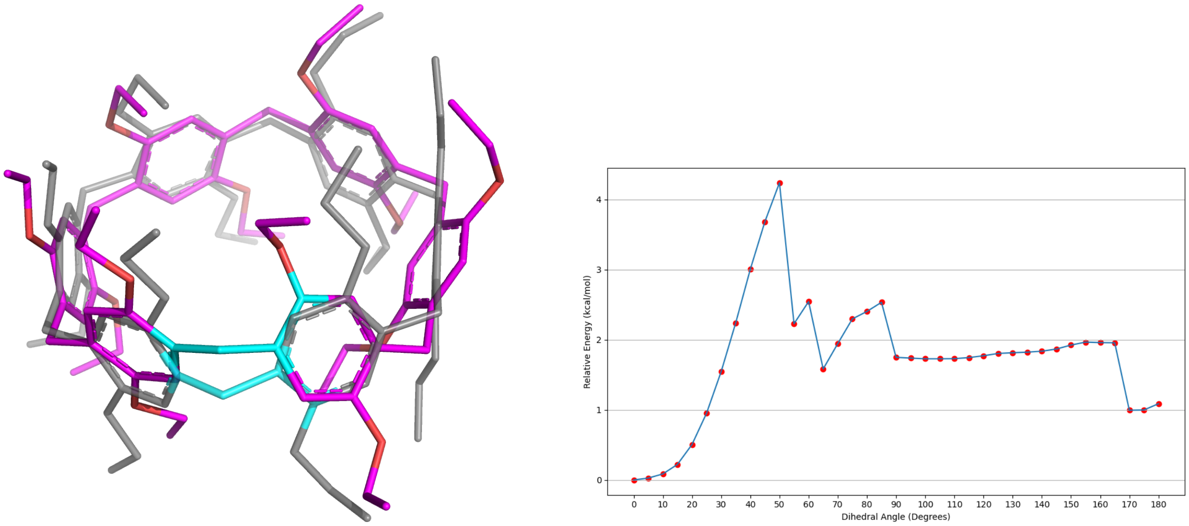

Figure S72. (Left) Overlay of initial (gray) and final (magenta) poses of 2,5-*bis*(ethoxy)pillar[6]arene **EtP6** (**M5**) from 180° dihedral scan (PM6). The four atoms depicting rotating dihedral angle φ used in calculation are shown in cyan. (Right) Potential energy diagram describing relative potential energy of **M5** as a function dihedral angle φ. The change in both dihedral angle φ and potential energy is relative to the initial position.

Moving from EtP6 (**M5**) to dendritic pillar[6]arenes **M1-M4**, we expected a substantial increase in the potential energy during the calculation due to increased steric bulk of dendrons being driven into the cylindrical cavity. For **M1**–**M4**, the scan was in each case set to 18 steps at $\varphi = 5°$ increments (90° total, Figure S73a). **M3** and **M4** reach 3.0 kcal/mol at a smaller dihedral angle than **M1** (40° and 55°, Figure S73a). Interestingly, **M3** and **M4** reach local minima at 45° and 65° (–1.9 and –1.6 kcal/mol, Figure S73a), suggesting the dendron is capable of stabilizing forces above the cavity. This can be seen to a greater extent with **M2**, reaching local minima at 25° and 55° (–5.3 and –8.2 kcal/mol, Figure S73a). However, this trend is not seen with **M1**; the energy continues to increase as the dihedral angle increases, likely due to the dendron being unable to thread through the cavity. Instead, the adjacent benzene ring and methylene twist to accommodate the changing dihedral angle, resulting in no rotation in the ring of interest (Figure S73b).

(a)

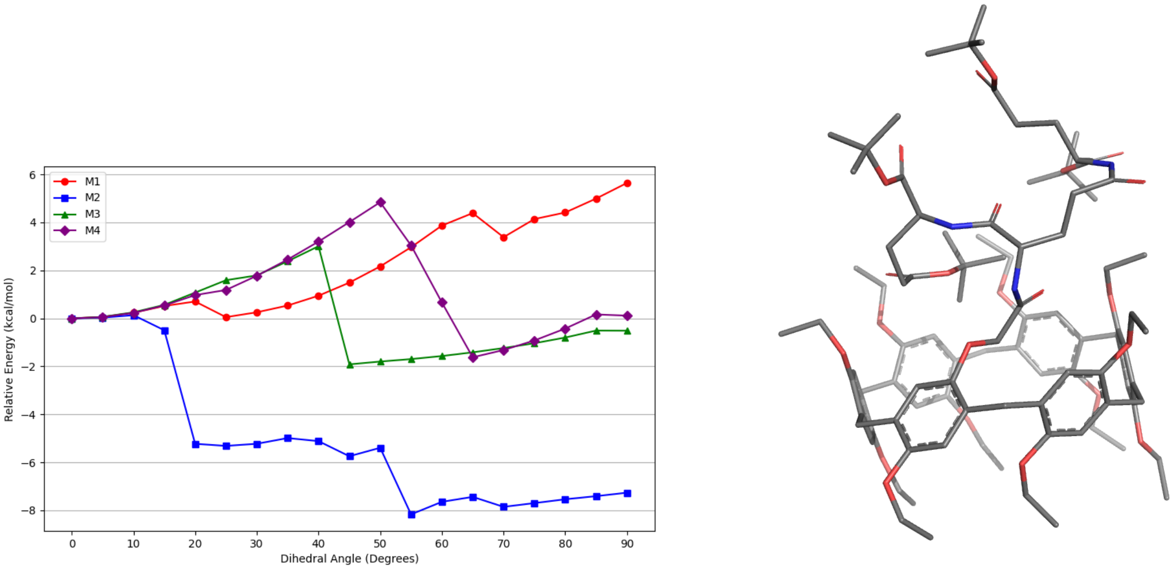

(b)

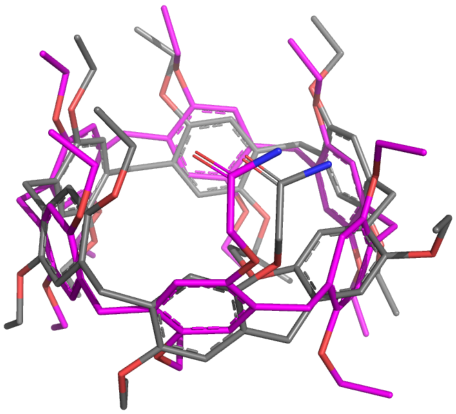

Figure S73. (a) (Left) Potential energy diagrams showing computed potential energies for **M1** (red), **M2** (blue), **M3** (green), and **M4** (purple) as a function of rotating dihedral angle φ. (Right) Step 12 (φ =55°) of dihedral drive scan for **M2** (PM6), showing the most stable position of the dendron. (b) Stick representations of initial and final poses of **M1** (PM6) shows significant distortion of the adjacent ring and methylene linker. The dendron is not shown for clarity.

Thus far, all scans have been performed in the "forward" direction – with the dendron going towards the cavity. However, when **11** undergoes *pS* to *pR* isomerization, one of the dendrons would be moving away from the cavity as the other moves in. Due to the simplified models, we decided to scan in the reverse direction to gain insight into what the other dendron may be doing. Interestingly, **M1** and **M2** have a steady increase by 5.0 and 4.2 kcal/mol from φ = 0° to –90°. At the same time, **M3** and **M4** reach minima at φ = –30° and –15° (–2.9 and –5.0 kcal/mol, respectively), suggesting that larger dendrons prefer twisting outward (Figure S74).

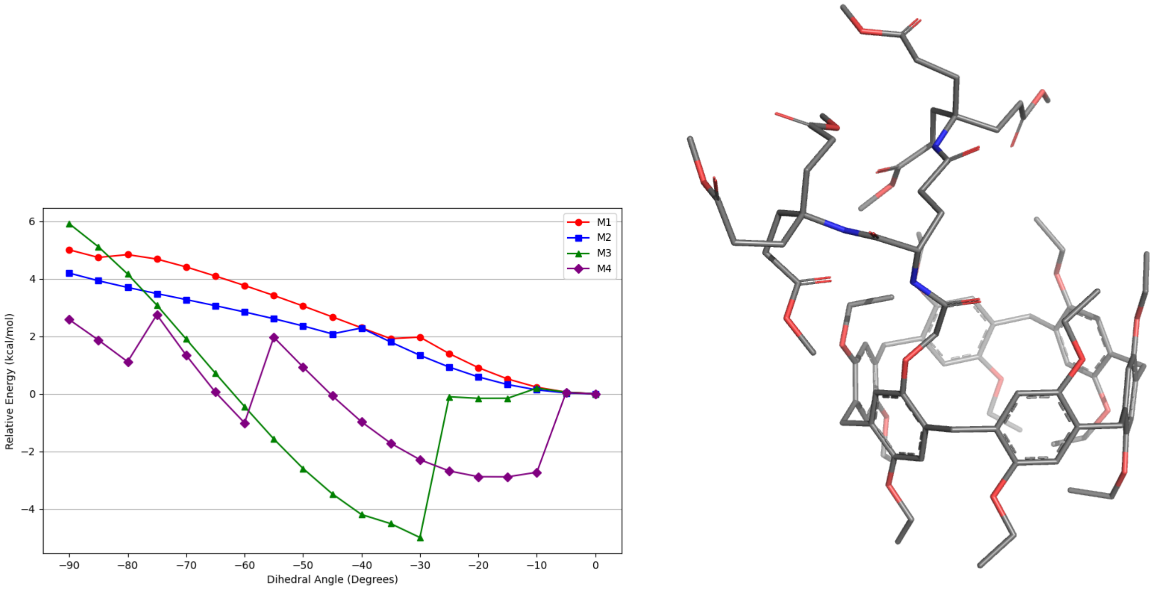

Figure S74. (Left) Scatter plot for the reverse scan of dihedral angle versus energy for **M1** (red), **M2** (blue), **M3** (green), and **M4** (purple). The change in angle and energy is relative to the initial position. (Right) Step 6 (30°) of dihedral scan for **M3** (PM6), showing the most stable position of the dendrimer.

3. Conclusion

To probe *in silico* *pS* to *pR* stereoisomerization of dendritic pillar[6]arenes *pS*/*pR*-**11** and *pS*/*pR*-**12**, we performed dihedral scan computations of five model compounds. These include methyl and *tert*-butyl esters of **M1** and **M2** (akin to *pS*/*pR*-**11**), **M3** and **M4** (akin to *pS*/*pR*-**12**) as well as **EtP6** labeled here as **M5**. Thus, **M1-M4** were unable to thread the dendron moiety through the cavity. In addition, dendrons tend to form favorable intermolecular contact above the pillararene cavity, making ring inversion less likely.

4. References

(1) Monte Carlo, OPLS4, Mendes, T., Sokal, A. D. Phys. Rev. D53 (1996) 3438-3444

(2) GaussView, Version 6, Dennington, Roy; Keith, Todd A.; Millam, John M. Semichem Inc., Shawnee Mission, KS, 2016.

(3) Gaussian 16, Revision C.01, Frisch, M. J.; Trucks, G. W.; Schlegel, H. B.; Scuseria, G. E.; Robb, M. A.; Cheeseman, J. R.; Scalmani, G.; Barone, V.; Petersson, G. A.; Nakatsuji, H.; Li, X.; Caricato, M.; Marenich, A. V.; Bloino, J.; Janesko, B. G.; Gomperts, R.; Mennucci, B.; Hratchian, H. P.; Ortiz, J. V.; Izmaylov, A. F.; Sonnenberg, J. L.; Williams-Young, D.; Ding, F.; Lipparini, F.; Egidi, F.; Goings, J.; Peng, B.; Petrone, A.; Henderson, T.; Ranasinghe, D.; Zakrzewski, V. G.; Gao, J.; Rega, N.; Zheng, G.; Liang, W.; Hada, M.; Ehara, M.; Toyota, K.; Fukuda, R.; Hasegawa, J.; Ishida, M.; Nakajima, T.; Honda, Y.; Kitao, O.; Nakai, H.; Vreven, T.; Throssell, K.; Montgomery, J. A., Jr.; Peralta, J. E.; Ogliaro, F.; Bearpark, M. J.; Heyd, J. J.; Brothers, E. N.; Kudin, K. N.; Staroverov, V. N.; Keith, T. A.; Kobayashi, R.; Normand, J.; Raghavachari, K.; Rendell, A. P.; Burant, J. C.; Iyengar, S. S.; Tomasi, J.; Cossi, M.; Millam, J. M.; Klene, M.; Adamo, C.; Cammi, R.; Ochterski, J. W.; Martin, R. L.; Morokuma, K.; Farkas, O.; Foresman, J. B.; Fox, D. J. Gaussian, Inc., Wallingford CT, 2016.

5. Supporting Tables

Table S5. Computed energies for dihedral drive scan of **M1.**

Dihedral Angle (Degrees)	Relative Dihedral Angle (Degrees)	Energy (Hartree)	Energy (kcal/mol)	Relative Energy (kcal/mol)
-207.6085	-90	-1.49638	-938.993	5.01
-202.6085	-85	-1.49680	-939.258	4.74
-197.6085	-80	-1.49665	-939.159	4.84
-192.6085	-75	-1.49689	-939.314	4.69
-187.6085	-70	-1.49733	-939.588	4.41
-182.6085	-65	-1.49783	-939.904	4.10
-177.6085	-60	-1.49836	-940.233	3.77
-172.6085	-55	-1.49891	-940.579	3.42
-167.6085	-50	-1.49949	-940.945	3.06
-162.6085	-45	-1.50009	-941.323	2.68
-157.6085	-40	-1.50070	-941.705	2.30
-152.6085	-35	-1.50130	-942.081	1.92
-147.6085	-30	-1.50122	-942.028	1.97
-142.6085	-25	-1.50213	-942.601	1.40
-137.6085	-20	-1.50291	-943.092	0.91
-132.6085	-15	-1.50354	-943.484	0.52
-127.6085	-10	-1.50399	-943.770	0.23
-122.6085	-5	-1.50427	-943.943	0.06
-117.6085	0	-1.50436	-944.001	0.00
-112.6085	5	-1.50427	-943.941	0.06
-107.6085	10	-1.50399	-943.766	0.24
-102.6085	15	-1.50354	-943.485	0.52
-97.6085	20	-1.50324	-943.297	0.70
-92.6085	25	-1.50428	-943.950	0.05
-87.6085	30	-1.50396	-943.747	0.25
-82.6085	35	-1.50351	-943.466	0.54
-77.6085	40	-1.50286	-943.057	0.94
-72.6085	45	-1.50199	-942.511	1.49
-67.6085	50	-1.50091	-941.834	2.17
-62.6085	55	-1.49964	-941.036	2.96
-57.6085	60	-1.49820	-940.133	3.87
-52.6085	65	-1.49737	-939.612	4.39
-47.6085	70	-1.49897	-940.617	3.38
-42.6085	75	-1.49776	-939.861	4.14
-37.6085	80	-1.49733	-939.588	4.41
-32.6085	85	-1.49640	-939.007	4.99
-27.6085	90	-1.49537	-938.357	5.64

Table S6. Computed energies for dihedral drive scan of **M2**.

Dihedral Angle (Degrees)	Relative Dihedral Angle (Degrees)	Energy (Hartree)	Energy (kcal/mol)	Relative Energy (kcal/mol)
-209.1631	-90	-1.64761	-1033.890	4.20
-204.1631	-85	-1.64803	-1034.157	3.93
-199.1631	-80	-1.64841	-1034.390	3.70
-194.1631	-75	-1.64875	-1034.604	3.49
-189.1631	-70	-1.64908	-1034.812	3.28
-184.1631	-65	-1.64942	-1035.024	3.07
-179.1631	-60	-1.64977	-1035.244	2.85
-174.1631	-55	-1.65014	-1035.477	2.61
-169.1631	-50	-1.65054	-1035.728	2.36
-164.1631	-45	-1.65098	-1036.004	2.09
-159.1631	-40	-1.65065	-1035.801	2.29
-154.1631	-35	-1.65143	-1036.289	1.80
-149.1631	-30	-1.65217	-1036.750	1.34
-144.1631	-25	-1.65282	-1037.159	0.93
-139.1631	-20	-1.65336	-1037.502	0.59
-134.1631	-15	-1.65379	-1037.768	0.32
-129.1631	-10	-1.65408	-1037.953	0.14
-124.1631	-5	-1.65425	-1038.058	0.03
-119.1631	0	-1.65430	-1038.090	0.00
-114.1631	5	-1.65425	-1038.058	0.03
-109.1631	10	-1.65409	-1037.956	0.13
-104.1631	15	-1.65509	-1038.584	-0.49
-99.1631	20	-1.66263	-1043.317	-5.23
-94.1631	25	-1.66277	-1043.405	-5.31
-89.1631	30	-1.66263	-1043.316	-5.23
-84.1631	35	-1.66225	-1043.078	-4.99
-79.1631	40	-1.66245	-1043.206	-5.12
-74.1631	45	-1.66346	-1043.836	-5.75
-69.1631	50	-1.66290	-1043.483	-5.39
-64.1631	55	-1.66732	-1046.258	-8.17
-59.1631	60	-1.66649	-1045.736	-7.65
-54.1631	65	-1.66616	-1045.529	-7.44
-49.1631	70	-1.66682	-1045.945	-7.85
-44.1631	75	-1.66657	-1045.789	-7.70
-39.1631	80	-1.66632	-1045.629	-7.54
-34.1631	85	-1.66611	-1045.500	-7.41
-29.1631	90	-1.66588	-1045.355	-7.26

Table S7. Computed energies for dihedral drive scan of **M3**.

Dihedral Angle (Degrees)	Relative Dihedral Angle (Degrees)	Energy (Hartree)	Energy (kcal/mol)	Relative Energy (kcal/mol)
-172.5182	-90	-1.83984	-1154.515	5.92
-167.5182	-85	-1.84112	-1155.320	5.11
-162.5182	-80	-1.84263	-1156.266	4.17
-157.5182	-75	-1.84435	-1157.348	3.08
-152.5182	-70	-1.84623	-1158.524	1.91
-147.5182	-65	-1.84812	-1159.713	0.72
-142.5182	-60	-1.84997	-1160.874	-0.44
-137.5182	-55	-1.85177	-1162.001	-1.57
-132.5182	-50	-1.85340	-1163.027	-2.59
-127.5182	-45	-1.85481	-1163.909	-3.48
-122.5182	-40	-1.85596	-1164.630	-4.20
-117.5182	-35	-1.85646	-1164.946	-4.51
-112.5182	-30	-1.85723	-1165.428	-5.00
-107.5182	-25	-1.84943	-1160.535	-0.10
-102.5182	-20	-1.84952	-1160.589	-0.16
-97.5182	-15	-1.84951	-1160.587	-0.15
-92.5182	-10	-1.84895	-1160.235	0.20
-87.5182	-5	-1.84918	-1160.376	0.06
-82.5182	0	-1.84927	-1160.433	0.00
-77.5182	5	-1.84917	-1160.370	0.06
-72.5182	10	-1.84887	-1160.180	0.25
-67.5182	15	-1.84837	-1159.871	0.56
-62.5182	20	-1.84756	-1159.358	1.07
-57.5182	25	-1.84673	-1158.842	1.59
-52.5182	30	-1.84641	-1158.642	1.79
-47.5182	35	-1.84547	-1158.052	2.38
-42.5182	40	-1.84448	-1157.426	3.01
-37.5182	45	-1.85232	-1162.349	-1.92
-32.5182	50	-1.85214	-1162.232	-1.80
-27.5182	55	-1.85197	-1162.130	-1.70
-22.5182	60	-1.85177	-1162.003	-1.57
-17.5182	65	-1.85152	-1161.848	-1.41
-12.5182	70	-1.85124	-1161.670	-1.24
-7.5182	75	-1.85092	-1161.469	-1.04
-2.5182	80	-1.85054	-1161.232	-0.80
2.4818	85	-1.85008	-1160.941	-0.51
7.4818	90	-1.85009	-1160.946	-0.51

Table S8. Computed energies for dihedral drive scan of **M4**.

Dihedral Angle (Degrees)	Relative Dihedral Angle (Degrees)	Energy (Hartree)	Energy (kcal/mol)	Relative Energy (kcal/mol)
-180.0979	-90	-2.09703	-1315.908	2.58
-175.0979	-85	-2.09817	-1316.618	1.87
-170.0979	-80	-2.09937	-1317.373	1.12
-165.0979	-75	-2.09677	-1315.741	2.75
-160.0979	-70	-2.09900	-1317.143	1.35
-155.0979	-65	-2.10105	-1318.431	0.06
-150.0979	-60	-2.10276	-1319.501	-1.01
-145.0979	-55	-2.09801	-1316.522	1.97
-140.0979	-50	-2.09966	-1317.556	0.93
-135.0979	-45	-2.10125	-1318.557	-0.07
-130.0979	-40	-2.10270	-1319.462	-0.97
-125.0979	-35	-2.10389	-1320.208	-1.72
-120.0979	-30	-2.10480	-1320.780	-2.29
-115.0979	-25	-2.10542	-1321.172	-2.68
-110.0979	-20	-2.10574	-1321.372	-2.88
-105.0979	-15	-2.10575	-1321.376	-2.89
-100.0979	-10	-2.10549	-1321.218	-2.73
-95.0979	-5	-2.10108	-1318.446	0.04
-90.0979	0	-2.10115	-1318.490	0.00
-85.0979	5	-2.10106	-1318.436	0.05
-80.0979	10	-2.10078	-1318.259	0.23
-75.0979	15	-2.10029	-1317.952	0.54
-70.0979	20	-2.09960	-1317.520	0.97
-65.0979	25	-2.09926	-1317.306	1.18
-60.0979	30	-2.09832	-1316.715	1.78
-55.0979	35	-2.09724	-1316.038	2.45
-50.0979	40	-2.09605	-1315.290	3.20
-45.0979	45	-2.09476	-1314.479	4.01
-45.0979	50	-2.09343	-1313.648	4.84
-45.0979	55	-2.09630	-1315.448	3.04
-45.0979	60	-2.10009	-1317.827	0.66
-45.0979	65	-2.10373	-1320.111	-1.62
-45.0979	70	-2.10325	-1319.809	-1.32
-45.0979	75	-2.10262	-1319.411	-0.92
-45.0979	80	-2.10183	-1318.915	-0.42
-45.0979	85	-2.10088	-1318.325	0.17
-45.0979	90	-2.10097	-1318.377	0.11

Table S9. Computed energies for dihedral drive scan of **M5**.

Dihedral Angle (Degrees)	Relative Dihedral Angle (Degrees)	Energy (Hartree)	Energy (kcal/mol)	Relative Energy (kcal/mol)
-100.9168	0	-0.70476	-442.245	0.00
-95.9168	5	-0.70472	-442.218	0.03
-90.9168	10	-0.70463	-442.160	0.09
-85.9168	15	-0.70441	-442.024	0.22
-80.9168	20	-0.70396	-441.740	0.51
-75.9168	25	-0.70325	-441.293	0.95
-70.9168	30	-0.70230	-440.701	1.54
-65.9168	35	-0.70119	-440.005	2.24
-60.9168	40	-0.69998	-439.241	3.00
-55.9168	45	-0.69889	-438.562	3.68
-50.9168	50	-0.69801	-438.007	4.24
-45.9168	55	-0.70121	-440.017	2.23
-40.9168	60	-0.70071	-439.701	2.54
-35.9168	65	-0.70224	-440.662	1.58
-30.9168	70	-0.70166	-440.301	1.94
-25.9168	75	-0.70110	-439.948	2.30
-20.9168	80	-0.70093	-439.841	2.40
-15.9168	85	-0.70072	-439.711	2.53
-10.9168	90	-0.70198	-440.496	1.75
-5.9168	95	-0.70199	-440.507	1.74
-0.9168	100	-0.70201	-440.517	1.73
4.0832	105	-0.70201	-440.517	1.73
9.0832	110	-0.70201	-440.515	1.73
14.0832	115	-0.70198	-440.500	1.75
19.0832	120	-0.70194	-440.473	1.77
24.0832	125	-0.70189	-440.441	1.80
29.0832	130	-0.70187	-440.431	1.81
34.0832	135	-0.70186	-440.422	1.82
39.0832	140	-0.70184	-440.409	1.84
44.0832	145	-0.70179	-440.377	1.87
49.0832	150	-0.70170	-440.321	1.92
54.0832	155	-0.70163	-440.279	1.97
59.0832	160	-0.70164	-440.286	1.96
64.0832	165	-0.70164	-440.288	1.96
69.0832	170	-0.70318	-441.251	0.99
74.0832	175	-0.70317	-441.247	1.00
79.0832	180	-0.70303	-441.158	1.09
